# The Preparation of Diaryl Sulfoxinium Triflates and Their Application in Palladium‐Catalyzed Cross‐Coupling Reactions

**DOI:** 10.1002/asia.202200828

**Published:** 2022-08-29

**Authors:** Chenyang Wang, Xianliang Wang, Deshen Kong, Khai‐Nghi Truong, Kari Rissanen, Carsten Bolm

**Affiliations:** ^1^ Institute of Organic Chemistry RWTH Aachen University Landoltweg 1 52074 Aachen Germany; ^2^ University of Jyvaskyla Department of Chemistry FI-40014 Jyväskylä Finland

**Keywords:** Coupling reaction, Onium salt, Sulfoximine, Sulfoxinium, Triflate

## Abstract

Treatment of *N*‐methyl‐*S*,*S*‐diaryl sulfoximines with methyl trifluoromethanesulfonate provides bench‐stable sulfoxinium salts in excellent yields. Applying them in Sonogashira‐, Heck‐ and Suzuki‐type cross‐coupling reactions leads to the corresponding products by sequential C−S bond cleavage and C−C bond formation. Electronic factors induced by substituents on the *S*‐aryl groups govern the coupling efficiency.

## Graphical abstract

***N,N′*** **‐Dimethyl‐*S,S*‐diaryl oxosulfonium triflates** are easily prepared from the corresponding sulfoximines by treatment with methyl trifluoromethanesulfonat. In palladium‐catalyzed Sonogashira‐, Heck‐ and Suzuki‐type cross‐coupling reactions, such salts can be applied a readily available aryl source. High aryl transfer selectivities have been observed in competition experiments.

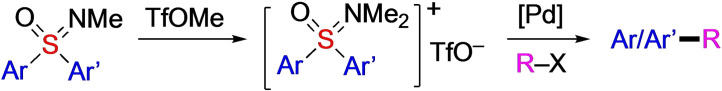

Sulfoximines were discovered by Bentley and co‐workers in the late 1940‐ies.^[1]^ Since then, these tetracoordinated sulfur(VI) reagents have extensively been investigated finding applications in organic synthesis, medicinal chemistry, and crop protection.[^2^, ^3^, ^4^] As part of his seminal work in this area, Johnson and co‐workers prepared a series of oxosulfonium salts **1** (Figure 1 and demonstrated the use of the corresponding ylides in methylene transfer reactions leading to epoxides, aziridines, and cyclopropanes.^[5]^ This chemistry was extended by Shibata and co‐workers, who demonstrated transfers of fluorinated groups R_f_ from reagents such as **2**.[^6^, ^7^] Magnier and co‐workers reported an analogous behavior of oxosulfonium salts **2** with fluorodichloro‐, bromodifluoro‐, and trifluoromethyl substituents.^[8]^

**Figure 1:**
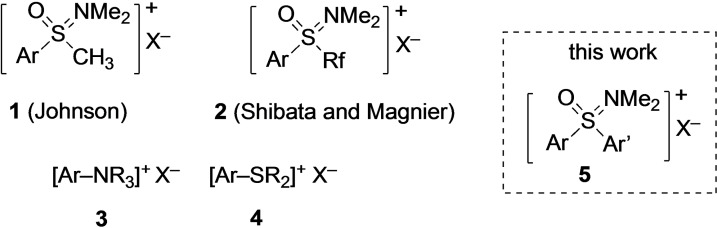
Oxosulfonium salts and related onium reagents with various synthetic applications.

After the initial finding of cross‐couplings with ammonium salts **3** by Wenkert and co‐workers in 1988,^[9]^ the field has been advanced to nowadays allow efficient C−C‐ and C−X‐bond formations under both metal‐catalyzed and metal‐free conditions.[^10^, ^11^] Almost a decade later, Liebeskind and co‐workers demonstrated the applicability of sulfonium salts **4** in such reactions,^[12]^ resulting in synthetic opportunities with tremendous synthetic potential for organic chemistry.^[13]^ In light of those results, we started wondering about transition metal‐catalyzed aryl‐transfer options with onium reagents of type **5** leading to the formation of new C−C bonds. The validation of this idea and the success of this project are reported here.

The project started with the preparation of a series of *N,N*‐dimethyl‐*S*,*S*‐diaryl oxosulfonium salts **5**, which were obtained by treatment of the corresponding sulfoximines **6** with methyl trifluoromethanesulfonate. Performing the reactions in acetonitrile at room temperature led to the targeted products **5** in yields ranging from 50 to 97% (Scheme 1). The respective structures were either symmetric (with two identical aryl groups, **5** **a**–**f**), unsymmetric (with two different aryls, **5** **g**–**k**), or fused (**5** **l**). The structure of **5** **a** was unequivocally confirmed by single crystal X‐ray structure determination (see Supporting Information for details).

**Scheme 1:**
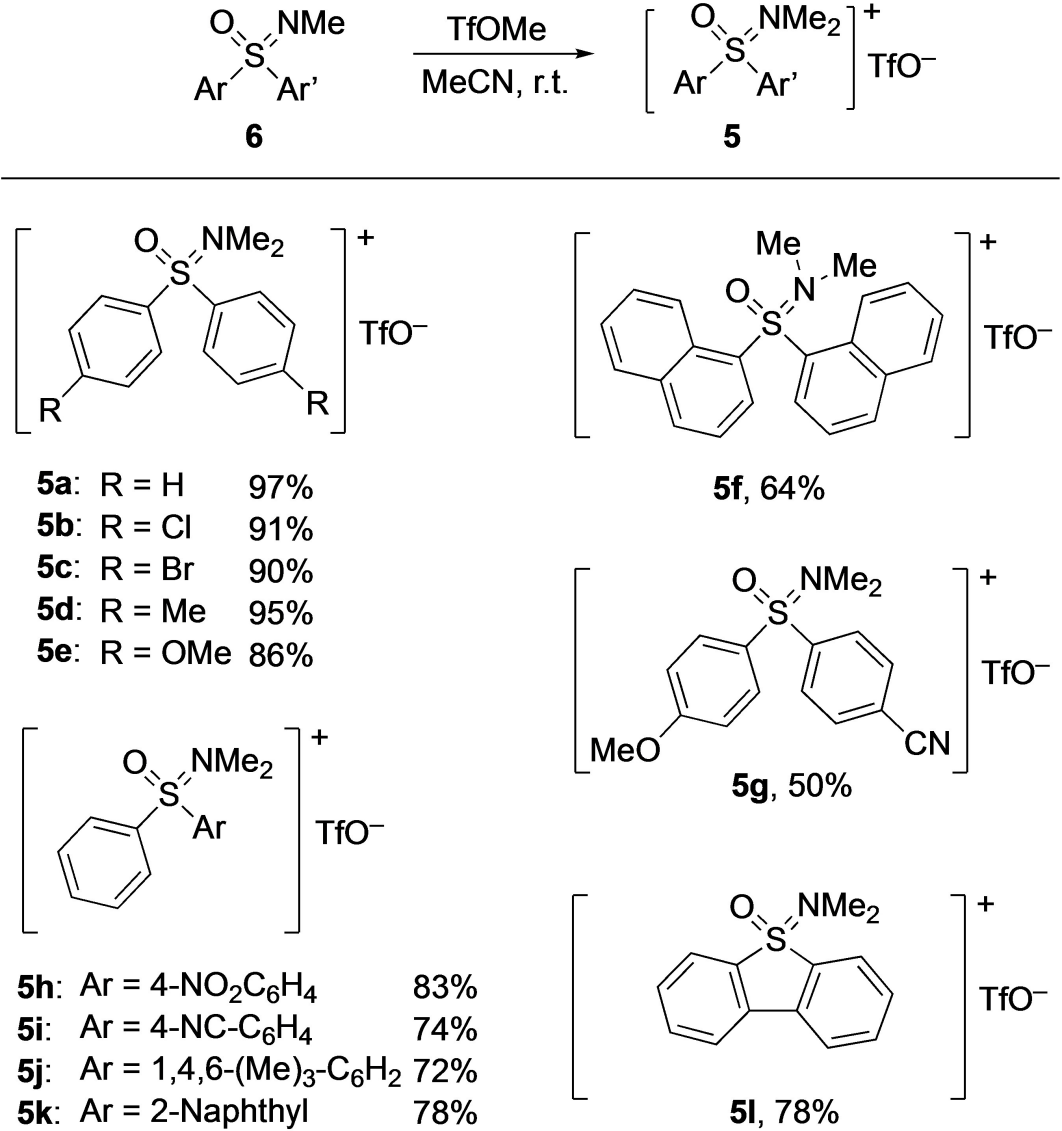
Preparation of *N,N*‐dimethyl‐*S*,*S*‐diaryl oxosulfonium triflates **5**.

For evaluating the potential of the envisaged cross‐coupling approach, a Sonogashira type C−C‐bond formation was investigated first. The initial screening and reaction optimization was performed with diphenyl sulfoxonium triflate **5** **a** and *p*‐tolyacetylene (**7** **a**) as representative substrates. The results are summarized in Table 1.

**Table 1:** Optimization of the reaction conditions of the Sonogashira‐type coupling reaction.^[a]^

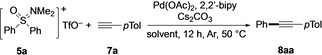		
Entry	Deviation from standard conditions	Yield [%]^[b]^
1	None	99 (94)
2	DCE instead of DCM	75
3	MeCN instead of DCM	56
4	Toluene instead of DCM	40
5	THF instead of DCM	91
6	DMF instead of DCM	99
7	Without 2,2’‐bipyridine	21
8	r.t. instead of 50 °C	65

[a] Use of **5** **a** (0.2 mmol), **7** **a** (0.4 mmol, 2.0 equiv.), Pd*(*OAc)_2_ (2.5 mol %), 2,2’‐bipyridine (10 mol %), and Cs_2_CO_3_ (0.4 mmol, 2.0 equiv.) in 1.5 mL of the solvent at 50 °C under argon. After 12 h, the solvent was evaporated, and the product was purified by column chromatography. 2,2’‐bipy=2,2’‐bipyridine, DCM=dichloromethane, DCE=1,2‐dichlorethane, THF=tetrahydrofuran, DMF=dimethylformamide [b] Determined by ^1^H NMR analysis of the crude reaction mixture using CH_2_Br_2_ as the internal standard. Yield of isolated product in parentheses.

Pleasingly, our hypothesis was confirmed, and coupling product **8** **aa** was formed in 99% yield (as determined by NMR spectroscopy with dibromomethane as internal standard), when a catalyst derived from 2.5 mol % of Pd(OAc)_2_ and 10 mol % of 2,2’‐bipyridine were applied in combination with 2 equiv. of Cs_2_CO_3_ in DCM at 50 °C under argon for 12 h (Table 1, entry 1). After aqueous work‐up followed by column chromatography, **8** **aa** was obtained in 94% yield. Substituting DCM by DCE, acetonitrile, toluene, or THF had a negative effect on the yield of **8** **aa** (Table, entries 2–5). DMF proved to be a suitable solvent too (Table 1, entry 6), but considering the product work‐up procedure, using DCM appeared advantageous. Performing the catalysis without 2.2’‐bipyridine or at ambient temperature, reduced the yield of **8** **aa** to 21% and 65%, respectively (Table 1, entries 7 and 8).

The substrate scope of the Sonogashira‐type coupling with oxosulfonium salts **5** and terminal alkynes **7** was broad, and the products were generally obtained in good to excellent yields (Scheme 2). In the first series, salts **5** **a**–**d**, and **5** **f** were reacted with *p*‐tolyl‐substituted alkyne **7** **a**. In general, the yields of the corresponding coupling products were high ranging from 82% (for **8** **ba**) to 94% (for **8** **aa**). Presumably due to competing cross‐couplings at the bromine sites, salt **5** **c** did not react well leading to a complex mixture of unidentified products. Electronic effects induced by aryl substituents appeared to be of minor importance. This notion was supported by the results of reactions between oxosulfonium salt **5** **b** and alkynes **7** **b**–**f**. In these cases, the yields of **8** **bb**–**bf** ranged from 65–96%. Now, 4‐methoxy‐ and 4‐fluoro‐substituted arylalkynes **7** **b** and **7** **c**, representing substrates with an electron‐donating and an electron‐withdrawing substituent, respectively, gave almost identical yields for the corresponding products (**8** **bb**: 85% and **8** **bc**: 87%). Moving the fluoro substituent from the 4 to the 3 and 2 positions of the aryl had a negative effect on the product yield as shown by the data for **8** **bd** (65%) and **8** **be** (67%) compared to **8** **bc** (87%). Particularly pleasing was the result for 1‐hexyne (**7** **f)**, which coupled with **5** **b** to give **8** **bf** in 96% yield.

**Scheme 2:**
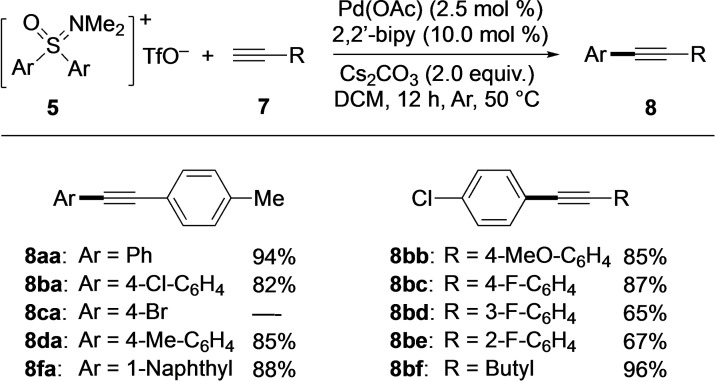
Substrate scope of Sonogashira‐type coupling reactions with oxosulfonium salts **5** and terminal alkynes **7**.

Next, Heck‐type reactions were tested, using diaryl sulfoxonium triflates **5** **a**–**d** as aryl sources and olefins **9** **a**–**c** as coupling partners. Details of the reaction optimization are presented in the Supporting Information (Table S1). In this case, a combination of Pd(OAc)_2_, 1,10‐phenanthroline (1,10‐phen), and Cs_2_CO_3_ proved optimal. As solvent, DCE was best, and the couplings were performed at 100 °C for 18 h under argon. Scheme 3 shows the results.

**Scheme 3:**
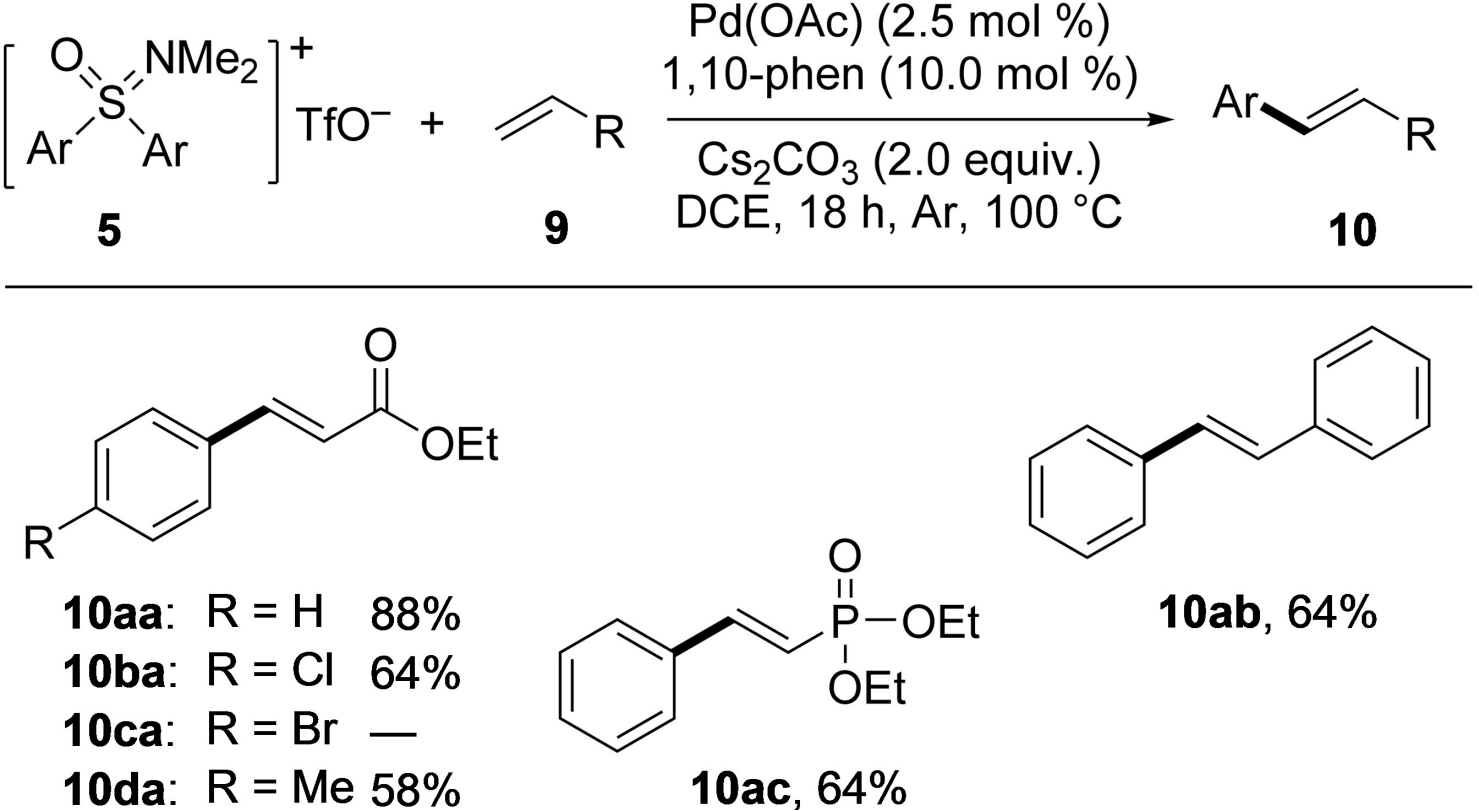
Substrate scope of Heck‐type coupling reactions with oxosulfonium salts **5** and olefins **9**.

In five out of six cases, the couplings proceeded well providing the products in moderate to good yields. Besides the reactions of **5** **a**, **5** **b**, and **5** **d** with ethyl acrylate (**9** **a**) leading to **10** **aa** (88%), **10** **ba** (64%), and **10** **da** (58%), the couplings of **5** **a** with styrene (**9** **b**) and diethyl vinylphosphonate (**9** **c**), which both led to a yield of 64% for products **10** **ab** and **10** **ac**, respectively, are noteworthy. Confirming the results presented above for the Sonogashira‐type coupling, salt **5** **c** proved problematic here too. Instead of **10** **ca**, a complex product mixture was formed, which remained unidentified.

Finally, Suzuki‐type couplings with combinations of five diaryl sulfoxonium triflates **5** and three boronic acids **11** were investigated (Scheme 4). Diphenyl sulfoxonium triflate salt **5** **a** and *p*‐tolylboronic acid (**11** **a**) were applied in the search for the optimal reaction conditions. Details of this study are provided in the Supporting Information (Table S2).

**Scheme 4:**
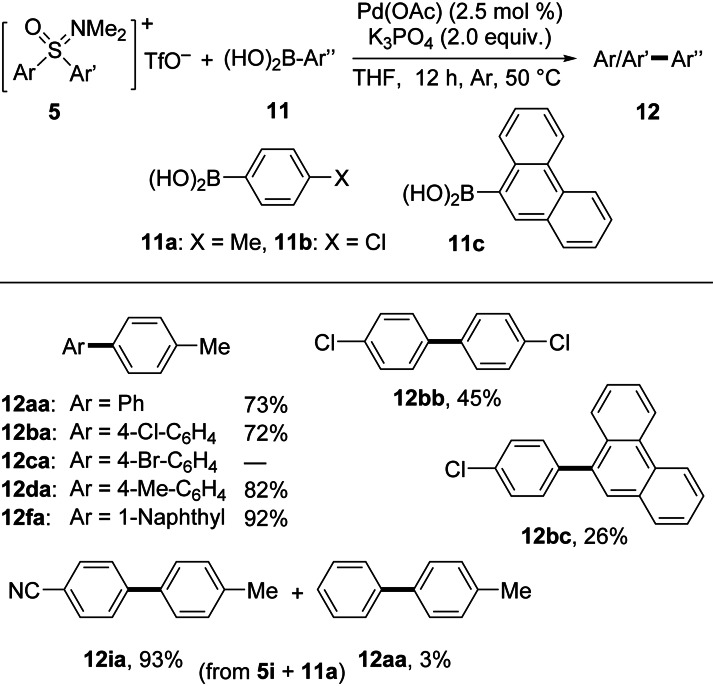
Substrate scope of Suzuki‐type coupling reactions with oxosulfonium salts **5** and aryl boronic acids **11**.

The Suzuki‐type reactions proceeded under similar conditions as the aforementioned cross‐couplings, with the exception that here, no ligand was needed for Pd(OAc)_2_. Furthermore, K_3_PO_4_ was the best base and THF the optimal solvent. In general, the cross‐couplings proceeded well, albeit leading to mixed results with respect to the yields. Thus, while products **12** **aa**, **12** **ba**, **12** **da**, and **12** **fa** were obtained in yields between 72% (for **12** **ba**) and 92% (for **12** **fa**), **12** **bb** and **12** **bc** were only formed in 45% and 26%, respectively. The latter result was attributed to the poor solubility of boronic acid **11** **c**. Also in this case, **5** **c** reacted sluggishly, and **12** **ca** could not be isolated. In this series, we also applied unsymmetrically substituted sulfoxonium triflate salt **5** **i**, and to our delight, we found a very pronounced aryl transfer selectivity in the coupling with boronic acid **11** **b**. The formation of 93% of **12** **ia** and 3% of **12** **aa** revealed a high preference for the cross‐coupling of the aryl group bearing an electron‐withdrawing 4‐cyano substituent over the unsubstituted phenyl. This observation was subsequently supported by results from a competition experiment related to a Sonogashira coupling (Scheme 5).

**Scheme 5:**
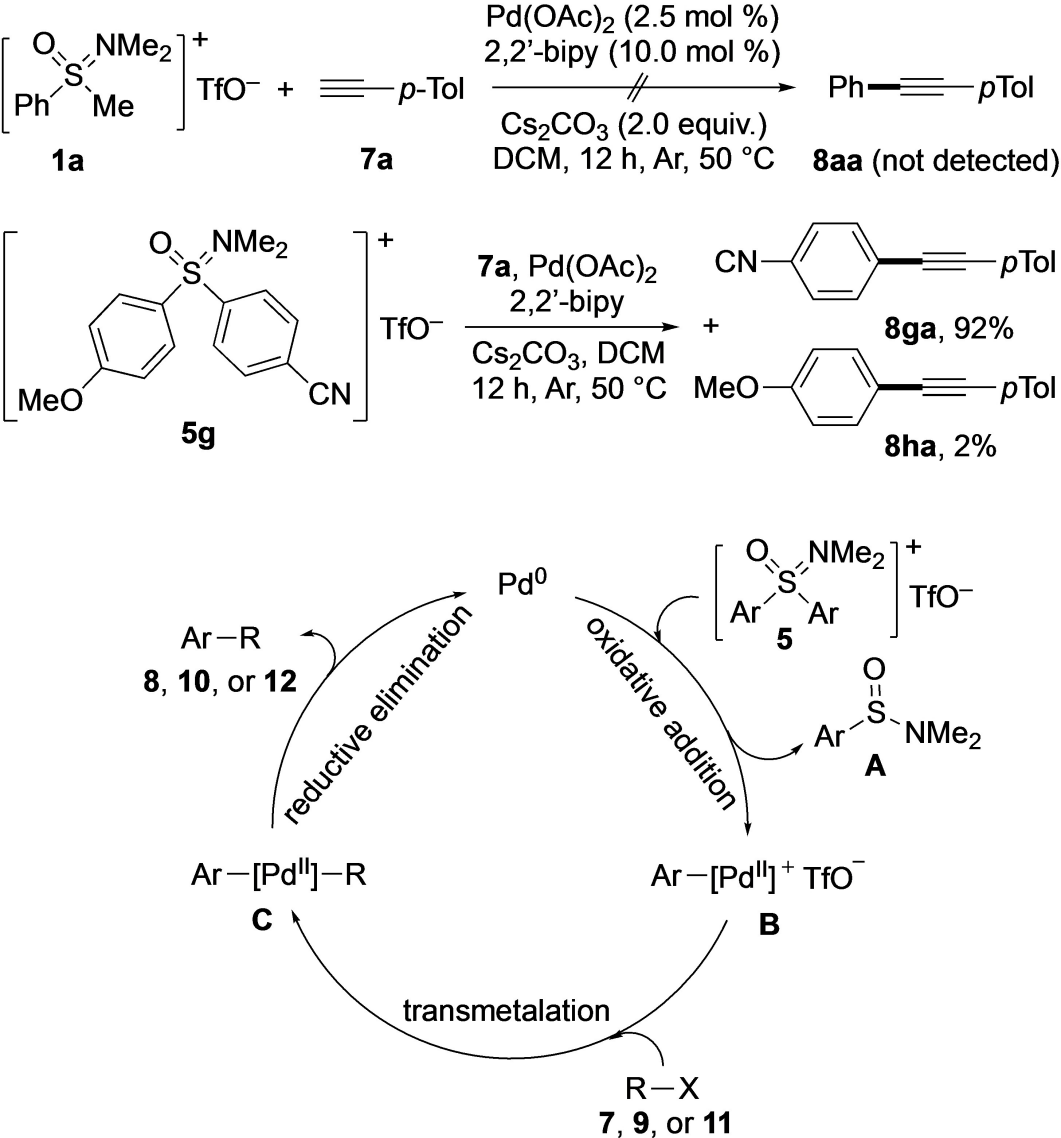
Control experiments and proposed mechanistic pathway.

In order to elucidate the reaction pathway, various process modifications were introduced. The Sonogashira‐type coupling with **7** **a** as reaction partner was selected as study case. First, the amount of **5** **a** was halved from 0.2 mmol to 0.1 mmol. Under the otherwise optimal conditions the yield of **8** **aa** was reduced to 42%, which indicated the importance of the aryl source quantity. Second, the attempt to substitute the *S*,*S*‐diaryl sulfoxonium triflate salts **5** in the Sonogashira‐type coupling with **7** **a** by the *S*‐methyl‐*S*‐phenyl analogue **1** **a** remained unsuccessful (Scheme 5). No product formation occurred, revealing the decisive role of the two *S*‐aryl substituents of **5** for the cross‐coupling process. Third, as discussed before, a ratio of 31 : 1 for products **12** **ia** and **12** **aa** was observed in the Suzuki‐type coupling of unsymmetrically substituted **5** **i** with *p*‐tolyl boronic acid (**11** **b**; Scheme 4). A similar trend was revealed, when **5** **g** was reacted with **7** **a** (Scheme 5). Also in this case, the aryl with the electron‐withdrawing 4‐cyano substituent was transferred with high preference over its 4‐methoxy‐bearing counterpart leading to products **8** **ga** and **8** **ha** in a ratio of 46 : 1.

Taking all observations and precious reports[^13^, ^14^] into account, the following mechanistic scenario can be suggested: The catalytic cycle begins with in‐situ generated palladium(0), which oxidatively adds *S,S*‐diaryl sulfoxonium triflate **5** leading to a palladium(II) intermediate **B** by loss of *N,N*‐dimethylarylsulfinamide **A**. Subsequently, **B** reacts with substrates **7**, **9** or **11** to give palladium(II) intermediate **C**. From **C**, product formation occurs by reductive elimination, which also regenerates the initial palladium(0) species and thereby closes the catalytic cycle.

In summary, we prepared a series of diaryl sulfoxonium triflate salts in good to excellent yields. The substrate scope was broad, and various functional groups, including halo, alkyl, acetoxy, and nitro groups were tolerated. Subsequently, those salts were applied as aryl sources in palladium‐catalyzed cross‐couplings. In all three reaction types – Sonogashira, Heck, and Suzuki – the C−C‐bond formations proceeded well and good product yields were achieved. Competition experiments showed that aryl groups with electron‐withdrawing substituents are preferentially transferred over aryls bearing electron‐donating groups.

## Conflict of interest

The authors declare no conflict of interest.

## Supporting information

As a service to our authors and readers, this journal provides supporting information supplied by the authors. Such materials are peer reviewed and may be re‐organized for online delivery, but are not copy‐edited or typeset. Technical support issues arising from supporting information (other than missing files) should be addressed to the authors.

Supporting Information

## Data Availability

The data that support the findings of this study are available in the supplementary material of this article.
