# Supplementary material for: The Preparation of Diaryl Sulfoxinium Triflates and Their Application in Palladium‐Catalyzed Cross‐Coupling Reactions
Source: Chem Asian J. 2022 Aug 29;17(19):e202200828. doi: 10.1002/asia.202200828 (PMC9804342; doi:10.1002/asia.202200828)

CHEMISTRY
AN ASIAN JOURNAL

Supporting Information

The Preparation of Diaryl Sulfoxinium Triflates and Their Application in Palladium-Catalyzed Cross-Coupling Reactions

Chenyang Wang[+], Xianliang Wang[+], Deshen Kong, Khai-Nghi Truong, Kari Rissanen, and Carsten Bolm*© 2022 The Authors. Chemistry - An Asian Journal published by Wiley-VCH GmbH. This is an open access article under the terms of the Creative Commons Attribution License, which permits use, distribution and reproduction in any medium, provided the original work is properly cited.

Table of Contents

1. General information S2

2. Preparation of the diaryl sulfoxonium triflate salts S2

3. Applications in Pd-catalyzed cross-coupling reactions S3

4. Characterizing data S5

5. X-ray crystallography S12

6. References S13

7. NMR spectra S15

1. General information

All reagents were obtained commercially unless otherwise noted, and solvents were taken from commercial solvent purification system (SPS). The reactions were monitored by thin layer chromatography (TLC) with aluminum sheets silica gel 60 F_{254} from Merck, and flash column chromatography purifications were performed using silica gel 60 (63-200 µm) from Merck. Nuclear Magnetic Resonance (NMR) spectra were acquired on an Agilent VNMRS 600, Agilent VNMRS 400, or Varian Mercury 300. Chemical shifts were reported in δ ppm (parts per million) and *J* (spin-spin coupling constants) were showed in Hz. ^{1}H and ^{13}C {^{1}H} NMR spectra were processed and analyzed with the program MestReNova. Chemical shifts (d) are given in parts per million (ppm) and spin-spin coupling constants (*J*) are reported in Hertz (Hz). Proton and carbon NMR spectra were referenced to the solvent residue signal of the non-deuterated solvent ($CHCl_3$: ^{1}H NMR: δ = 7.26 ppm, $CDCl_3$: ^{13}C{^{1}H} NMR: δ = 77.0 ppm; $(CH_3)_2SO$: ^{1}H NMR: δ = 2.50 ppm, $(CD_3)_2SO$: ^{13}C{^{1}H} NMR: δ = 39.52 ppm. Carbon spectra were measured by proton broadband decoupling, are abbreviated by s (singlet), d (doublet), t (triplet), q (quartet), br (broad), m (multiplet). Mass spectra were measured on Finnigan SSQ Finnigan 7000 spectrometer (EI, 70 eV). High-resolution mass spectra (HRMS) were measured on Thermo Scientific LTQ Orbitrap XL spectrometer. All IR data were collected by attenuated total reflectance (ATR) and wavenumbers *v* are given in cm^{-1}. Melting points (mp) was measured on Bϋchi B-540 melting point apparatus.

2. Preparation of diaryl sulfoxonium triflate salts

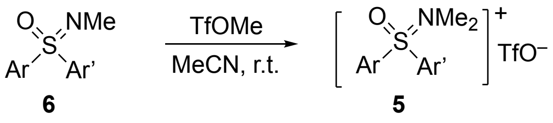

In a 25 mL ground flask, *N*-methyl sulfoximimine **6**[S1] (5 mmol) was dissolved in acetonitrile (2 mL). After the dropwise addition of MeOTf (5.5 mmol, 1.1 equiv) over 5 min, the mixture was stirred for 2 h at ambient temperature.[S2] When the reaction was complete, the solvent was removed under vacuum. Then, the solid crude product was washed with ethyl acetate (3 x 3 mL) to provide the pure product. [If no solid crude product was formed after removing the solvent, the product was purified by a short flash column chromatography (ethyl acetate: methanol = 15:1)].

3. Applications in Pd-catalyzed cross-coupling reactions

3.1 Sonogashira reaction[S3]

3.1.1 General procedure for the Sonogashira reaction

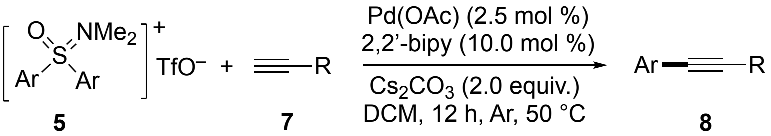

5 **7** **8**

To a 25 mL Schlenk tube with a stirring bar, diaryl sulfoxonium triflate salt **5** (0.2 mmol), $Pd(OAc)_2$ (1.1 mg, 0.005 mmol, 2.5 mol %), 2,2'-bipyridine (3 mg, 0.02 mmol, 10 mol %), and Cs_2CO_3 (130 mg, 0.4 mmol, 2.0 equiv) are added in a glovebox. The solid mixture is dissolved in DCM (1.5 mL) and then, arylacetylene **7** (0.4 mmol, 2.0 equiv) is added by syringe. After stirring at 50 °C for 12 h, the solvent is removed under vacuum and product **8** is purified by column chromatography with pentane as the eluant.

3.2 Heck reaction[S4]

3.2.1 Optimization of the reaction conditions

Table S1 Optimization of the Heck reaction with diaryl sulfoxonium triflate **5** as arylating agent.[a]

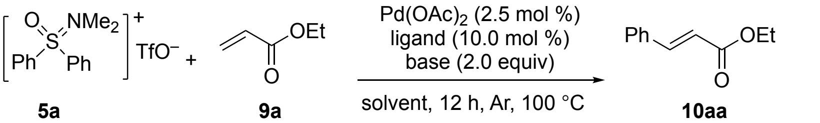

5a **9a** **10aa**

Entry	Solvent	Base	Ligand	Yield [%][b]
1	DMF	Et_3N	PPh_3	15
2	toluene	Et_3N	PPh_3	trace
3	DCE	Et_3N	PPh_3	35
4	DCE	K_2CO_3	PPh_3	42
5	DCE	Cs_2CO_3	PPh_3	54
6	DCE	K_3PO_4	PPh_3	22
7	DCE	*t*BuOK	PPh_3	15
8	DCE	NaOH	PPh_3	45
9	DCE	Cs_2CO_3	/	5
10	DCE	Cs_2CO_3	Xphos	13
11	DCE	Cs_2CO_3	2,2'-bipyridyl	54
12	DCE	Cs_2CO_3	2,2'-bipyridyl (60 °C)	44
13	DCE	Cs_2CO_3	1,10-phenanthroline	77
14	DCE	Cs_2CO_3	1,10-phenanthroline (18 h)	89 (88)
15	DCE	Cs_2CO_3	4,4'-dimethoxy-2,2'-bipyridin	14
16	DCE	Cs_2CO_3	dtbbpy	72
17				
18				
19				

[a]Use of 0.2 mmol of **5a**, 2.0 equiv of **9a**, 2.0 equiv of the base in 1.5 mL of the solvent.
[b]Determined by ^1H NMR analysis of the crude reaction mixture using CH_2Br_2 as internal standard. In parentheses, yield of isolated product.

3.2.2 General procedure for the Heck reaction

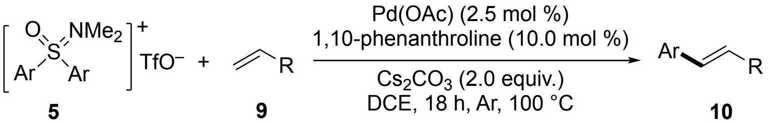

5 **9** **10**

To a 25 mL Schlenk tube with a stirring bar, diaryl sulfoxonium triflate salt **5** (0.2 mmol), $Pd(OAc)_2$ (1.1 mg, 0.005 mmol, 2.5 mol %), 1,10-phenathroline (3.6 mg, 0.02 mmol, 10 mol % and Cs_2CO_3 (130 mg, 0.4 mmol, 2.0 equiv) are added in a glovebox. The solid mixture is dissolved in DCE (1.5 mL), and ethyl acrylate (**9a**), styrene (**9b**) or diethyl vinylphosphonate (**9c,** 0.4 mmol, 2.0 equiv) is added by syringe. After stirring at 100 ºC for 18 h, the solvent is removed under vacuum, and the product is purified by column chromatography (with pentane to pentane/ethyl acetate = 4:1 as eluent) to give product **10**.

3.3 Suzuki coupling reaction[S5]

3.3.1 Optimization of the reaction conditions

Table S2 Optimization of the Suzuki reaction with diaryl sulfoxonium triflate **5** as arylating agent.[a]

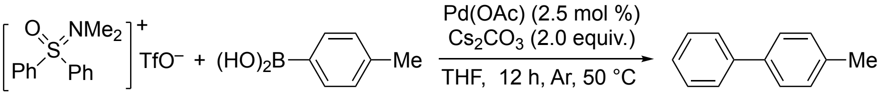

5a **11a** **12aa**

Modification of reaction conditions	Yield [%][b]
None	80
CH_3CN instead of THF	30
1,4-dioxane instead of THF	64
toluene instead of THF	70
DCE instead of THF	75
K_2CO_3 instead of Cs_2CO_3	66
Na_2CO_3 instead of Cs_2CO_3	45
$NaHCO_3$ instead of Cs_2CO_3	65
K_3PO_4 instead of Cs_2CO_3	82 (73)
*t*BuOK instead of Cs_2CO_3	66
Et_3N instead of Cs_2CO_3	50

[a]Use of 0.2 mmol of **5a**, 2.0 equiv of **11a**, 2.0 equiv of the base in 1.5 mL of the solvent. [b]Determined by ^1H NMR analysis of the crude reaction mixture using CH_2Br_2 as the internal standard. In parentheses, yield of isolated product.

3.3.2 General procedure for the Suzuki reaction

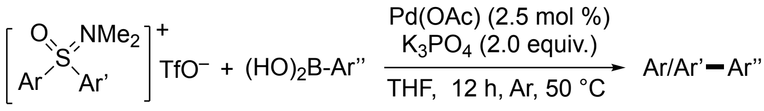

5 **11** **12**

To a 25 mL Schlenk tube with a stirring bar, diaryl sulfoxonium triflate salt **5** (0.2 mmol), aryl boronic acid **11** (0.4 mmol, 2.0 equiv), $Pd(OAc)_2$ (1.1 mg, 0.005 mmol, 2.5 mol %), and K_3PO_4 (85 mg, 0.4 mmol, 2.0 equiv) are added in a glovebox. The solid mixture is dissolved in THF (1.5 mL). After stirring at 50 ºC for 12 h, the solvent is removed under vacuum, and the product is purified by column chromatography (with pentane as eluent) to give product **12**.

4. Characterizing data

***N*-Methyl-*N*-(oxodiphenyl-λ⁶-sulfaneylidene)methanaminium trifluoromethanesulfonate (5a)**

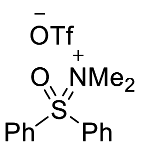

Following the general procedure for the preparation of diaryl sulfoxonium triflate salts, **5a** was isolated in 97% yield (white solid, 1.92 g). **Melting point**: 140-142 ºC. **^{1}H NMR (400 MHz, CDCl₃)** δ 8.43 (d, *J* = 7.2 Hz, 4H), 7.90 – 7.71 (m, 6H), 3.16 (s, 6H). **^{13}C {^{1}H} NMR (151 MHz, CDCl₃)** δ 137.3, 131.6, 129.7, 129.6, 121.0 (q, *J* = 320.5 Hz), 38.6. **^{19}F NMR (376 MHz, CDCl₃)** δ –78.24. **IR (ATR):** 3096, 2944, 1835, 1577, 1452, 1259, 1148, 1028, 944, 774, 736, 687 cm^{-1}. **HRMS (ESI) *m/z* [M-OTf]$^+$** = calcd. 246.0947, found 246.0942. **MS (EI): *m/z*** = 247 (16), 246 (100), 203 (16), 83 (13), 77 (11).

***N*-[Bis(4-chlorophenyl)(oxo)-λ⁶-sulfaneylidene]-*N*-methylmethanaminium trifluoromethanesulfonate (5b)**

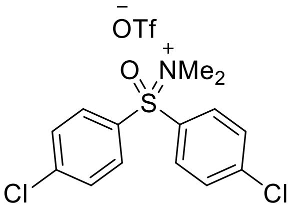

Following the general procedure for the preparation of diaryl sulfoxonium triflate salts, **5b** was isolated in 91% yield (white solid, 2.11 g). **Melting point**: 147-149 ºC. **^{1}H NMR (400 MHz, CDCl₃)** δ 8.39 (d, *J* = 9.0 Hz, 4H), 7.76 (d, *J* = 8.9 Hz, 4H), 3.16 (s, 6H). **^{13}C {^{1}H} NMR (101 MHz, CDCl₃)** δ 145.2, 132.1, 131.2, 127.5, 120.8 (q, *J* = 320.1 Hz), 38.7. **^{19}F NMR (376 MHz, CDCl₃)** δ –78.25. **IR (ATR):** 3369, 3087, 2947, 1623, 1569, 1469, 1398, 1469, 1398, 1258, 1157, 1078, 1028, 956, 833, 760 cm^{-1}. **HRMS (ESI) *m/z* [M-OTf]$^+$** = calcd. 314.0167, found 314.0163. **MS (EI): *m/z*** = 318 13), 317 (11), 316 (68), 315 (17), 314 (100), 274 (16), 271 (14), 254 (12), 222 (13), 159 (15), 143 (16), 75 (13).

***N*-[Bis(4-bromophenyl)(oxo)-λ⁶-sulfaneylidene]-*N*-methylmethanaminium trifluoromethanesulfonate (5c)**

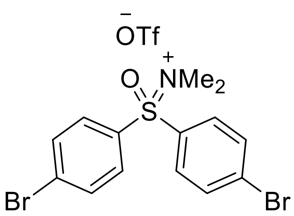

Following the general procedure for the preparation of diaryl sulfoxonium triflate salts, **5c** was isolated in 90% yield 90% (white solid, 2.49 g). **Melting point**: 170-173 ºC **^{1}H NMR (600 MHz, CDCl₃)** δ 8.47 – 8.07 (m, 4H), 8.06 – 7.87 (m, 4H), 3.19 (s, 6H). **^{13}C {^{1}H}**

NMR (151 MHz, CDCl₃) δ 135.2, 134.2, 131.1, 128.2, 120.8 (q, J = 320.1 Hz), 38.8. **^{19}F NMR (565 MHz, CDCl₃)** δ –78.25. **IR (ATR):** 3368, 3086, 2944, 1649, 1564, 1466, 1390, 1257, 1158, 1065, 1028, 954, 751 cm^{-1}. **HRMS (ESI) *m/z* [M-OTf]$^+$** = calcd. 401.9157, found 401.9160. **MS (EI):** ***m/z*** = 406 (36), 404 (68), 402 (34), 344 (15), 186 (27), 184 (34), 85 (65), 83 (100), 49 (11), 48 (21), 47 (36).

***N*-Methyl-*N*-(oxodi-*p*-tolyl-λ^6-sulfaneylidene)methanaminium trifluoromethanesulfonate (5d)**

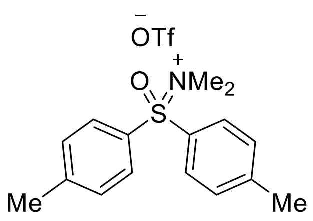

Following the general procedure for the preparation of diaryl sulfoxonium triflate salts, **5d** was isolated in 95% yield (white solid, 2.01 g). **Melting point**: 142-143 ºC. **^{1}H NMR (400 MHz, CDCl₃)** δ 8.19 (d, J = 8.6 Hz, 4H), 7.55 (d, J = 9.2 Hz, 4H), 3.08 (s, 6H), 2.44 (s, 6H). **^{13}C {^{1}H} NMR (101 MHz, CDCl₃)** δ 149.2, 132.1, 129.5, 126.4, 120.9 (q, J = 320.5 Hz), 38.3, 21.8. **^{19}F NMR (376 MHz, CDCl₃)** δ –78.22. **IR (ATR):** 3508, 3093, 2945, 1662, 1589, 1449, 1409, 1256, 1150, 1082, 1027, 956, 806, 734, 680 cm^{-1}. **HRMS (ESI) *m/z* [M-OTf]$^+$** = calcd. 274.1260, found 274.1255. **MS (EI):** ***m/z*** = 275 (19), 274 (100), 231 (11), 214 (9), 182 (9).

(Dimethyl-λ^4-azaneylidene)bis(4-methoxyphenyl)-λ^6-sulfanone trifluoromethanesulfonate (5e)

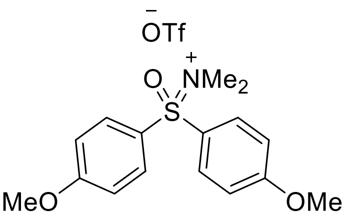

Following the general procedure for the preparation of diaryl sulfoxonium triflate salts, **5e** was isolated in 86% yield (white solid, 1.96 g). **Melting point**: 113-114 ºC. **^{1}H NMR (400 MHz, CDCl₃)** δ 8.30 – 8.22 (m, 4H), 7.24 – 7.17 (m, 4H), 3.90 (s, 6H), 3.08 (s, 6H). **^{13}C {^{1}H} NMR (101 MHz, CDCl₃)** δ 166.4, 131.9, 121.0 (q, J = 320.5 Hz), 119.8, 116.9, 56.3, 38.2. **^{19}F NMR (376 MHz, CDCl₃)** δ –78.25. **IR (ATR):** 3082, 2949, 1701, 1586, 1491, 1462, 1420, 1319, 1256, 1222, 1154, 1084, 1021, 958, 804, 711, 688 cm^{-1}. **HRMS (ESI) *m/z* [M-OTf]$^+$** = calcd. 306.1158, found 306.1158. **MS (EI):** ***m/z*** = 307 (18), 306 (100), 246 (28), 214 (21), 155 (17), 136 (27), 85 (52), 83 (80), 48 (19), 47 (32).

***N*-[Di(naphthalen-1-yl)(oxo)-λ^6-sulfaneylidene]-*N*-methylmethanaminium trifluoromethane-sulfonate (5f)**

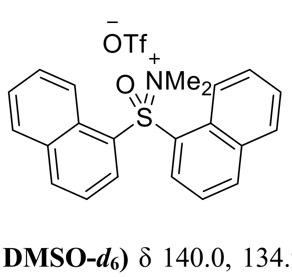

Following the general procedure for the preparation of diaryl sulfoxonium triflate salts, **5f** was isolated in 64% yield (white solid, 1.58 g). **Melting point**: 141-142 ºC ºC. **^{1}H NMR (600 MHz, DMSO-*d*₆)** δ 8.69 (d, J = 8.2 Hz, 2H), 8.40 – 8.33 (m, 4H), 8.24 (d, J = 7.6 Hz, 2H), 7.91 – 7.79 (m, 6H), 3.32 (s, 6H). **^{13}C {^{1}H} NMR (151 MHz, DMSO-*d*₆)** δ 140.0, 134.9, 134.3, 131.2, 130.8, 129.0, 128.5, 125.9, 124.2, 123.5, 121.2 (q, J = 322.4 Hz), 39.1. **^{19}F NMR (565 MHz, DMSO-*d*₆)** δ –77.71. **IR (ATR):** 3352, 3071, 1714, 1620, 1504, 1454, 1354, 1260, 1150, 1026, 974, 949, 808, 768, 721 cm^{-1}. **HRMS (ESI) *m/z* [M-OTf]$^+$** = calcd. 346.1260, found 346.1254. **MS (EI):** ***m/z*** = 426 (22), 425 (100), 346 (38), 292 (47), 286 (36), 252 (19), 175 (18), 145 (30), 128 (22), 127 (22), 122 (25), 115 (22).

N-[(4-Cyanophenyl)(4-methoxyphenyl)(oxo)-λ^6-sulfaneylidene]-N-methylmethanaminium trifluoromethanesulfonate (5g)

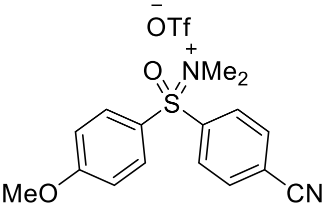

Following the general procedure for the preparation of diaryl sulfoxonium triflate salts, **5g** was isolated in 50% yield (white solid, 1.12 g). **Melting point**: 112-113 °C. **^{1}H NMR (400 MHz, $CDCl_3$)** δ 8.58 (d, J = 8.7 Hz, 2H), 8.31 (d, J = 9.2 Hz, 2H), 8.05 (d, J = 8.7 Hz, 2H), 7.24 (d, J = 9.1 Hz, 2H), 3.91 (s, 3H), 3.14 (s, 6H). **^{13}C {^{1}H} NMR (101 MHz, $CDCl_3$)** δ 167.3, 135.5, 134.9, 132.6, 130.3, 120.81 (q, J = 319.9 Hz), 120.3, 117.4, 116.9, 116.1, 56.5, 38.6. **^{19}F NMR (376 MHz, $CDCl_3$)** δ –78.24. **IR (ATR):** 3091, 2948, 1588, 1492, 1421, 1330, 1261, 1152, 1082, 1023, 957, 829, 735, 676 cm^{-1}. **HRMS (ESI) *m/z* [M-OTf]$^+$** = calcd. 301.1005, found 301.1004. **MS (EI): *m/z*** = 301 (27), 286 (10), 209 (26), 155 (55), 136 (24), 85 (65), 83 (100), 48 (20), 47 (35).

N-Methyl-N-[(4-nitrophenyl)(oxo)(phenyl)-λ^6-sulfaneylidene]methanaminium trifluoromethanesulfonate (5h)

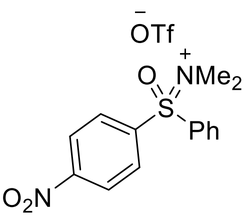

Following the general procedure for the preparation of diaryl sulfoxonium triflate salts, **5h** was isolated in 83% yield (light yellow solid, 1.83 g). **Melting point**: 100-103 °C. **^{1}H NMR (600 MHz, $CDCl_3$)** δ 8.76 (d, J = 9.1 Hz, 2H), 8.59 (d, J = 9.0 Hz, 2H), 8.49 – 8.39 (m, 2H), 7.93 (t, J = 7.5 Hz, 1H), 7.85 (t, J = 8.0 Hz, 2H), 3.22 (s, 6H). **^{13}C {^{1}H} NMR (151 MHz, $CDCl_3$)** δ 152.6, 138.1, 135.9, 132.0, 131.8, 130.1, 128.1, 126.5, 120.8 (q, J = 320.3 Hz), 39.0. **^{19}F NMR (565 MHz, $CDCl_3$)** δ –78.25. **IR (ATR):** 3490, 3106, 1607, 1532, 1452, 1358, 1257, 1148, 1081, 1029, 945, 859, 739 cm^{-1}. **HRMS (ESI) *m/z* [M-OTf]$^+$** = calcd. 291.0797, found 291.0799. **MS (EI): *m/z*** = 291 (43), 247 (10), 125 (18), 106 (10), 87 (10), 85 (65), 83 (100), 77 (13), 49 (10), 48 (19), 47 (33).

N-[(4-Cyanophenyl)(oxo)(phenyl)-λ^6-sulfaneylidene]-N-methylmethanaminium trifluoromethanesulfonate (5i)

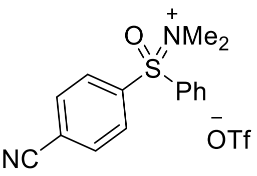

Following the general procedure for the preparation of diaryl sulfoxonium triflate salts, **5i** was isolated in 74% yield (white solid, 1.55 g). **Melting point**: 118-120 °C. **^{1}H NMR (600 MHz, $CDCl_3$)** δ 8.68 (d, J = 8.7 Hz, 2H), 8.46 (d, J = 8.5 Hz, 2H), 8.11 (d, J = 8.8 Hz, 2H), 7.94 (t, J = 7.5 Hz, 1H), 7.86 (t, J = 7.9 Hz, 2H), 3.22 (s, 6H). **^{13}C {^{1}H} NMR (151 MHz, $CDCl_3$)** δ 138.1, 135.1, 134.4, 132.0, 130.7, 130.0, 128.2, 120.9, 120.7 (q, J = 319.3 Hz), 116.0, 38.9. **^{19}F NMR (565 MHz, $CDCl_3$)** δ –78.26. **IR (ATR):** 3427, 3040, 2955, 1703, 1579, 1450, 1402, 1259, 1226, 1149, 1080, 1027, 945, 845, 750, 705, 680 cm^{-1}. **HRMS (ESI) *m/z* [M-OTf]$^+$** = calcd. 271.0899, found 271.0899. **MS (EI): *m/z*** = 271 (28), 87 (10), 85 (64), 83 (100), 69 (12), 48 (15), 47 (29).

N-[Mesityl(oxo)(phenyl)-λ^6-sulfaneylidene]-N-methylmethanaminium trifluoromethanesulfonate (5j)

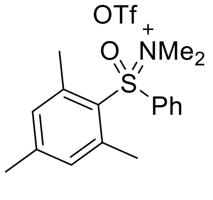

Following the general procedure for the preparation of diaryl sulfoxonium triflate salts, **5j** was isolated in 72% yield (white solid, 1.57 g). **Melting point**: 90-92 °C. **^{1}H NMR (600 MHz, $CDCl_3$)** δ 8.16 (d, J = 8.0 Hz, 2H), 7.93 – 7.87

(m, 1H), 7.86 – 7.80 (m, 2H), 7.18 (s, 2H), 3.25 (s, 6H), 2.53 (s, 6H), 2.41 (s, 3H). **^{13}C {^{1}H} NMR (151 MHz, CDCl$_3$)** δ 148.5, 142.2, 137.0, 134.5, 132.6, 131.1, 129.9, 123.5, 120.9 (q, *J* = 320.7 Hz), 38.6, 23.1, 21.3. **^{19}F NMR (565 MHz, CDCl$_3$)** δ –78.21. **IR (ATR):** 3092, 2998, 1596, 1450, 1388, 1258, 1146, 1084, 1027, 939, 860, 745, 710 cm^{-1}. **HRMS (ESI) *m/z* [M-OTf]$^+$** = calcd. 288.1416, found 288.1417. **MS (EI):** ***m/z*** = 288 (44), 245 (10), 242 (19), 228 (22), 225 (33), 166 (18), 165 (17), 105 (16), 98 (17), 91 (15), 85 (76), 83 (100), 77 (16).

_N_-Methyl-_N_-[naphthalen-2-yl(oxo)(phenyl)-λ^6-sulfaneylidene]methanaminium trifluoromethanesulfonate (5k)

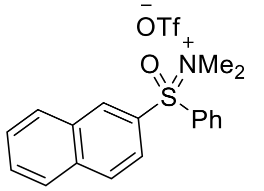

Following the general procedure for the preparation of diaryl sulfoxonium triflate salts, **5k** was isolated in 78% yield (white solid, 1.73 g). **Melting point**: 130-132 °C. **^{1}H NMR (600 MHz, CDCl$_3$)** δ 8.72 (d, *J* = 9.1 Hz, 1H), 8.61 (d, *J* = 7.8 Hz, 1H), 8.36 (d, *J* = 8.1 Hz, 1H), 8.32 (d, *J* = 8.6 Hz, 2H), 8.05 (d, *J* = 8.2 Hz, 1H), 7.96 (t, *J* = 7.8 Hz, 1H), 7.84 (t, *J* = 7.9 Hz, 2H), 7.79 (t, *J* = 7.8 Hz, 2H), 7.72 (t, *J* = 7.6 Hz, 1H), 3.28 (s, 6H). **^{13}C {^{1}H} NMR (151 MHz, CDCl$_3$)** δ 139.6, 137.3, 134.6, 133.9, 131.6, 131.4, 130.3, 129.8, 129.7, 128.7, 128.0, 125.5, 124.0, 123.0, 121.0 (q, *J* = 320.5 Hz), 39.0. **^{19}F NMR (565 MHz, CDCl$_3$)** δ –78.13. **IR (ATR):** 3069, 2957, 1591, 1503, 1354, 1262, 1146, 1078, 1029, 955, 832, 762, 701 cm^{-1}. **HRMS (ESI) *m/z* [M-OTf]$^+$** = calcd. 296.1103, found 296.1104. **MS (EI):** ***m/z*** = 297 (20), 296 (100), 236 (11), 143 (18), 127 (10), 115 (15).

_N_-Methyl-_N_-[5-oxido-5λ^4-dibenzo[_b,d_]thiophen-5-ylidene]methanaminium trifluoromethanesulfonate (5l)

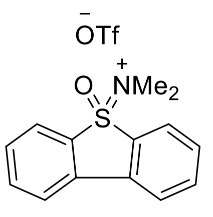

Following the general procedure for the preparation of diaryl sulfoxonium triflate salts, **5l** was isolated in 78% yield (white solid, 1.53 g). **Melting point**: 135-137 °C. **^{1}H NMR (600 MHz, DMSO-*d*$_6$)** δ 8.46 – 8.38 (m, 4H), 8.19 – 8.06 (m, 2H), 7.92 – 7.85 (m, 2H), 3.01 (s, 6H). **^{13}C {^{1}H} NMR (151 MHz, DMSO-*d*$_6$)** δ 140.1, 135.5, 133.1, 127.5, 126.1, 125.3, 121.2 (q, *J* = 322.4 Hz), 37.3. **^{19}F NMR (565 MHz, DMSO-*d*$_6$)** δ –77.73. **IR (ATR):** 3060, 2927, 1582, 1449, 1256, 1165, 1060, 1026, 931, 768, 728 cm^{-1}. **HRMS (ESI) *m/z* [M-OTf]$^+$** = calcd. 244.0790, found 244.0791. **MS (EI):** ***m/z*** = 201 (15), 200 (100), 184 (24), 182 (12), 181 (38), 180 (24), 172 (50), 171 (77), 168 (12).

1-Methyl-4-(phenylethynyl)benzene (8aa)[S6]

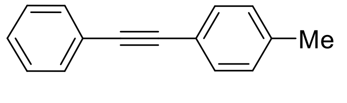

Following the general procedure for the Sonogashira reaction, **8aa** was isolated in 94% yield (white solid, 36 mg). **^{1}H NMR (400 MHz, CDCl$_3$)** δ 7.48 – 7.41 (m, 2H), 7.38 – 7.32 (m, 2H), 7.30 – 7.21 (m, 3H), 7.08 (d, *J* = 7.8 Hz, 2H), 2.29 (s, 3H). **^{13}C {^{1}H} NMR (101 MHz, CDCl$_3$)** δ 137.3, 130.5, 130.5, 128.1, 127.3, 127.0, 122.5, 119.2, 88.5, 87.7, 20.5.

1-Chloro-4-(_p_-tolylethynyl)benzene (8ba)[S6]

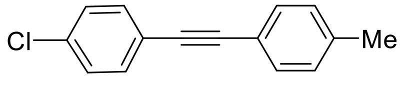

Following the general procedure for the Sonogashira reaction, **8ba** was isolated in 82% yield (white solid, 37 mg). **^{1}H NMR (400 MHz, CDCl$_3$)** δ 7.51 – 7.40 (m, 4H), 7.37 – 7.31 (m, 2H), 7.18 (d, *J* = 7.9 Hz, 2H), 2.40 (s, 3H). **^{13}C {^{1}H} NMR (101 MHz, CDCl$_3$)** δ 138.7, 134.0,

132.7, 131.5, 129.2, 128.7, 122.0, 119.8, 90.5, 87.6, 21.5.

1,2-Di-*p*-tolylethyne (8da)[S6]

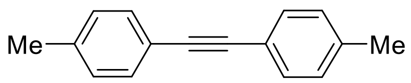

Following the general procedure for the Sonogashira reaction, **8da** was isolated in 85% yield (white solid, 35 mg). **^{1}H NMR (400 MHz, CDCl$_3$)** δ 7.40 (d, *J* = 8.2 Hz, 4H), 7.13 (d, *J* = 7.8 Hz, 4H), 2.35 (s, 6H). **^{13}C {^{1}H} NMR (101 MHz, CDCl$_3$)** δ 138.1, 131.4, 129.0, 120.4, 88.8, 21.5.

1-(*p*-Tolylethynyl)naphthalene (8fa)[S7]

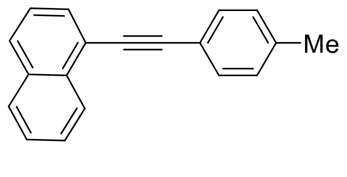

Following the general procedure for the Sonogashira reaction, **8fa** was isolated in 88% yield (white solid, 42 mg). **^{1}H NMR (600 MHz, CDCl$_3$)** δ 8.45 (d, *J* = 8.3 Hz, 1H), 7.87 (d, *J* = 8.1 Hz, 1H), 7.84 (d, *J* = 8.3 Hz, 1H), 7.76 (d, *J* = 7.1 Hz, 1H), 7.63 – 7.57 (m, 1H), 7.57 – 7.51 (m, 3H), 7.46 (t, *J* = 7.7 Hz, 1H), 7.21 (d, *J* = 7.8 Hz, 2H), 2.41 (s, 3H). **^{13}C {^{1}H} NMR (151 MHz, CDCl$_3$)** δ 138.5, 133.3, 133.2, 131.5, 130.2, 129.2, 128.5, 128.3, 126.7, 126.4, 126.3, 125.3, 121.1, 120.3, 94.5, 86.9, 21.6.

1-Chloro-4-[(4-methoxyphenyl)ethynyl]benzene (8bb)[S6]

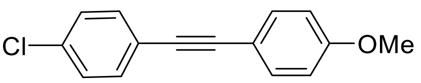

Following the general procedure for the Sonogashira reaction, **8bb** was isolated in 85% yield (white solid, 41 mg). **^{1}H NMR (600 MHz, CDCl$_3$)** δ 7.51 – 7.41 (m, 4H), 7.32 (d, *J* = 8.5 Hz, 2H), 6.89 (d, *J* = 8.8 Hz, 2H), 3.84 (s, 3H). **^{13}C {^{1}H} NMR (151 MHz, CDCl$_3$)** δ 159.8, 133.8, 133.1, 132.6, 128.6, 122.1, 115.0, 114.0, 90.4, 87.0, 55.3.

1-Chloro-4-[(4-fluorophenyl)ethynyl]benzene (8bc)[S8]

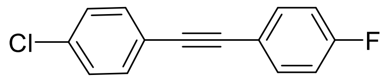

Following the general procedure for the Sonogashira reaction, **8bc** was isolated in 87% yield (yellow solid, 40 mg). **^{1}H NMR (600 MHz, CDCl$_3$)** δ 7.54 – 7.49 (m, 2H), 7.47 – 7.42 (m, 2H), 7.36 – 7.31 (m, 2H), 7.09 – 7.01 (m, 2H). **^{13}C {^{1}H} NMR (151 MHz, CDCl$_3$)** δ 162.6 (d, *J* = 249.9 Hz), 134.3, 133.5 (d, *J* = 8.3 Hz), 132.7, 128.7, 121.6, 119.0, 115.7 (d, *J* = 22.1 Hz), 89.2, 87.9. **^{19}F NMR (564 MHz, CDCl$_3$)** δ –110.55.

1-[(4-Chlorophenyl)ethynyl]-3-fluorobenzene (8bd)[S9]

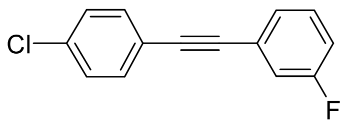

Following the general procedure for the Sonogashira reaction, **8bd** was isolated in 65% yield (colorless oil, 30 mg). **^{1}H NMR (600 MHz, CDCl$_3$)** δ 7.52 – 7.44 (m, 2H), 7.38 – 7.29 (m, 4H), 7.25 – 7.21 (m, 1H), 7.10 – 7.02 (m, 1H). **^{13}C {^{1}H} NMR (151 MHz, CDCl$_3$)** δ 162.4 (d, *J* = 246.7 Hz), 134.6, 132.9, 130.0 (d, *J* = 8.8 Hz), 128.8, 127.5 (d, *J* = 3.0 Hz), 124.8 (d, *J* = 9.4 Hz), 121.3, 118.4 (d, *J* = 22.8 Hz), 115.8 (d, *J* = 21.4 Hz), 89.1, 89.0 (d, *J* = 3.2 Hz). **^{19}F NMR (564 MHz, CDCl$_3$)** δ –112.83.

1-[(4-Chlorophenyl)ethynyl]-2-fluorobenzene (8be)[S10]

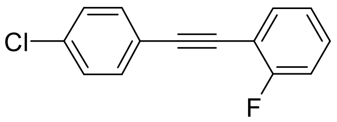

Following the general procedure for the Sonogashira reaction, **8be** was isolated in 67% yield (white solid, 31 mg). **^{1}H NMR (600 MHz, $CDCl_3$)** δ 7.56 – 7.45 (m, 3H), 7.38 – 7.28 (m, 3H), 7.18 – 7.03 (m, 2H). **^{13}C {^{1}H} NMR (151 MHz, $CDCl_3$)** δ 162.6 (d, J = 251.8 Hz), 134.6, 133.4, 132.9, 130.2 (d, J = 8.0 Hz), 128.7, 124.0 (d, J = 3.9 Hz), 121.4, 115.6 (d, J = 20.8 Hz), 111.6 (d, J = 15.9 Hz), 93.2 (d, J = 3.1 Hz), 83.6. **^{19}F NMR (564 MHz, $CDCl_3$)** δ –109.73.

1-Chloro-4-(hex-1-yn-1-yl)benzene (8bf)[S6]

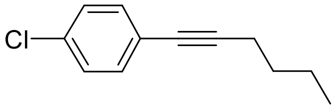

Following the general procedure for the Sonogashira reaction, **8bf** was isolated in 96% yield (white solid, 37 mg). **^{1}H NMR (600 MHz, $CDCl_3$)** δ 7.35 – 7.28 (m, 2H), 7.27 – 7.22 (m, 2H), 2.40 (t, J = 7.1 Hz, 2H), 1.65 – 1.53 (m, 2H), 1.51 – 1.37 (m, 2H), 0.95 (t, J = 7.3 Hz, 3H). **^{13}C {^{1}H} NMR (151 MHz, $CDCl_3$)** δ 133.3, 132.7, 128.4, 122.6, 91.5, 79.5, 30.7, 22.0, 19.1, 13.6.

4-(*p*-Tolylethynyl)benzonitrile (8ga)[S11]

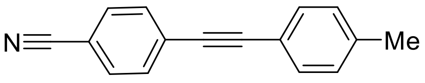

Following the general procedure for the Sonogashira reaction, **8ga** was isolated in 92% yield (white solid, 40 mg). **^{1}H NMR (600 MHz, $CDCl_3$)** δ 7.65 (d, J = 8.6 Hz, 1H), 7.61 (d, J = 8.6 Hz, 1H), 7.46 (d, J = 8.1 Hz, 1H), 7.21 (d, J = 8.5 Hz, 1H), 2.41 (s, 2H). **^{13}C {^{1}H}** NMR (151 MHz, $CDCl_3$) δ 139.5, 132.0, 132.0, 131.7, 129.3, 128.5, 119.2, 118.6, 111.3, 94.1, 87.2, 21.6.

Ethyl cinnamate (10aa)[S12]

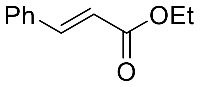

Following the general procedure for the Heck reaction, **10aa** was isolated in 88% yield (light yellow oil, 31 mg). **^{1}H NMR (400 MHz, $CDCl_3$)** δ 7.62 (d, J = 16.0 Hz, 1H), 7.49 – 7.42 (m, 2H), 7.31 (m, 3H), 6.37 (d, J = 16.0 Hz, 1H), 4.20 (q, J = 7.1 Hz, 2H), 1.27 (t, J = 7.1 Hz, 3H). **^{13}C {^{1}H} NMR (101 MHz, $CDCl_3$)** δ 167.0, 144.6, 134.5, 130.2, 128.9, 128.0, 118.3, 60.5, 14.3.

Ethyl (*E*)-3-(4-chlorophenyl)acrylate (10ba)[S12]

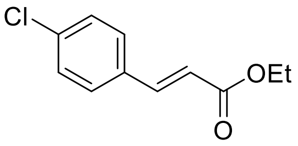

Following the general procedure for the Heck reaction, **10ba** was isolated in 64% yield (white solid, 27 mg). **^{1}H NMR (600 MHz, $CDCl_3$)** δ 7.62 (d, J = 16.1 Hz, 1H), 7.47 – 7.41 (m, 2H), 7.37 – 7.32 (m, 2H), 6.40 (d, J = 16.0 Hz, 1H), 4.26 (q, J = 7.1 Hz, 2H), 1.33 (t, J = 7.1 Hz, 3H). **^{13}C {^{1}H} NMR (151 MHz, $CDCl_3$)** δ 166.7, 143.1, 136.1, 132.9, 129.2, 129.1, 118.8, 60.6, 14.3.

Ethyl (*E*)-3-(*p*-tolyl)acrylate (10da)[S12]

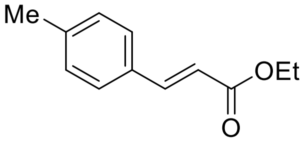

Following the general procedure for the Heck reaction, **10da** was isolated in 58% yield (light yellow oil, 22 mg). **^{1}H NMR (600 MHz, $CDCl_3$)** δ 7.66 (d, J = 16.0 Hz, 1H), 7.42 (d, J = 8.2 Hz, 2H), 7.19 (d, J = 8.3 Hz, 2H), 6.39 (d, J = 16.0 Hz, 1H), 4.26 (q, J = 7.1 Hz, 2H),

2.37 (s, 3H), 1.33 (t, *J* = 7.1 Hz, 3H). **^{13}C {^{1}H} NMR (151 MHz, CDCl$_3$)** δ 167.2, 144.6, 140.6, 131.7, 129.6, 128.0, 117.2, 60.4, 21.4, 14.3.

(*E*)-1,2-Diphenylethene (10ab)[S13]

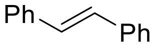

Following the general procedure for the Heck reaction, **10ab** was isolated in 86% yield (white solid, 31 mg). **^{1}H NMR (400 MHz, CDCl$_3$)** δ 7.67 (d, *J* = 8.4 Hz, 4H), 7.51 (t, *J* = 7.5 Hz, 4H), 7.45 – 7.36 (m, 2H), 7.26 (s, 2H). **^{13}C {^{1}H} NMR (101 MHz, CDCl$_3$)** δ 137.4, 128.7, 128.7, 127.6, 126.5.

Diethyl (*E*)-styrylphosphonate (10ac)[S13]

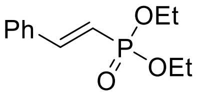

Following the general procedure for the Heck reaction, **10ac** was isolated in 64% yield (colorless oil, 28 mg). **^{1}H NMR (600 MHz, CDCl$_3$)** δ 7.57 – 7.43 (m, 3H), 7.41 – 7.33 (m, 3H), 6.25 (m, 1H), 4.22 – 4.00 (m, 4H), 1.35 (t, *J* = 7.1 Hz, 6H). **^{13}C {^{1}H} NMR (151 MHz, CDCl$_3$)** δ 148.7 (d, *J* = 6.6 Hz), 134.9 (d, *J* = 23.2 Hz), 130.2, 128.9, 127.7, 114.0 (d, *J* = 191.3 Hz), 61.8 (d, *J* = 5.5 Hz), 16.4 (d, *J* = 6.5 Hz).

4-Methyl-1,1'-biphenyl (12aa)[S14]

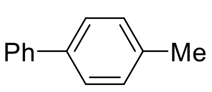

Following the general procedure for the Suzuki coupling reaction, **12aa** was isolated in 73% yield (white solid, 24 mg). **^{1}H NMR (400 Hz, CDCl$_3$)** δ 7.53 – 7.47 (m, 2H), 7.42 (d, *J* = 8.1 Hz, 2H), 7.35 (m, 2H), 7.27 – 7.21 (m, 1H), 7.22 – 7.12 (m, 2H), 2.32 (s, 3H). **^{13}C {^{1}H} NMR (101 Hz, CDCl$_3$)** δ 141.2, 138.4, 137.02, 129.5, 128.7, 127.0, 127.0, 21.1.

4-Chloro-4'-methyl-1,1'-biphenyl (12ba)[S14]

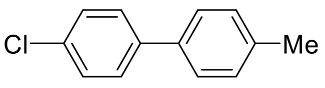

Following the general procedure for the Suzuki coupling reaction, **12ba** was isolated in 72% yield (white solid, 29 mg). **^{1}H NMR (400MHz, CDCl$_3$)** δ 7.53 (d, *J* = 8.3 Hz, 2H), 7.48 (d, *J* = 7.8 Hz, 2H), 7.42 (d, *J* = 8.5 Hz, 2H), 7.28 (d, *J* = 7.9 Hz, 2H), 2.42 (s, 3H). **^{13}C {^{1}H} NMR (101MHz, CDCl$_3$)** δ 139.6, 137.4, 137.1, 133.0, 129.6, 128.9, 128.2, 126.8, 21.1.

4,4'-dimethyl-1,1'-biphenyl (12da)[S15]

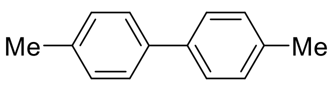

Following the general procedure for the Suzuki coupling reaction, **12da** was isolated in 82% yield (white solid, 30 mg). **^{1}H NMR (400 MHz, CDCl$_3$)** δ 7.51 (d, *J* = 8.1 Hz, 4H), 7.27 (d, *J* = 7.8 Hz, 4H), 2.42 (s, 6H). **^{13}C {^{1}H} NMR (101 MHz, CDCl$_3$)** δ 138.30 136.7, 129.4, 126.8, 21.1.

1-(*p*-Tolyl)naphthalene (12fa)[S16]

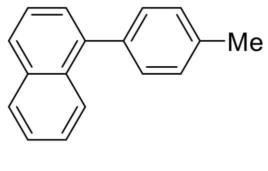

Following the general procedure for the Suzuki coupling reaction, **12fa** was isolated in 92% yield (colorless oil, 40 mg). **^{1}H NMR (600 MHz, CDCl$_3$)** δ 7.98 – 7.88 (m, 2H), 7.87 (d, *J* = 8.3 Hz, 1H), 7.57 – 7.48 (m, 2H), 7.47 – 7.39 (m, 4H), 7.33 (d, *J* = 7.7 Hz, 2H), 2.48 (s, 3H). **^{13}C {^{1}H} NMR (151 MHz, CDCl$_3$)** δ 140.2, 137.8, 136.9, 133.8, 131.7, 130.0, 129.0, 128.2, 127.4, 126.9, 126.1, 125.9, 125.7, 125.4, 21.3.

4,4'-Dichloro-1,1'-biphenyl (12bb)[S14]

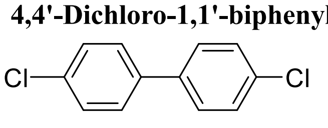

Following the general procedure for the Suzuki coupling reaction, **12bb** was isolated in 45% yield (white solid, 20 mg). **^{1}H NMR (600 MHz, $CDCl_3$)** δ 7.51 – 7.45 (m, 4H), 7.43 – 7.38 (m, 4H). **^{13}C {^{1}H} NMR (151 MHz, $CDCl_3$)** δ 138.4, 133.7, 129.0, 128.2.

9-(4-Chlorophenyl)phenanthrene (12bc)[S17]

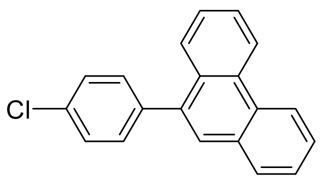

Following the general procedure for the Suzuki coupling reaction, **12bc** was isolated in 26% yield (white solid, 15 mg). **^{1}H NMR (600 MHz, $CDCl_3$)** δ 8.79 (d, *J* = 8.3 Hz, 1H), 8.73 (d, *J* = 8.0 Hz, 1H), 7.90 (dd, *J* = 7.9, 1.4 Hz, 1H), 7.86 (dd, *J* = 8.3, 1.3 Hz, 1H), 7.72 – 7.66 (m, 2H), 7.66 (s, 1H), 7.66 – 7.60 (m, 1H), 7.58 – 7.52 (m, 1H), 7.52 – 7.46 (m, 4H). **^{13}C {^{1}H} NMR (151 MHz, $CDCl_3$)** δ 139.2, 137.5, 133.4, 131.4, 131.4, 130.8, 130.6, 130.0, 128.7, 128.5, 127.6, 126.9, 126.8, 126.6, 126.6, 123.0, 122.5.

4'-Methyl-[1,1'-biphenyl]-4-carbonitrile (12ia)[S15]

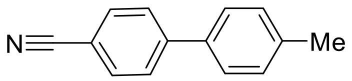

Following the general procedure for the Suzuki coupling reaction, **12ia** was isolated in 93% yield (white solid, 36 mg). **^{1}H NMR (600 MHz, $CDCl_3$)** δ 7.73 – 7.69 (m, 2H), 7.69 – 7.65 (m, 2H), 7.52 – 7.47 (m, 2H), 7.31 – 7.27 (m, 2H), 2.42 (s, 3H). **^{13}C {^{1}H} NMR (151 MHz, $CDCl_3$)** δ 145.6, 138.7, 136.3, 132.6, 129.8, 127.5, 127.0, 119.0, 110.5, 21.2.

5. X-ray crystallography

Single crystals of **5a** were prepared by slow evaporation from DCM/n-pentane/EtOAc at ambient temperature. Then, the crystals of **5a** were analyzed using a dual-source Rigaku SuperNova diffractometer equipped with an Atlas detector and an Oxford Cryostream cooling system using mirror-monochromated Mo-K_α radiation (λ = 0.71073 Å). Data collection and reduction were performed using the program *COLLECT*[S18] and *HKL DENZO AND SCALEPACK*,[S19] respectively, and the intensities were corrected for absorption using *SADABS*.[S20] The structures were solved with Direct Methods (*SHELXS*)[21] and refined by full-matrix least-squares based on F^2 using *SHELXL*-2015.[S9] Non-hydrogen atoms were assigned anisotropic displacement parameters unless stated otherwise. Hydrogen atoms were placed in idealized positions and included as riding. Isotropic displacement parameters for all hydrogen atoms were constrained to multiples of the equivalent displacement parameters of their parent atoms with $U_{iso}(H) = 1.2\ U_{eq}$(parent atom). The X-ray single crystal data, experimental details and CCDC numbers are given below.

Crystal data for **5a**: CCDC-2175966, $C_{15}H_{16}NO_4S_2F_3$, M = 395.41 gmol^{-1}, colorless block, 0.25 × 0.25 × 0.27 mm^3, orthorhombic space group *Pna2₁* (No. 33), a = 19.0279(8) Å, b = 11.3670(4) Å, c = 8.1800(3) Å, V = 1769.3(1) Å^3, Z = 4, D_{calc} = 1.484 gcm^{-3}, F(000) = 816, μ = 0.351 mm^{-1}, T = 120.0(1) K, θ_{max} = 26.24°, 15409 total reflections, 3454 unique, 3246 with $I_o > 2\sigma(I_o)$, R_{int} = 0.0364, 3454 data, 228 parameters, 1 restraints, GooF = 1.065, R_1 = 0.0313 and wR_2 = 0.0702 [$I_o > 2\sigma(I_o)$], R_1 = 0.0347 and wR_2 = 0.0727 (all reflections), Flack x = -0.05(3), $-0.272 < d\Delta\rho < 0.221$ eÅ^{-3}.

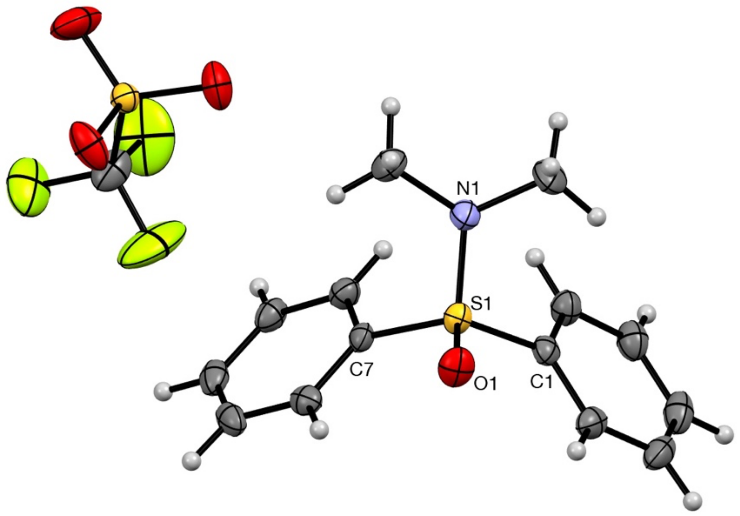

Figure S1. *ORTEP3* plot of **5a**. Displacement ellipsoids are drawn at the 50% probability level.

6. References

[S1] For the synthesis of sulfoximines, see: a) A. Tota, M. Zenzola, S. J. Chawner, S. S. John-Campbell, C. Carlucci, G. Romanazzi, L. Degennaro, J. A. Bull, R. Luisi, Synthesis of *NH*-sulfoximines from sulfides by chemoselective one-pot *N*- and *O*-transfers, *Chem. Commun*. **2017**, *53*, 348-351; for the synthesis of *N*-Me sulfoximines, see: b) L. Rout, P. Saha, S. Jammi, T. Punniyamurthy, Efficient Copper(I)-Catalyzed C–S Cross Coupling of Thiols with Aryl Halides in Water, *Eur. J. Org. Chem.* **2008**, 640-643.

[S2] C. Urban, F. Cadoret, J. C. Blazejewski and E. Magnier, Sulfoximines as a Versatile Scaffold for Electrophilic Fluoroalkylating Reagents, *Eur. J. Org. Chem.* **2011**, 4862-4867.

[S3] K. Sonogashira, Development of Pd–Cu catalyzed cross-coupling of terminal acetylenes with sp^2-carbon halides, *Organomet. Chem.* **2002**, *653*, 46-49.

[S4] R. F. Heck, Palladium-Catalyzed Vinylation of Organic Halides, *Org. React.* **2004**, *27*, 345-390.

[S5] N. Miyaura, K. Yamada, A. Suzuki, A new stereospecific cross-coupling by the palladium-catalyzed reaction of 1-alkenylboranes with 1-alkenyl or 1-alkynyl halides, *Tetrahedron Lett.* **1979**, *20*, 3437-3440.

[S6] D. Zhao, C. Gao, X. Su, Y. He, J. You, Y. Xue, Copper-catalyzed decarboxylative cross-coupling of alkynyl carboxylic acids with aryl halides, *Chem. Commun.,* **2010***, 46,* 9049-9051.

[S7] M. Mastalir, E. Pittenauer, B. Stöger, G. Allmaier, K. Kirchne, Three Different Reactions, One Catalyst: A Cu(I) PNP Pincer Complex as Catalyst for C–C and C–N Cross-Couplings, *Org. Lett.* **2017**, *19*, 2178-2181.

[S8] H. Wang, L. Li, X. Bai, W. Deng, Z. Zheng, K. Yang, L. Xu, One-by-one hydrogenation, cross-coupling reaction, and Knoevenagel condensations catalyzed by $PdCl_2$ and the downstream

palladium residue, *Green Chem.*, **2013**, *15*, 2349-2355.
[S9] A. R. Gholap, K. Venkatesan, R. Pasricha, T. Daniel, R. J. Lahoti, K. V. Srinivasan, Copper- and Ligand-Free Sonogashira Reaction Catalyzed by Pd(0) Nanoparticles at Ambient Conditions under Ultrasound Irradiation, *J. Org. Chem.* **2005**, *70*, 4869-4872.
[S10] D. B. Paixao, D. S. Rampon, H. D. Salles, E. G. O. Soares, F. N. Bilheri, P. H. Schneider, Trithiocarbonate Anion as a Sulfur Source for the Synthesis of 2,5-Disubstituted Thiophenes and 2-Substituted Benzo[*b*]thiophenes, *J. Org. Chem.* **2020**, *85*, 12922-12934.
[S11] L. Yang, Y. Li, Q. Chen, Y. Du, C. Cao, Y. Shi, G. Pang, Sonogashira/hydroarylation sequential reactions: catalyzed by NHC-Pd complexes, *Tetrahedron* **2013**, *69*, 5178-5184.
[S12] Z. Huang, Y. Tang, Unexpected Catalyst for Wittig-Type and Dehalogenation Reactions, *J. Org. Chem.* **2002**, *67*, 5320-5326.
[S13] X. Mi, M. Huang, H. Guo, Y. Wu, An efficient palladium(II) catalyst for oxidative Heck-type reaction under base-free conditions, *Tetrahedron* **2013**, *69*, 5123-5128.
[S14] J. Mao, Q. Hua, G. Xie, J. Guo, Z. Yao, D. Shi, S. Ji, Iodine-Catalyzed Suzuki–Miyaura Coupling Performed in Air, *Adv. Synth. Catal.* **2009**, *351*, 635-641.
[S15] J. Hamdi, A. A. Blanco, B. Diehl, J. B. Wiley, M. L. Trudell, Room-Temperature Aqueous Suzuki-Miyaura Cross-Coupling Reactions Catalyzed via a Recyclable Palladium@Halloysite Nanocomposite, *Org. Lett.* **2019**, *21*, 3471-3475.
[S16] M.-T. Chen, D. A. Vicic, M. L. Turner, O. Navarro, (*N*-Heterocyclic Carbene)$PdCl_2$(TEA) Complexes: Studies on the Effect of the “Throw-Away” Ligand in Catalytic Activity, *Organometallics* **2011**, *30*, 5052-5056.
[S17] R. Jin, Y. Chen, W. Liu, D. Xu, Y. Li, A. Ding, H. Guo, Merging photoredox catalysis with Lewis acid catalysis: activation of carbon–carbon triple bonds, *Chem. Commun.,* **2016**, *52*, 9909-9912.
[S18] R. W. W. Hooft, *COLLECT* **1998**, Nonius BV, Delft, the Netherlands.
[S19] Z. Otwinowski, W. Minor, Processing of X-ray diffraction data collected in oscillation moder. In *Methods in Enzymology*, *Macromolecular Crystallography*, *Part A*, Carter Jr., C. W.; Sweet, R. M., Eds.; Academic Press: New York, **1997**, *276*, pp 307-326.
[S20] G. M. Sheldrick, *SADABS* Version 2008/2 **1996**, University of Göttingen, Germany.
[S21] a) G. M. Sheldrick, A short history of ShelXL. *Acta Cryst.* **2008**, *A64*, 112-122.; b) G. M. Sheldrick, SHELXL13. Program package for crystal structure determination from single crystal diffraction data, University of Göttingen, Germany, 2013; c) G. M. Sheldrick, Crystal structure refinement with *ShelXL*. *Acta Cryst.* **2015**, *C71*, 3-8.

7. NMR spectra of compounds

^{1}H NMR spectrum of compound **5a** (400 MHz, $CDCl_3$)

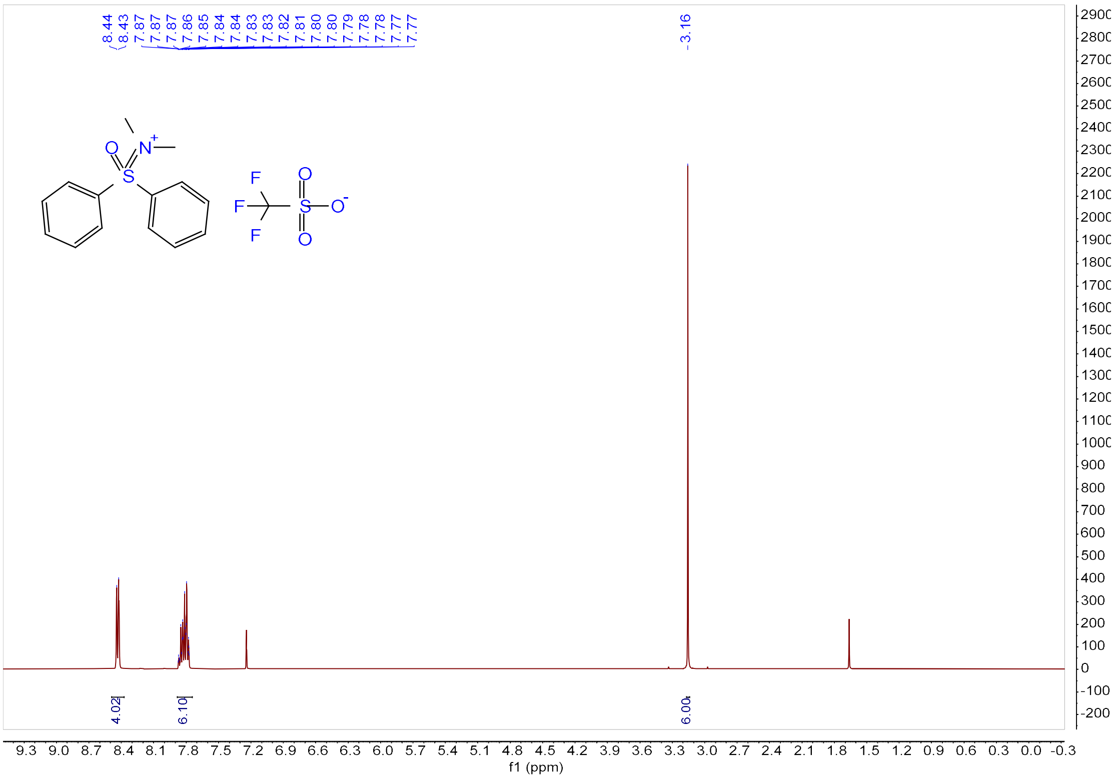

^{13}C NMR spectrum of compound **5a** (151 MHz, $CDCl_3$)

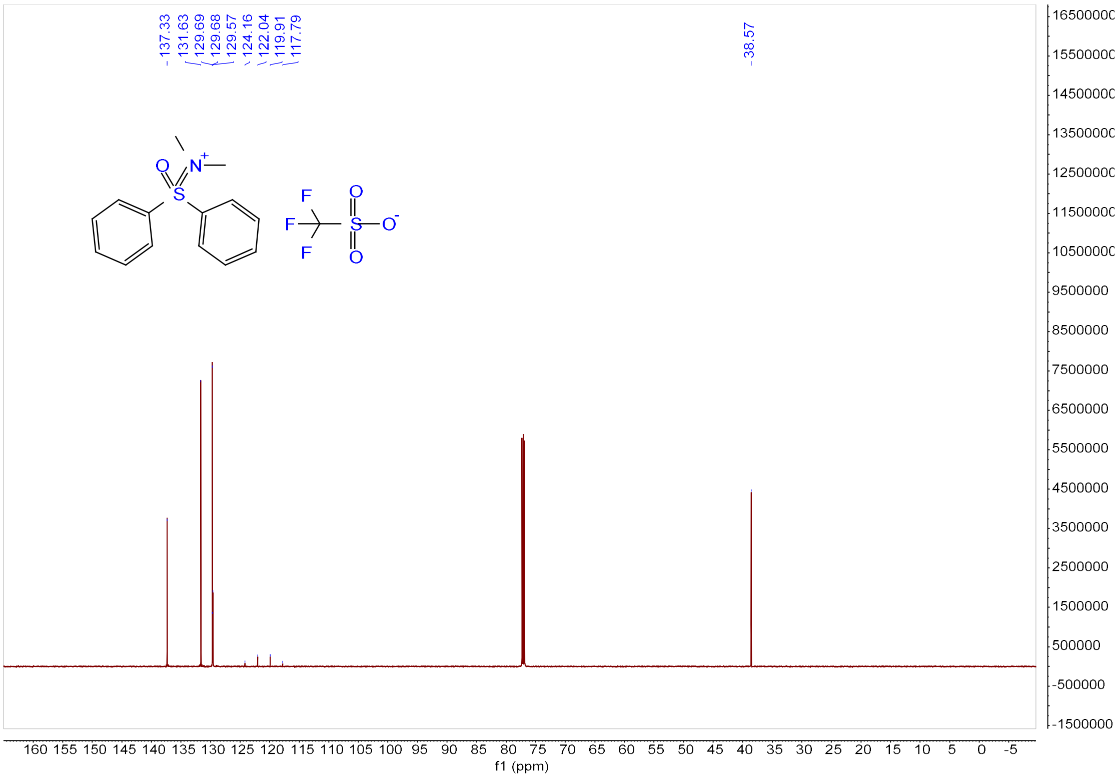

^{19}F NMR spectrum of compound **5a** (376 MHz, CDCl$_3$)

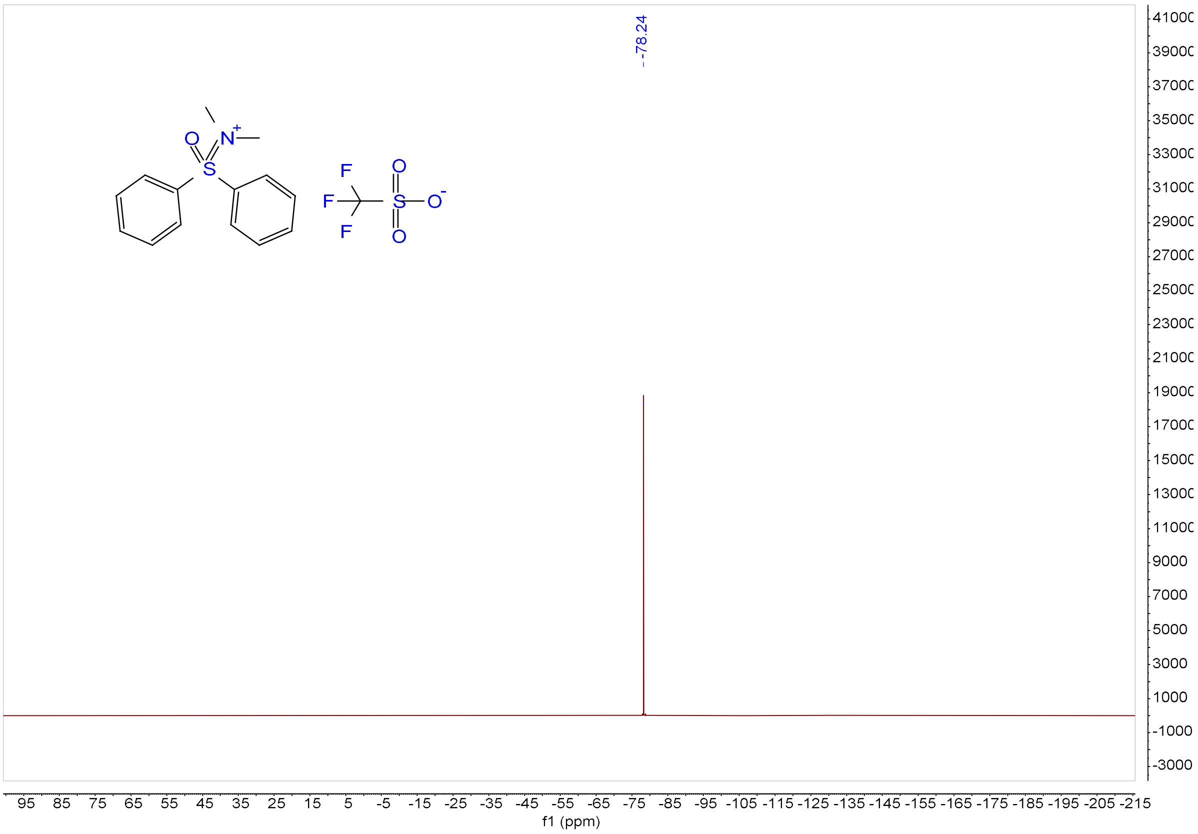

^{1}H NMR spectrum of compound **5b** (400 MHz, CDCl$_3$)

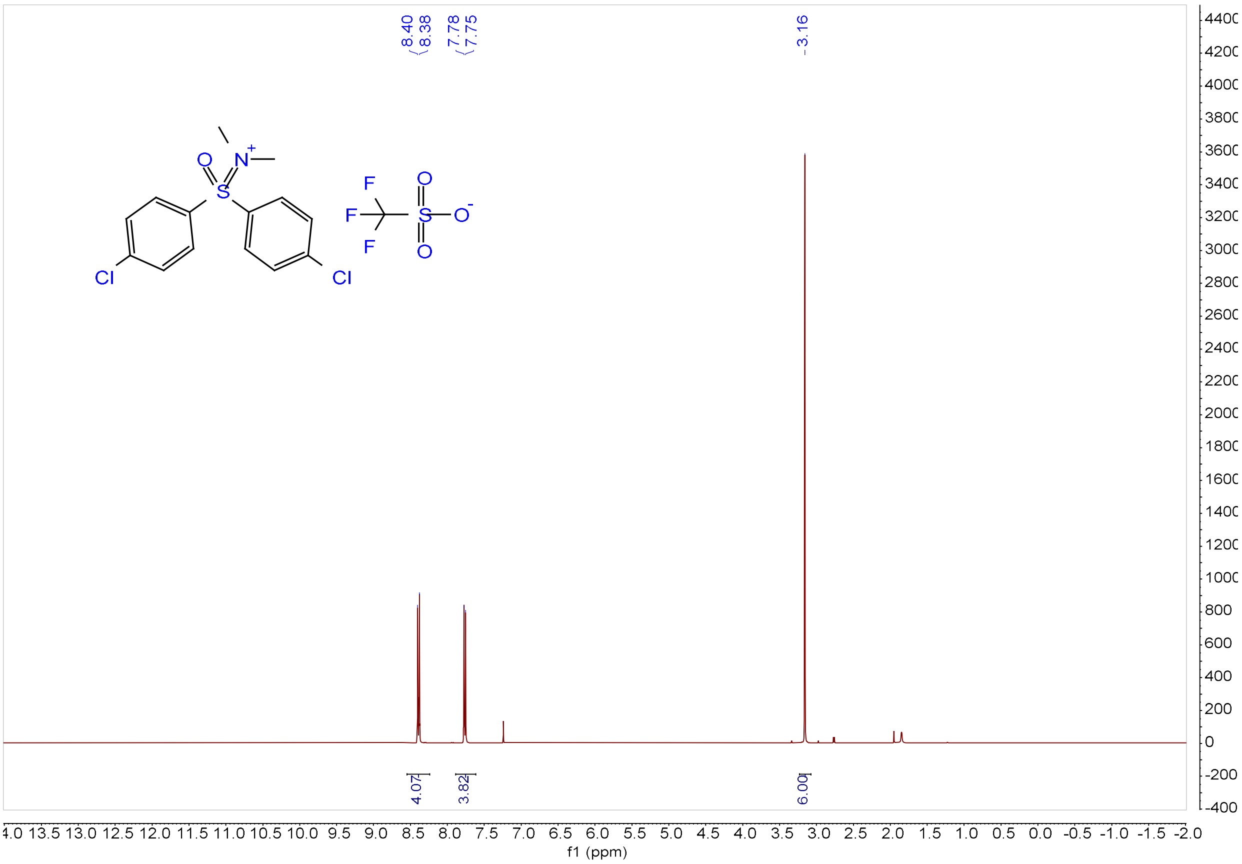

^{13}C NMR spectrum of compound **5b** (101 MHz, CDCl$_3$)

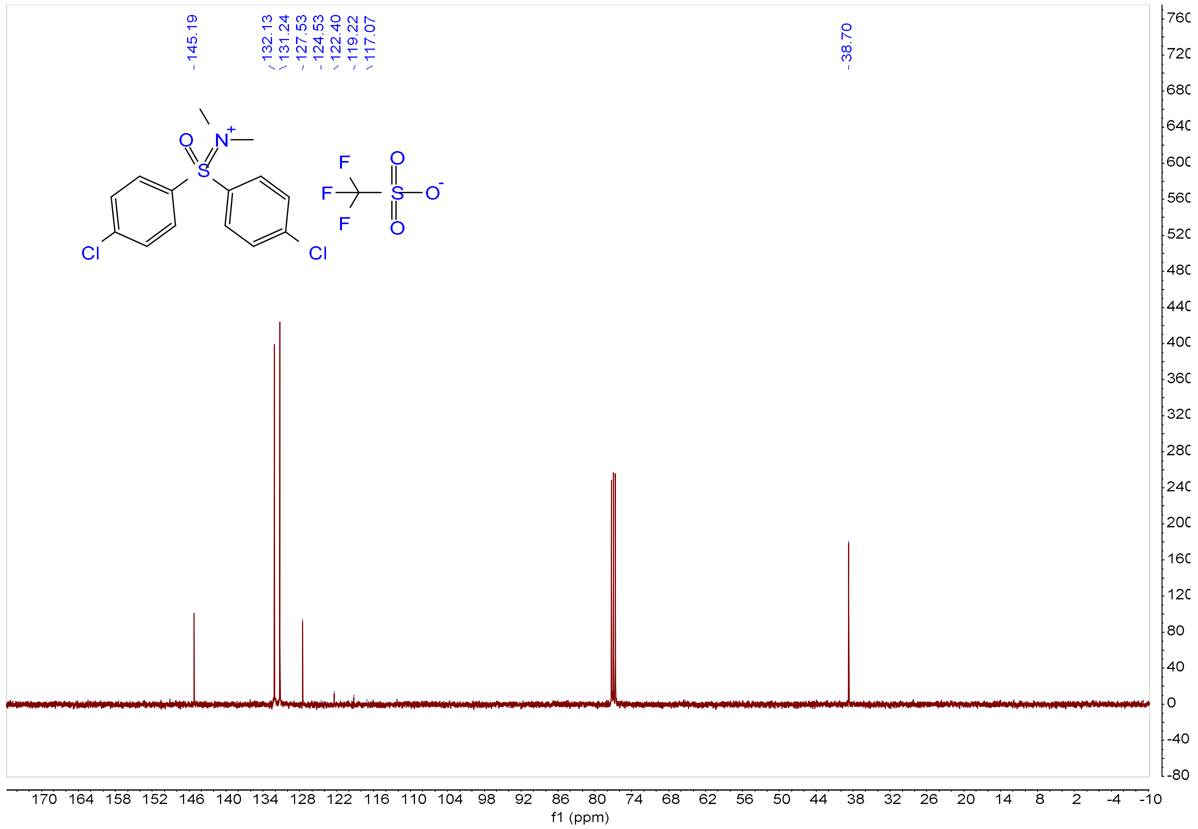

^{19}F NMR spectrum of compound **5b** (376 MHz, CDCl$_3$)

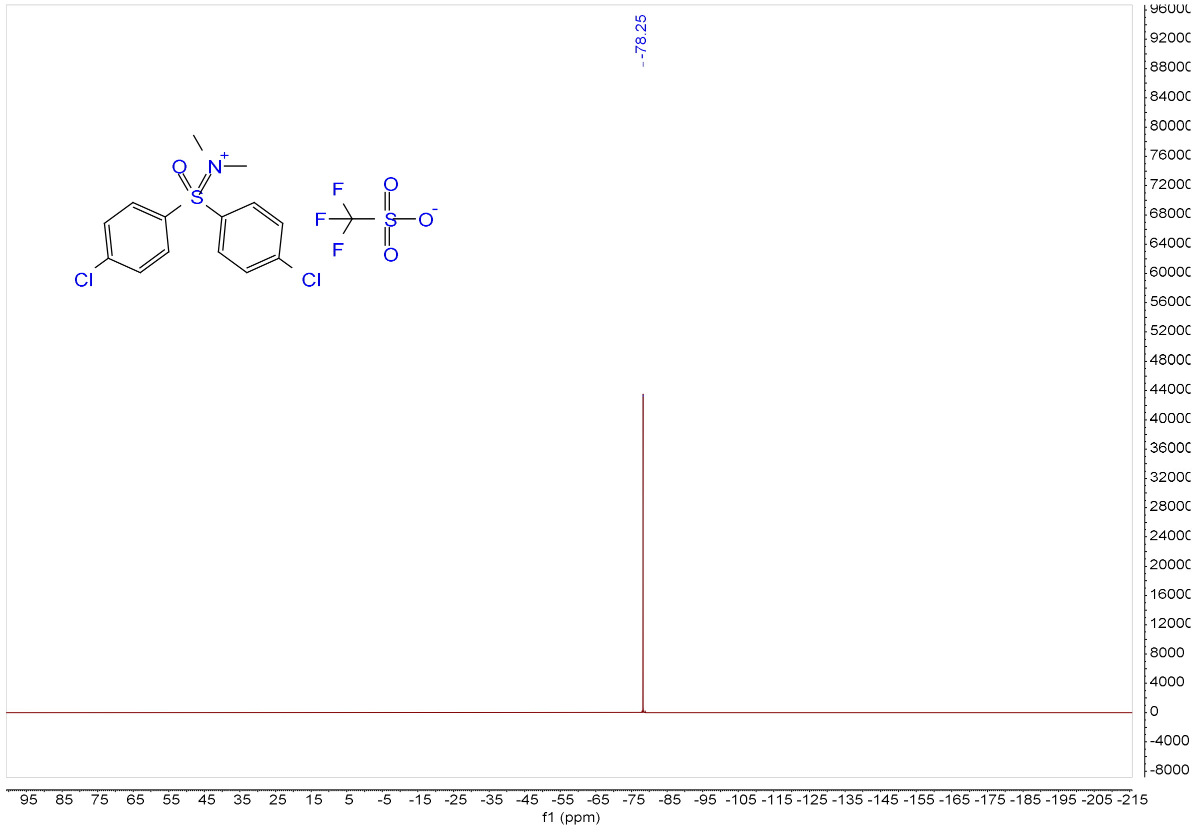

^{1}H NMR spectrum of compound **5c** (600 MHz, CDCl3)

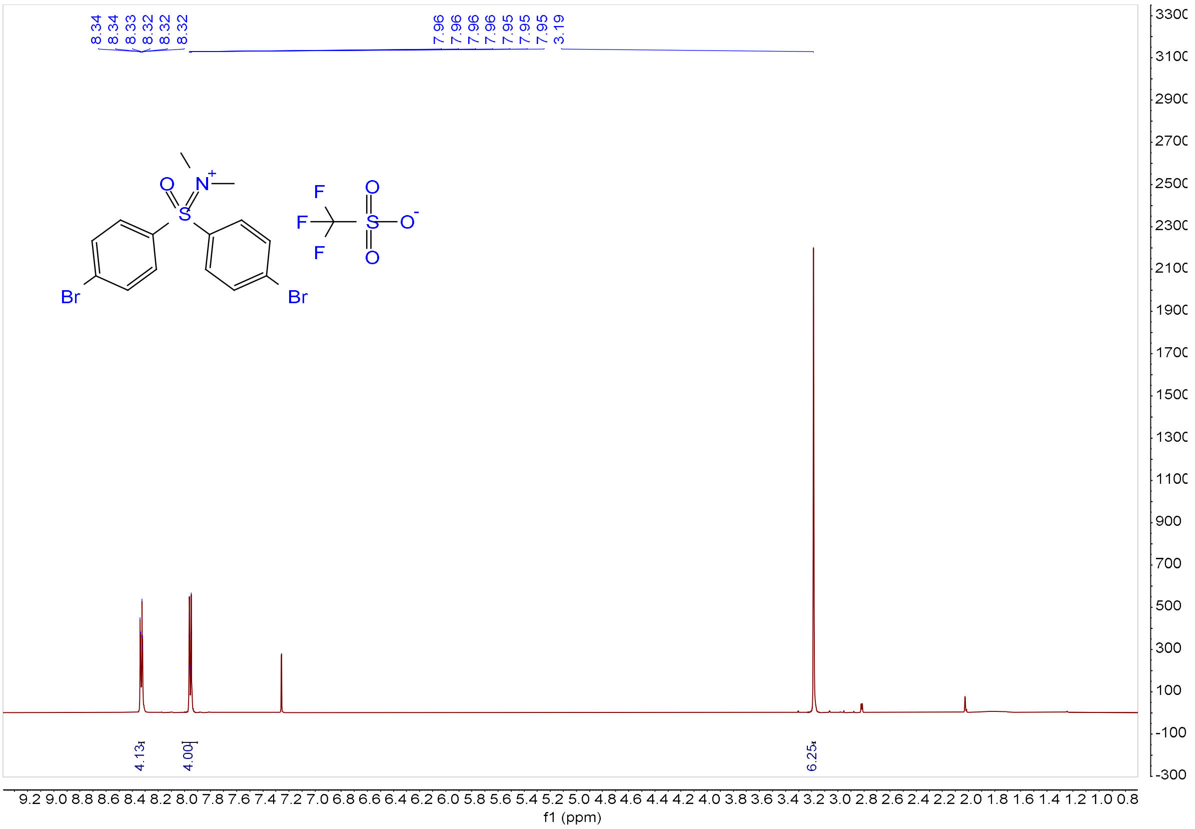

^{13}C NMR spectrum of compound **5c** (151 MHz, CDCl3)

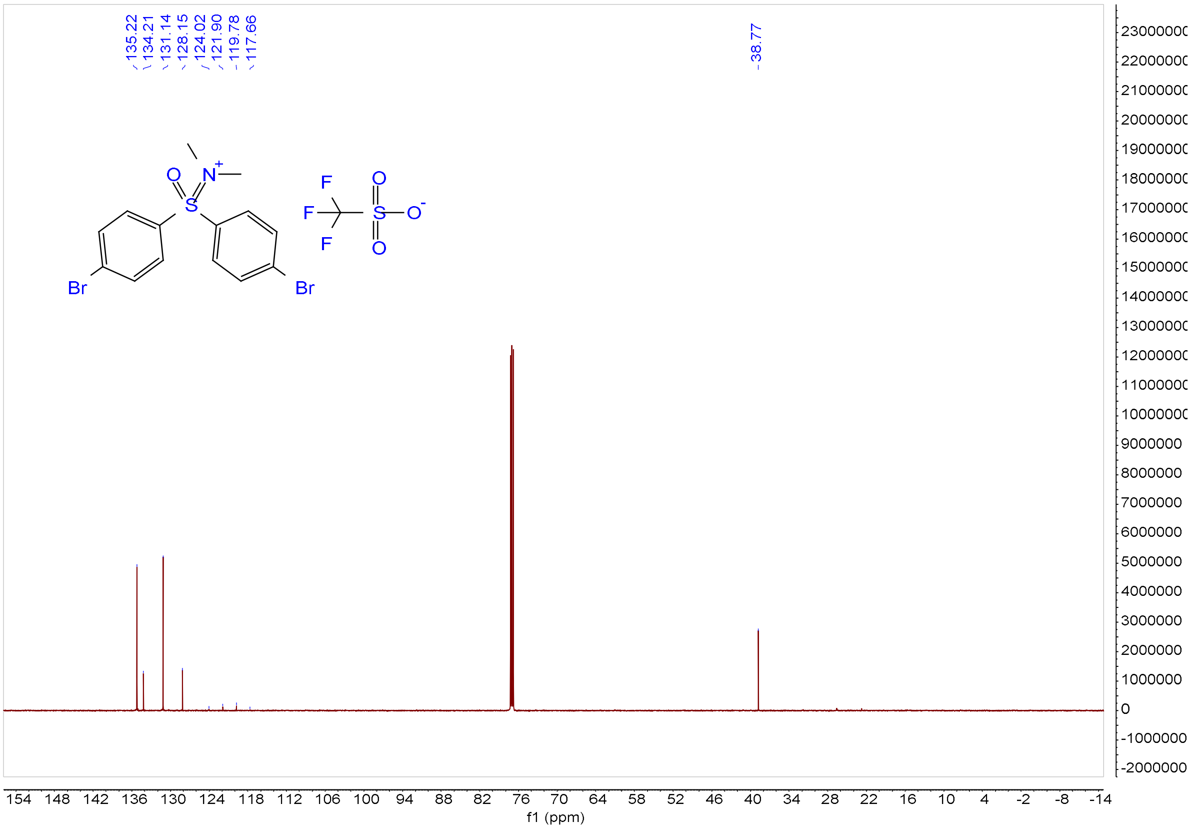

^{19}F NMR spectrum of compound **5c** (565 MHz, $CDCl_3$)

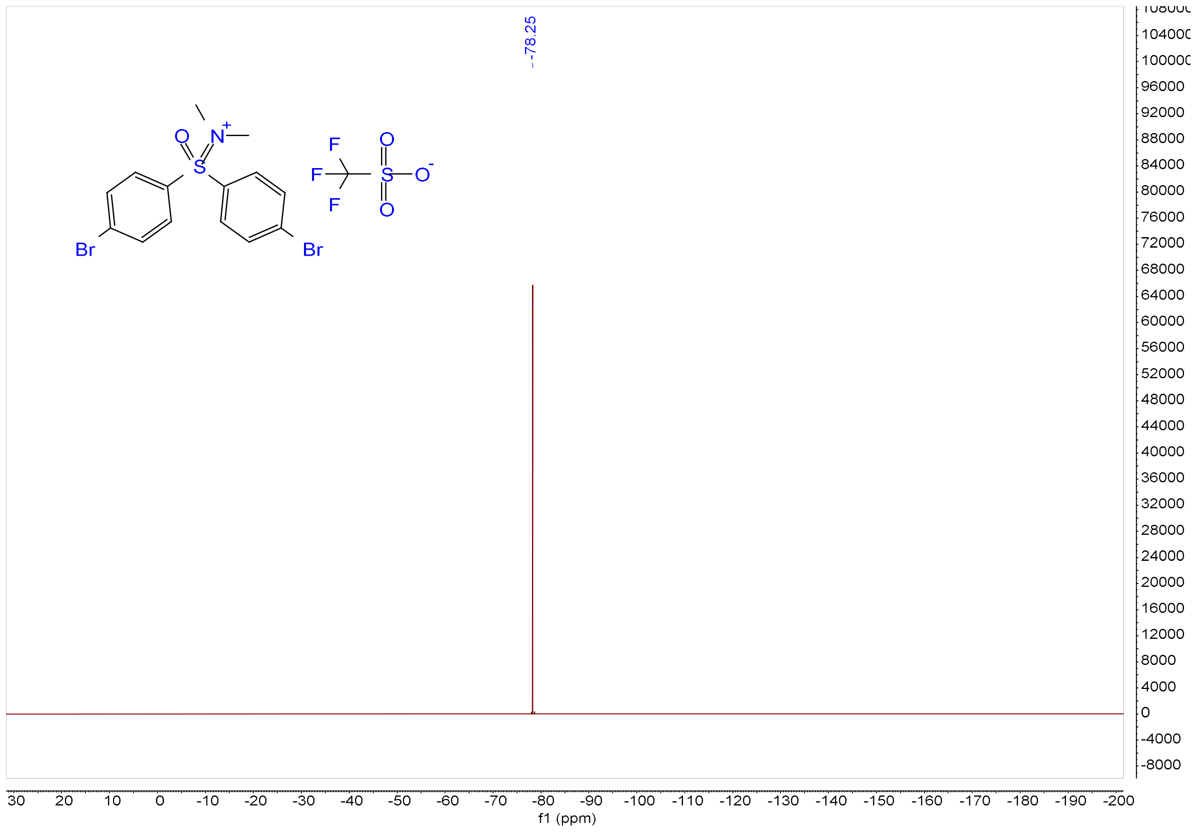

^{1}H NMR spectrum of compound **5d** (400 MHz, $CDCl_3$)

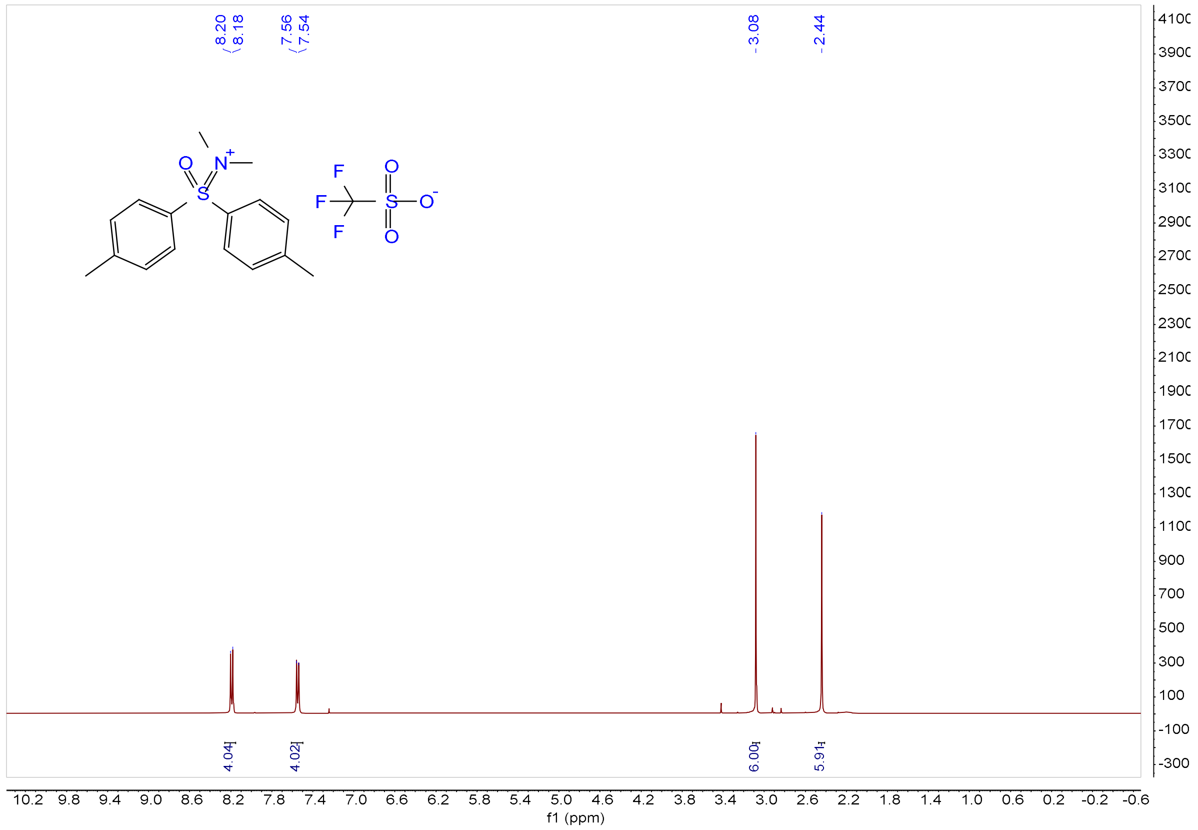

^{13}C NMR spectrum of compound **5d** (101 MHz, $CDCl_3$)

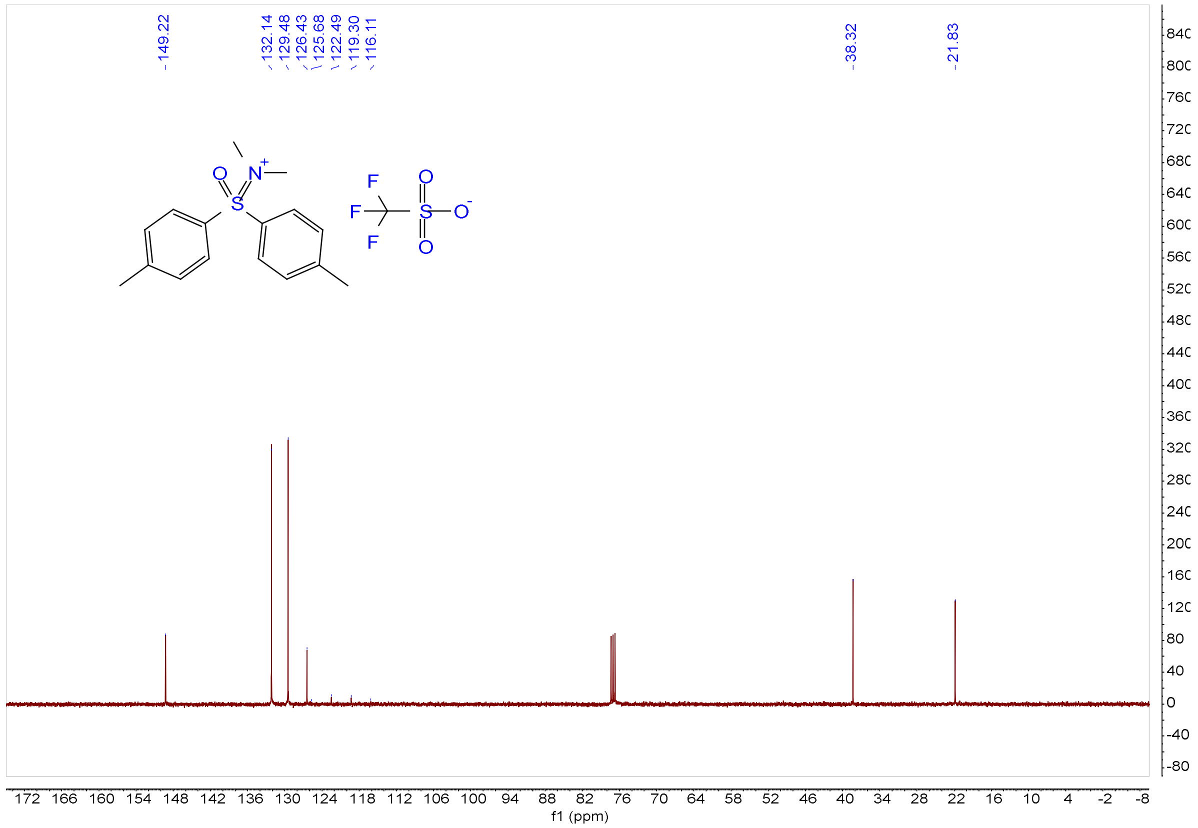

^{19}F NMR spectrum of compound **5d** (376 MHz, $CDCl_3$)

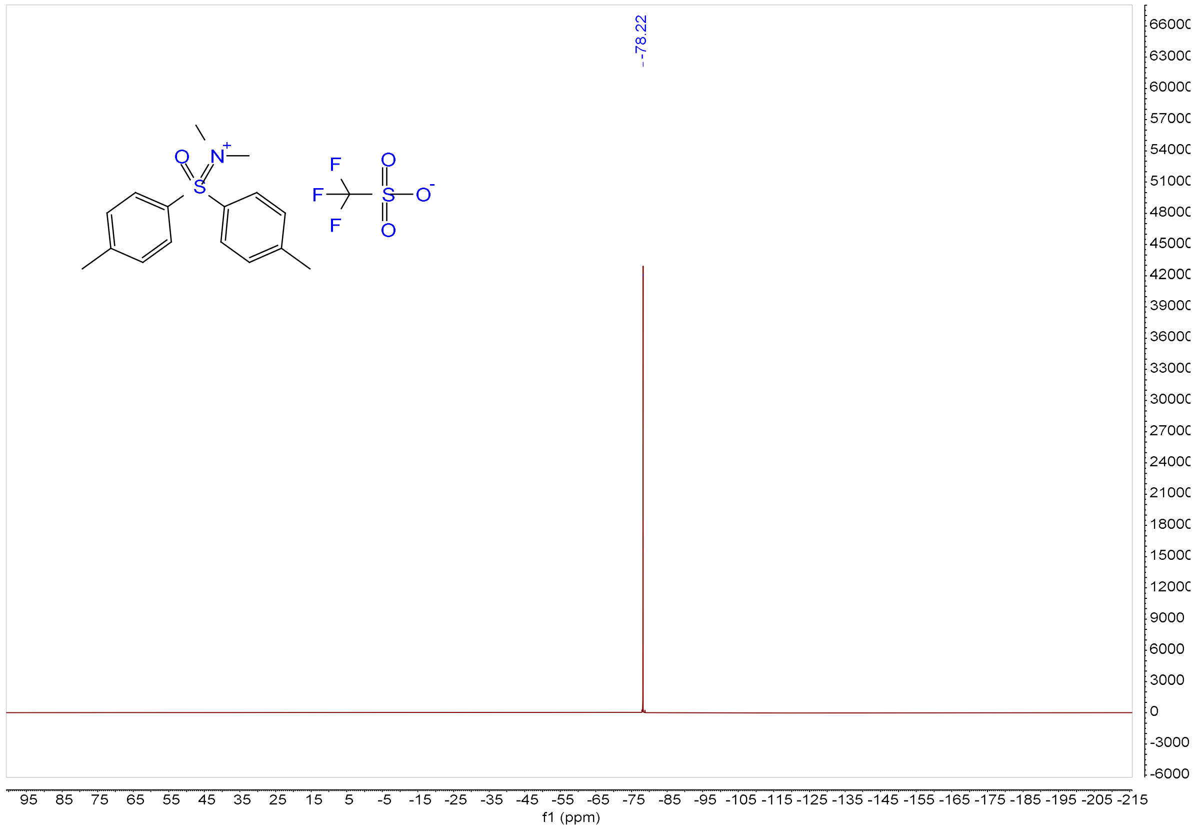

^{1}H NMR spectrum of compound **5e** (400 MHz, $CDCl_3$)

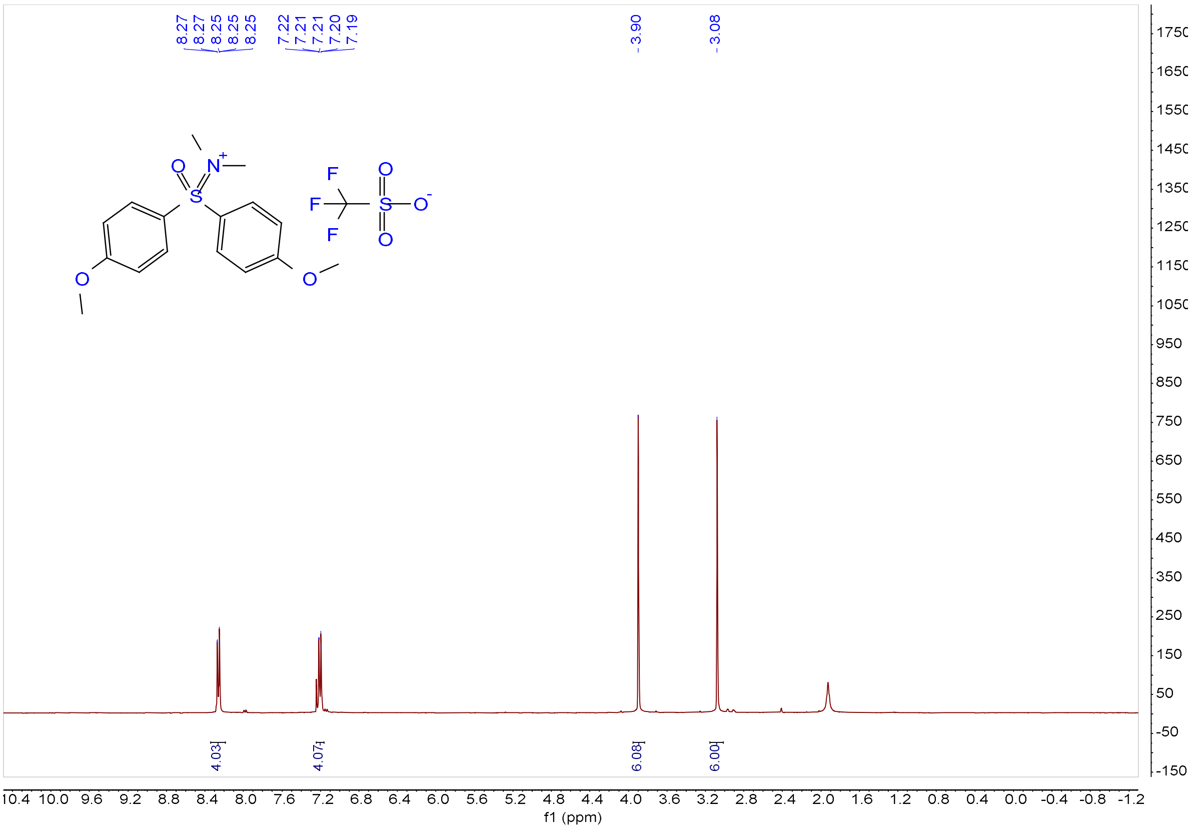

^{13}C NMR spectrum of compound **5e** (101 MHz, $CDCl_3$)

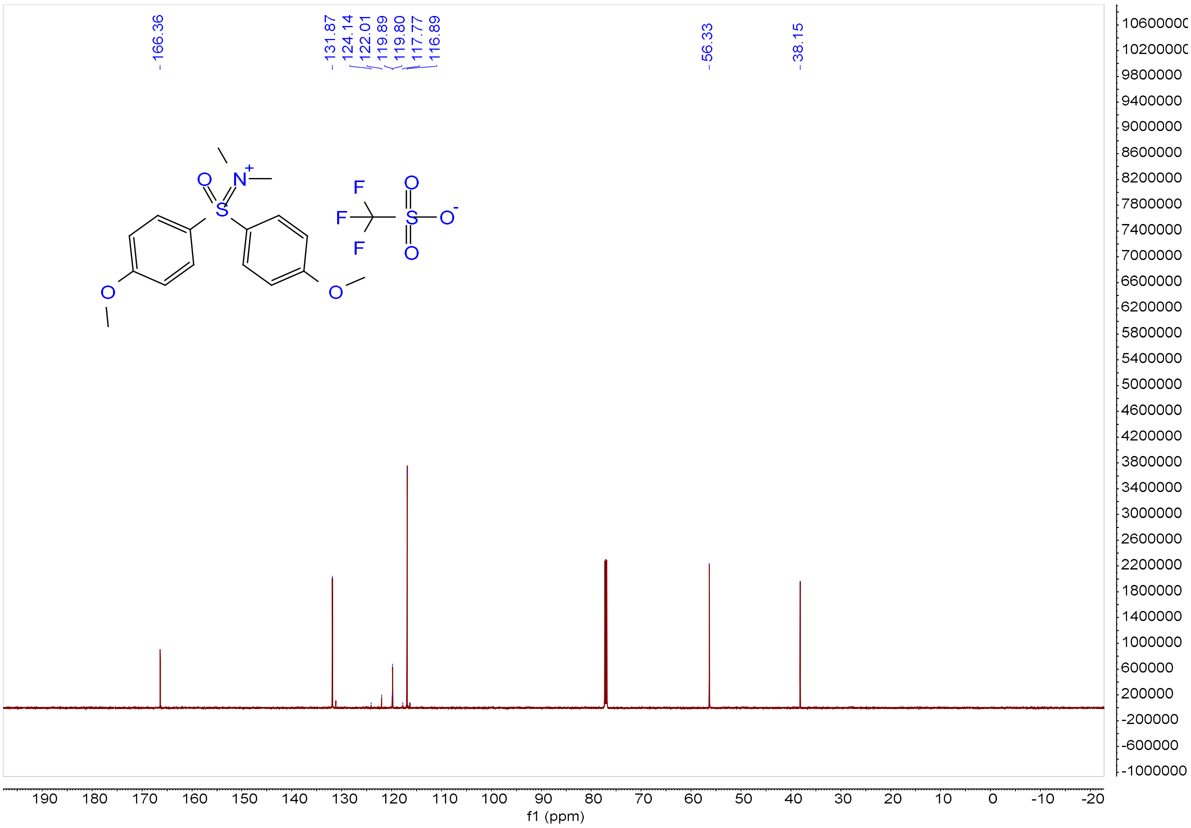

^{19}F NMR spectrum of compound **5e** (376 MHz, $CDCl_3$)

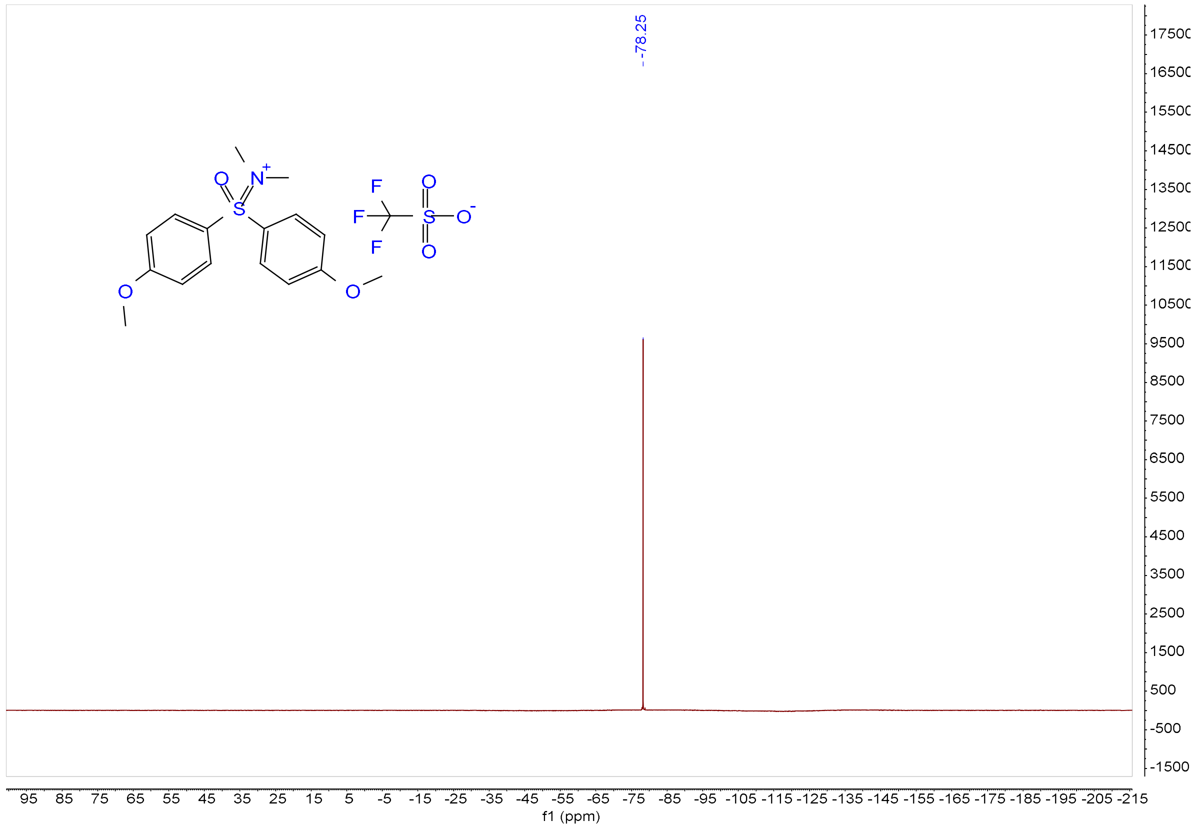

^{1}H NMR spectrum of compound **5f** (600 MHz, DMSO-d_6)

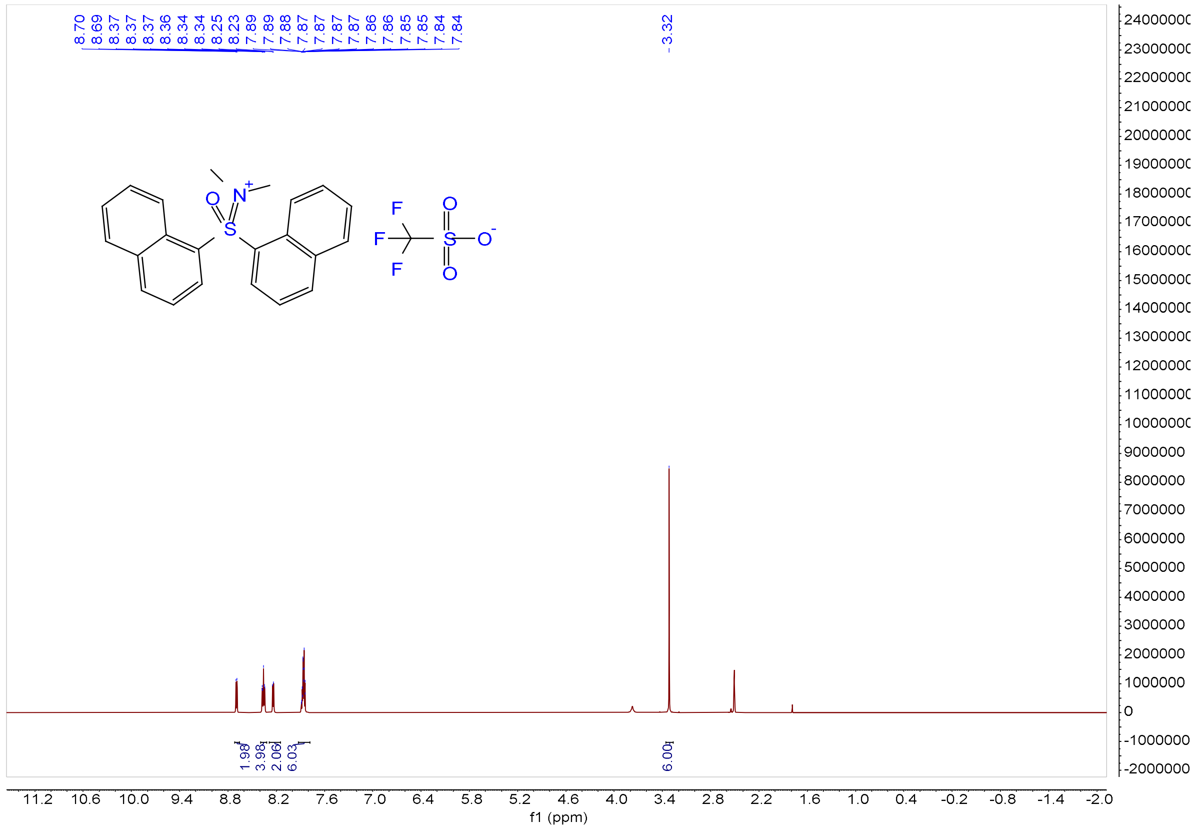

^{13}C NMR spectrum of compound **5f** (151 MHz, DMSO-d_6)

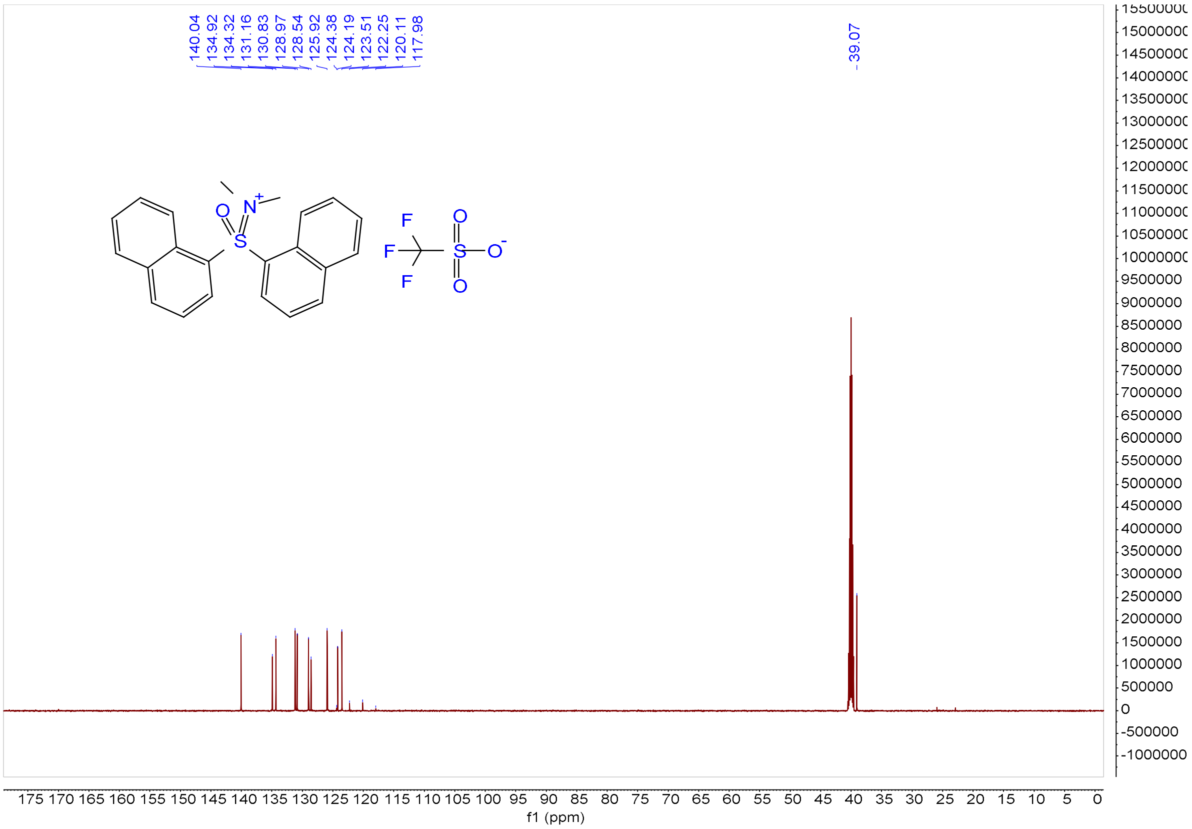

^{19}F NMR spectrum of compound **5f** (565 MHz, DMSO-d_6)

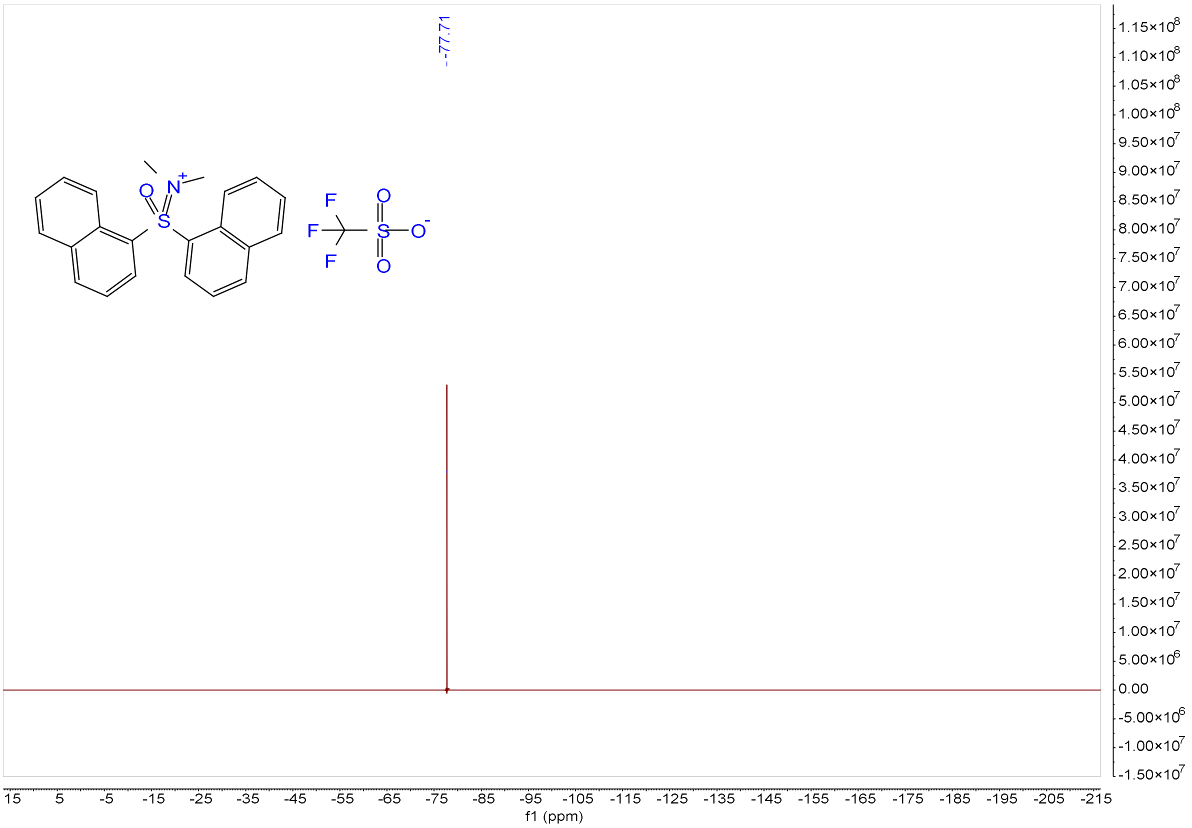

^{1}H NMR spectrum of compound **5g** (400 MHz, $CDCl_3$)

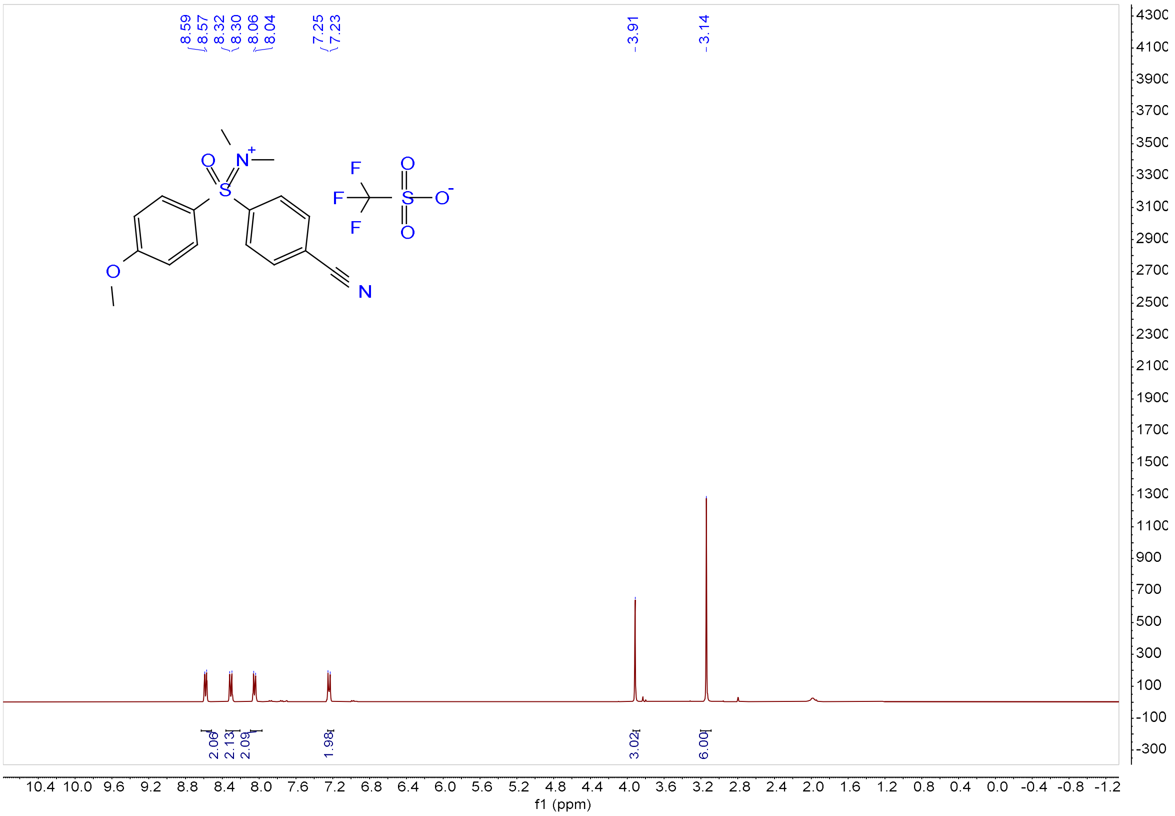

^{13}C NMR spectrum of compound **5g** (101 MHz, $CDCl_3$)

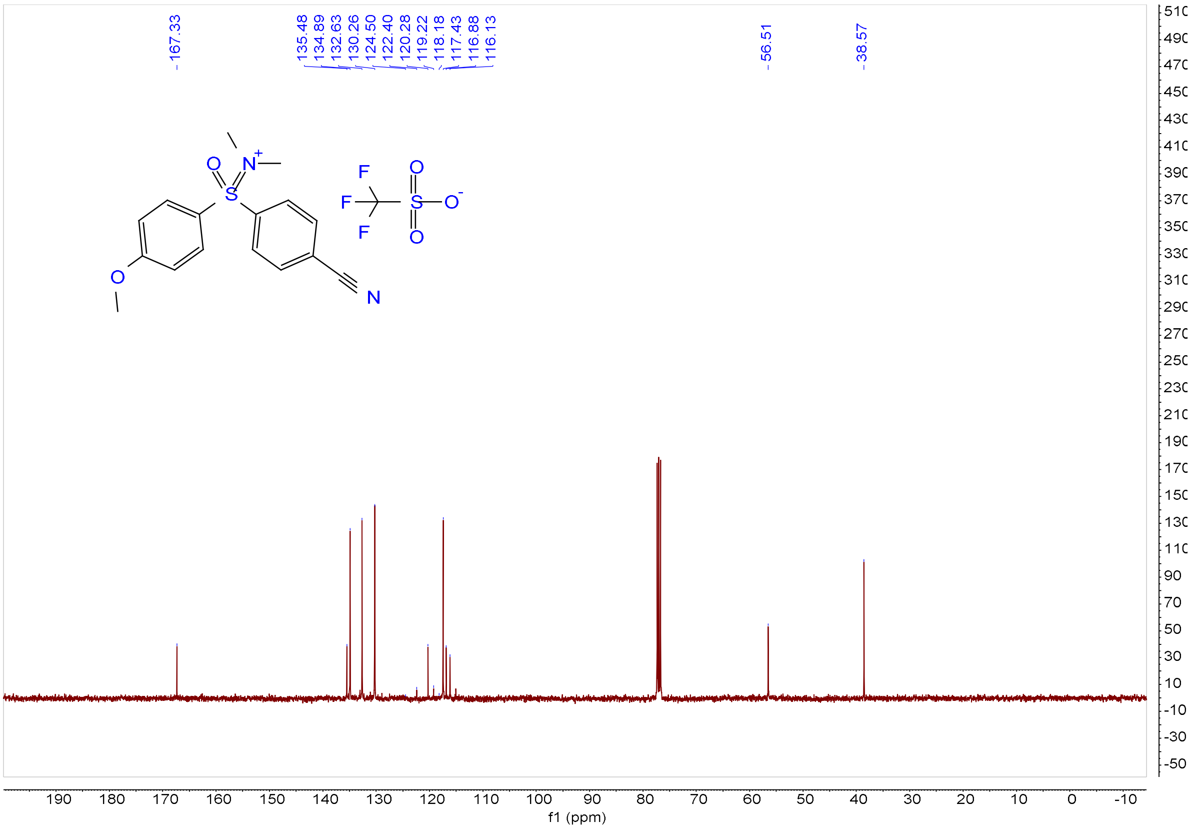

^{19}F NMR spectrum of compound **5g** (376 MHz, CDCl$_3$)

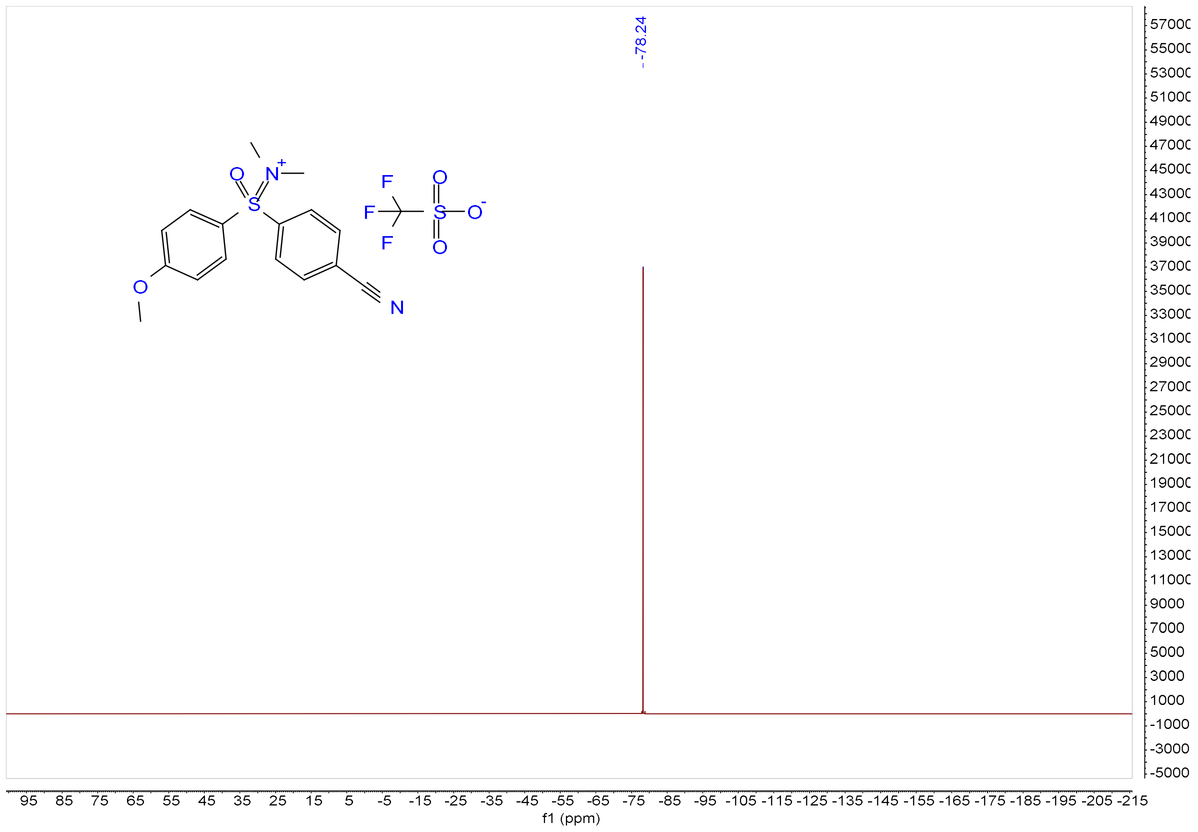

^{1}H NMR spectrum of compound **5h** (600 MHz, CDCl$_3$)

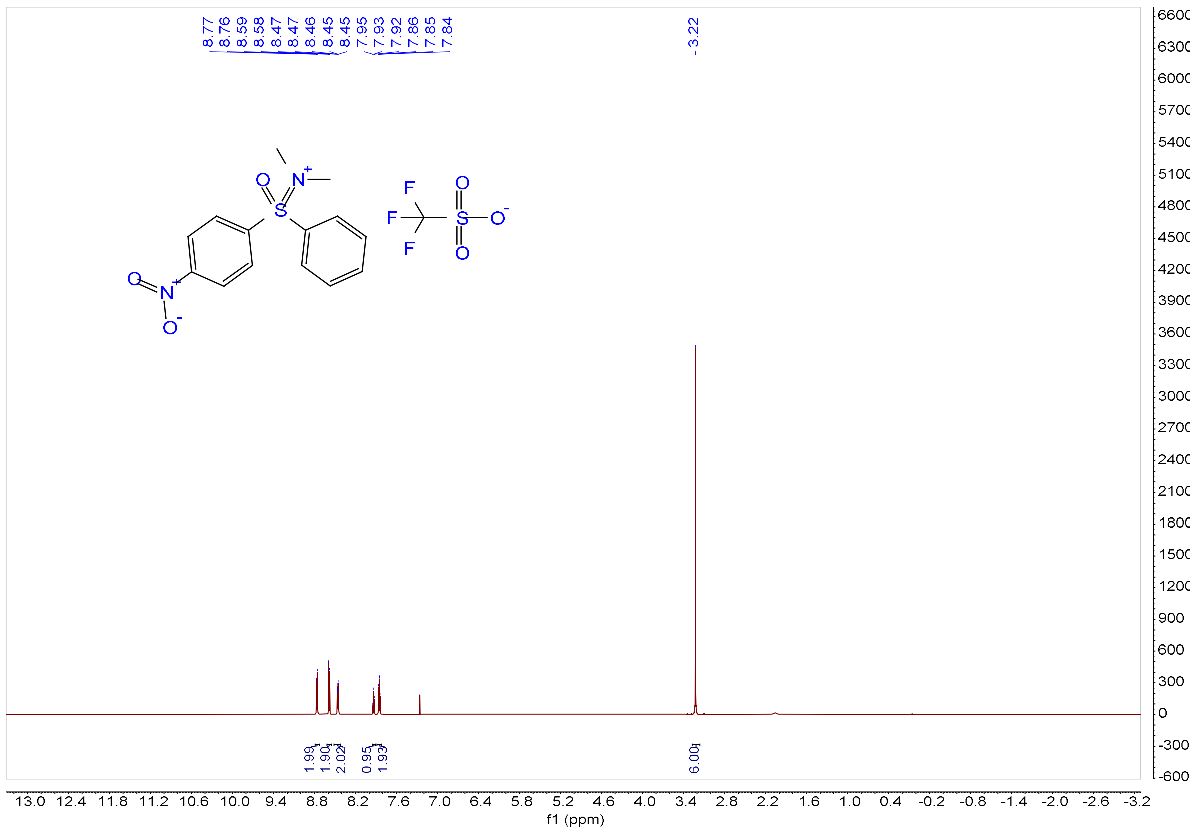

^{13}C NMR spectrum of compound **5h** (151 MHz, $CDCl_3$)

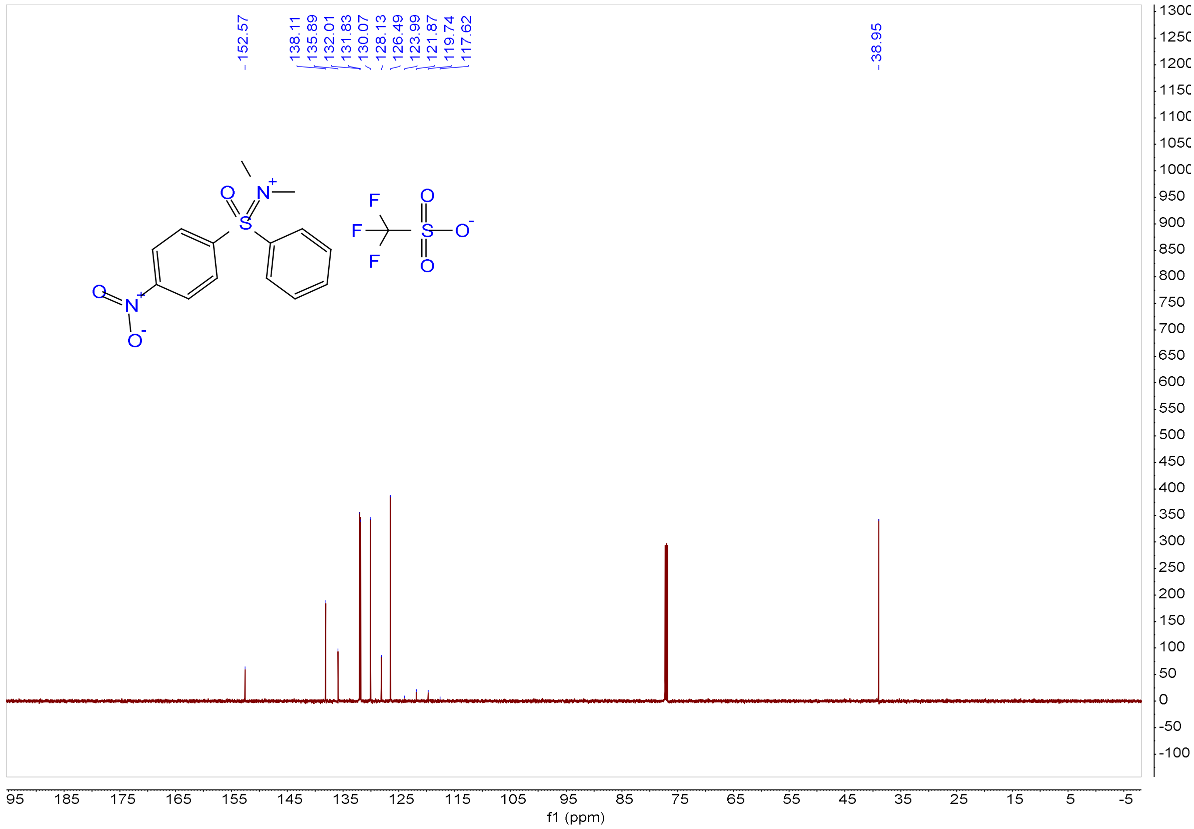

^{19}F NMR spectrum of compound **5h** (565 MHz, $CDCl_3$)

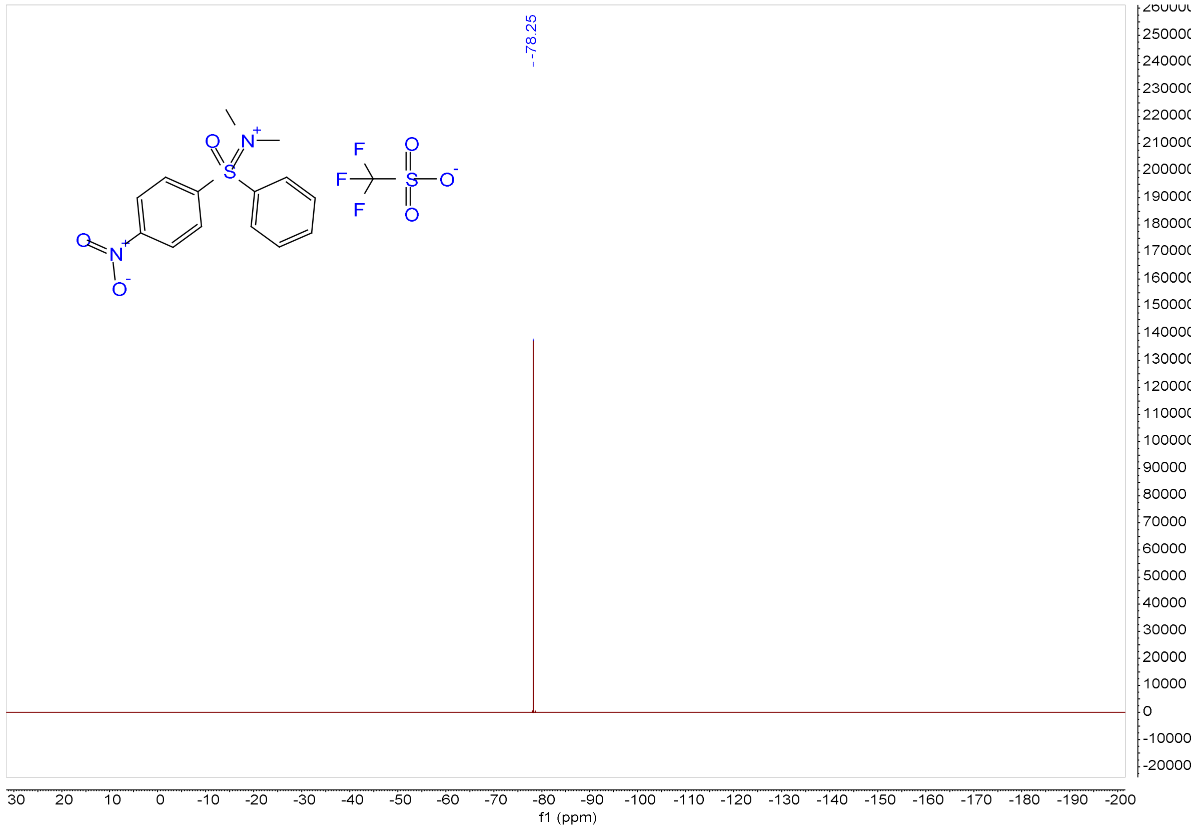

^{1}H NMR spectrum of compound **5i** (600 MHz, $CDCl_3$)

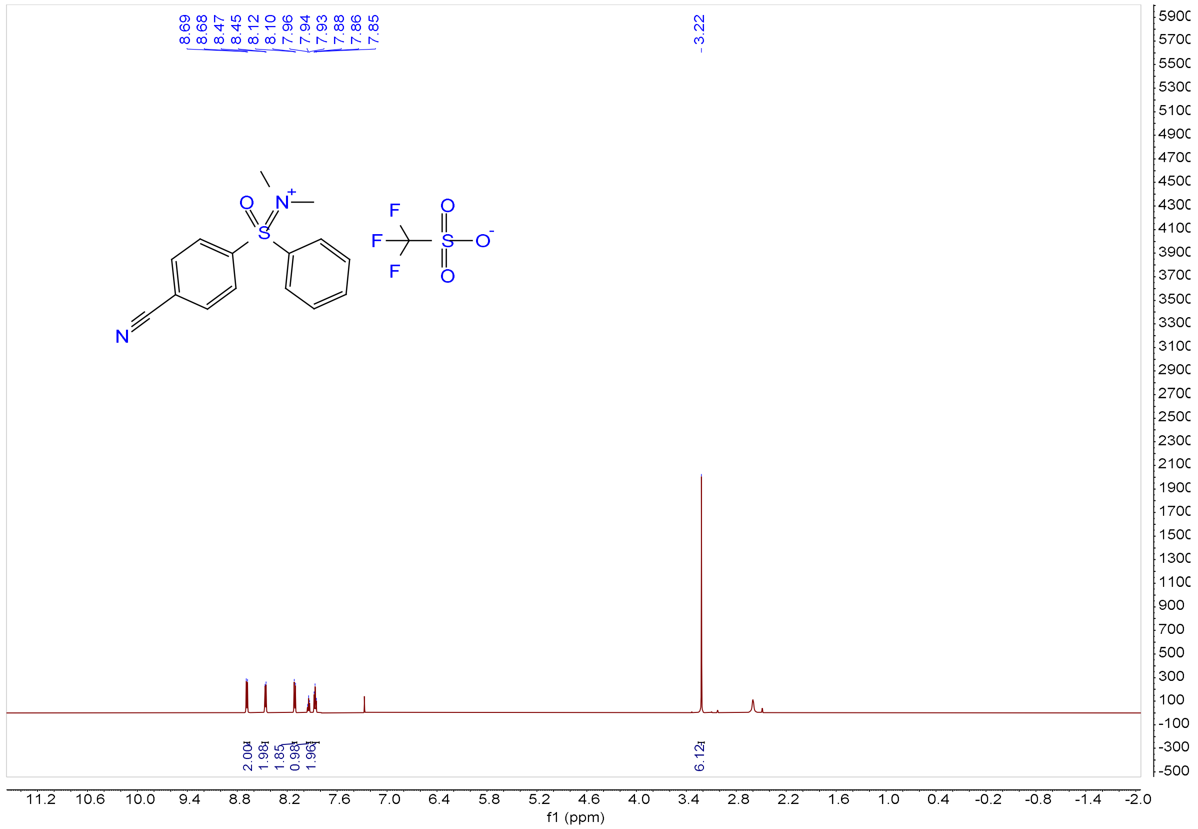

^{13}C NMR spectrum of compound **5i** (151 MHz, $CDCl_3$)

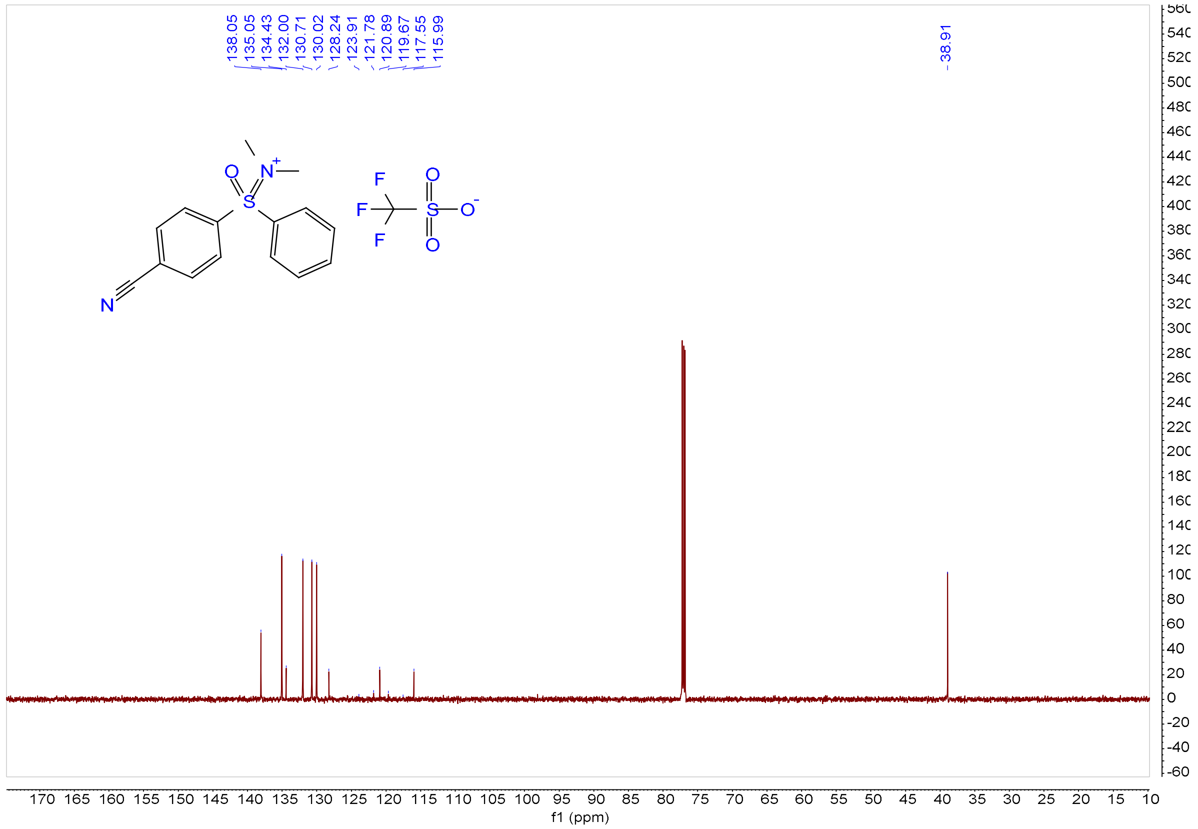

^{19}F NMR spectrum of compound **5i** (565 MHz, $CDCl_3$)

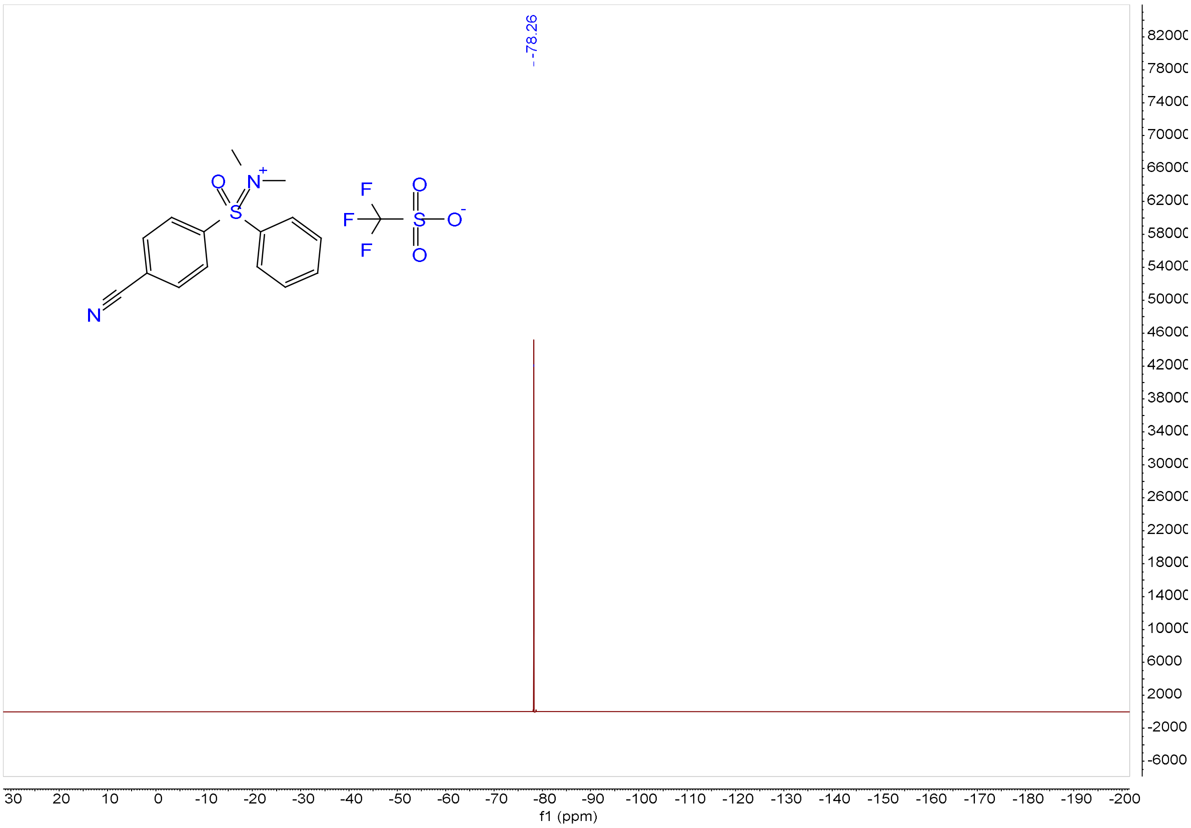

^{1}H NMR spectrum of compound **5j** (600 MHz, $CDCl_3$)

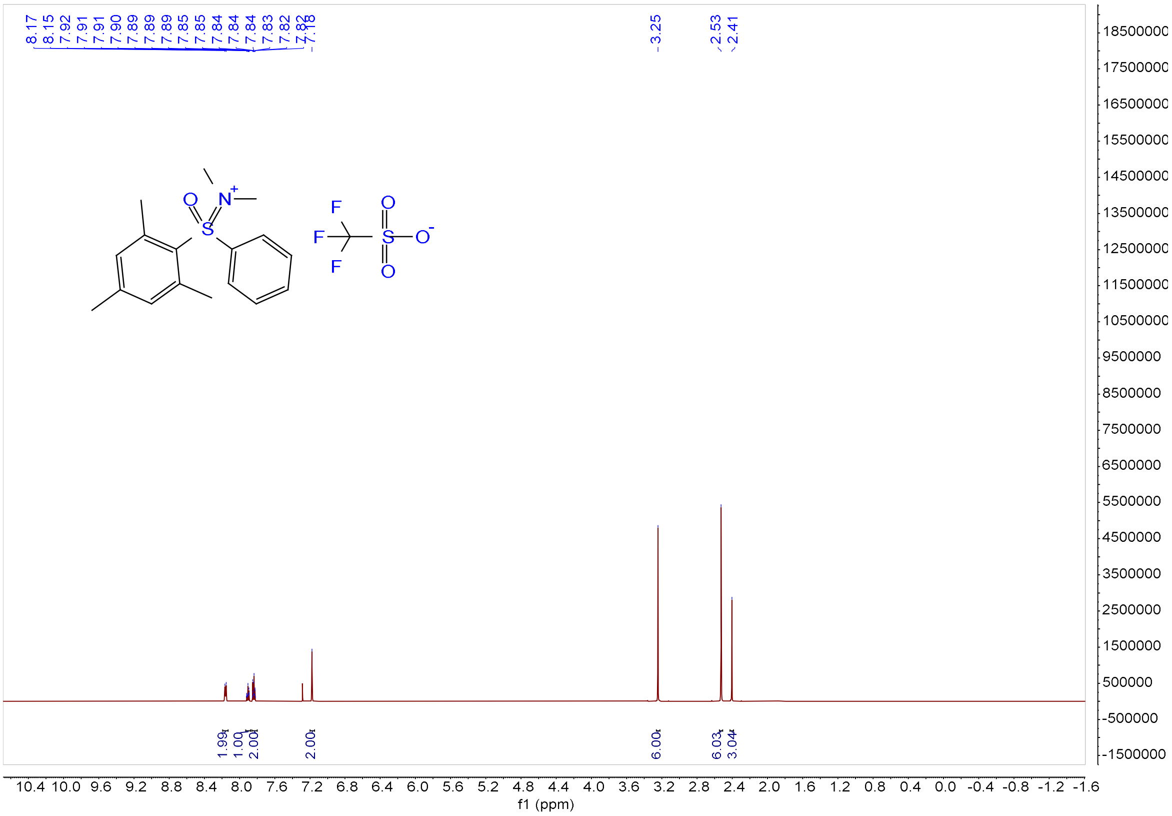

^{13}C NMR spectrum of compound **5j** (151 MHz, $CDCl_3$)

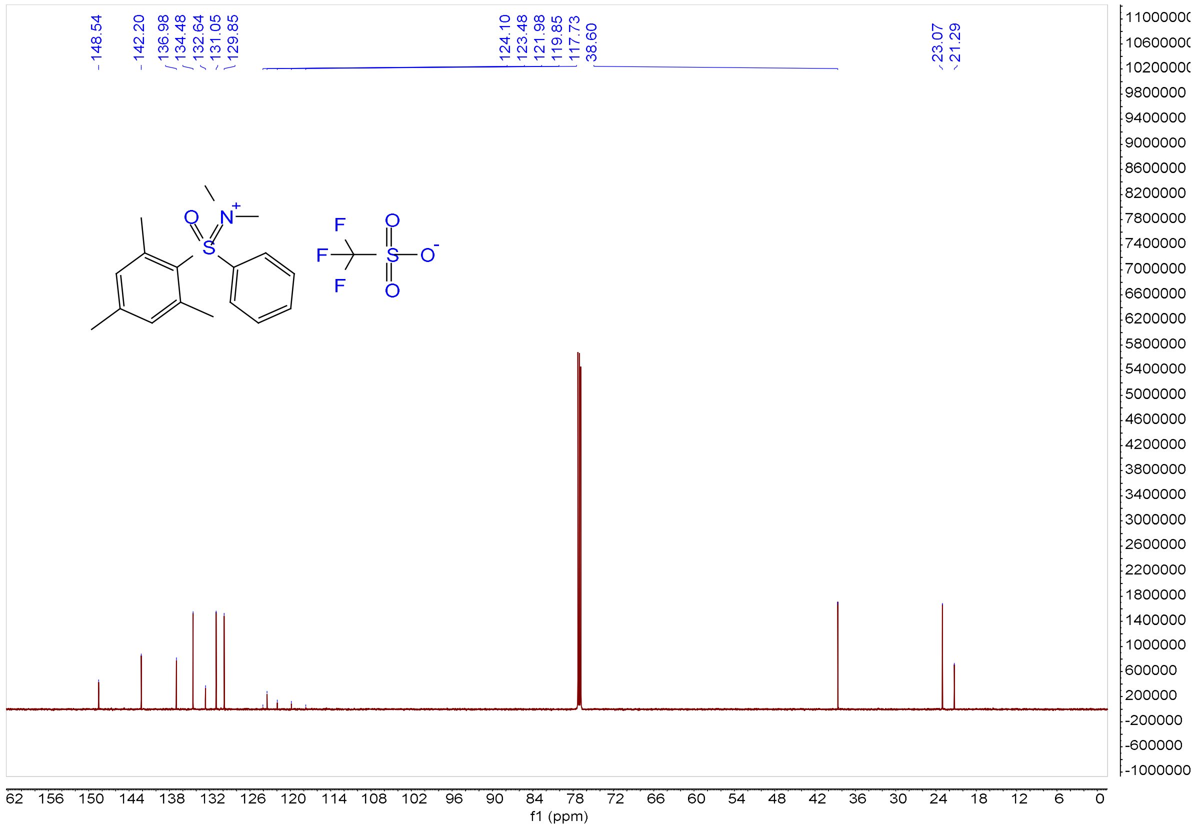

^{19}F NMR spectrum of compound **5j** (565 MHz, $CDCl_3$)

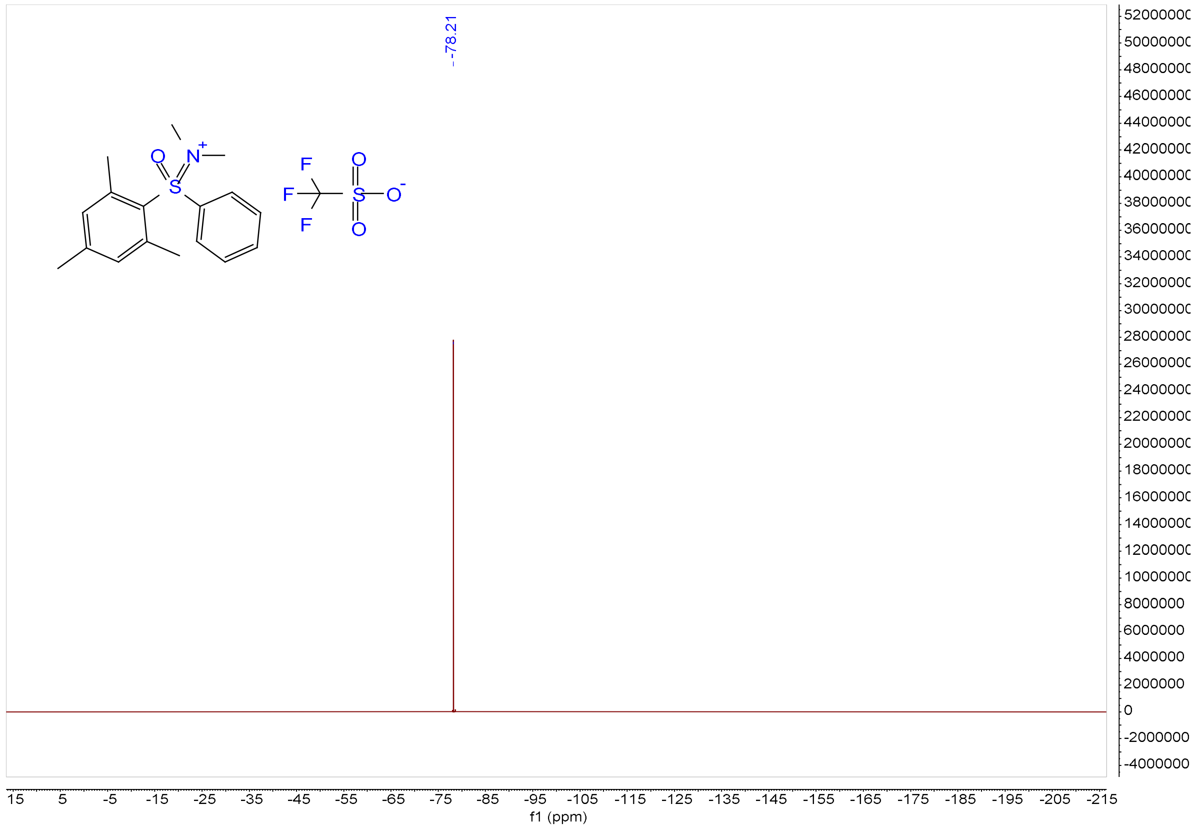

^{1}H NMR spectrum of compound **5k** (600 MHz, $CDCl_3$)

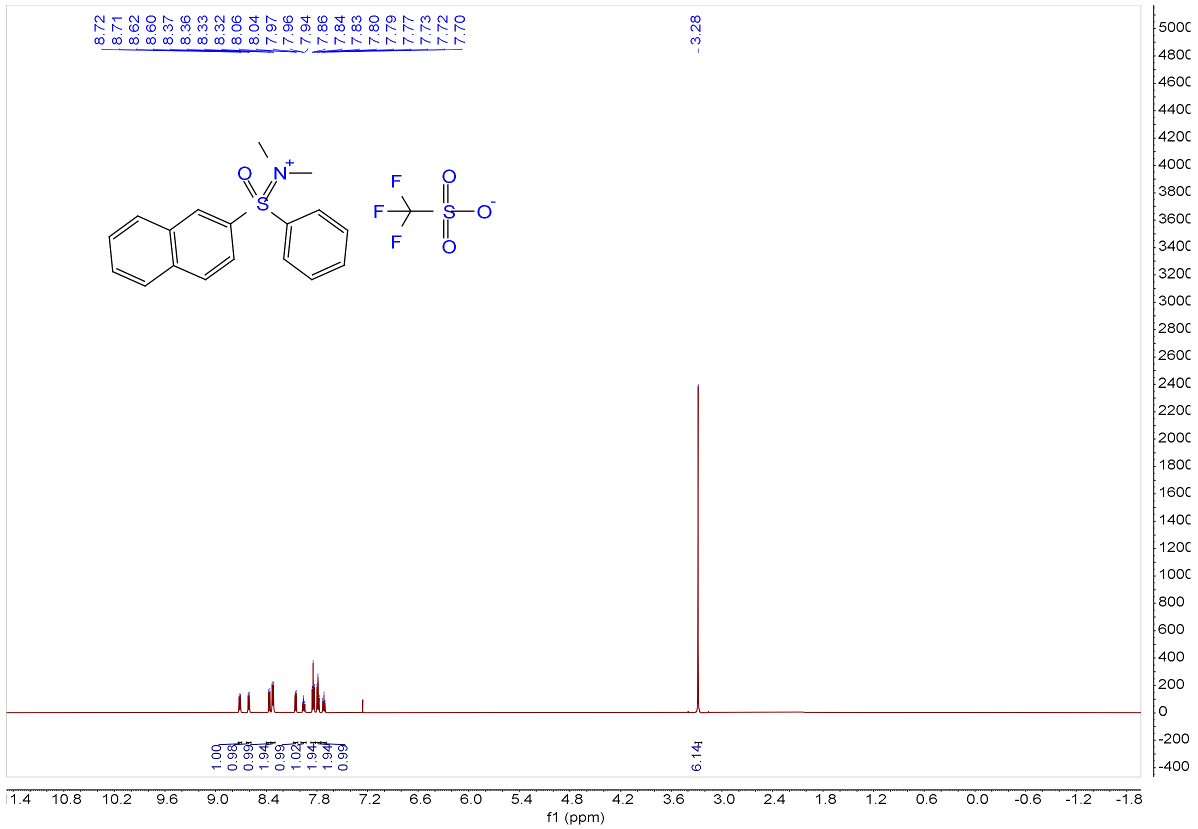

^{13}C NMR spectrum of compound **5k** (151 MHz, $CDCl_3$)

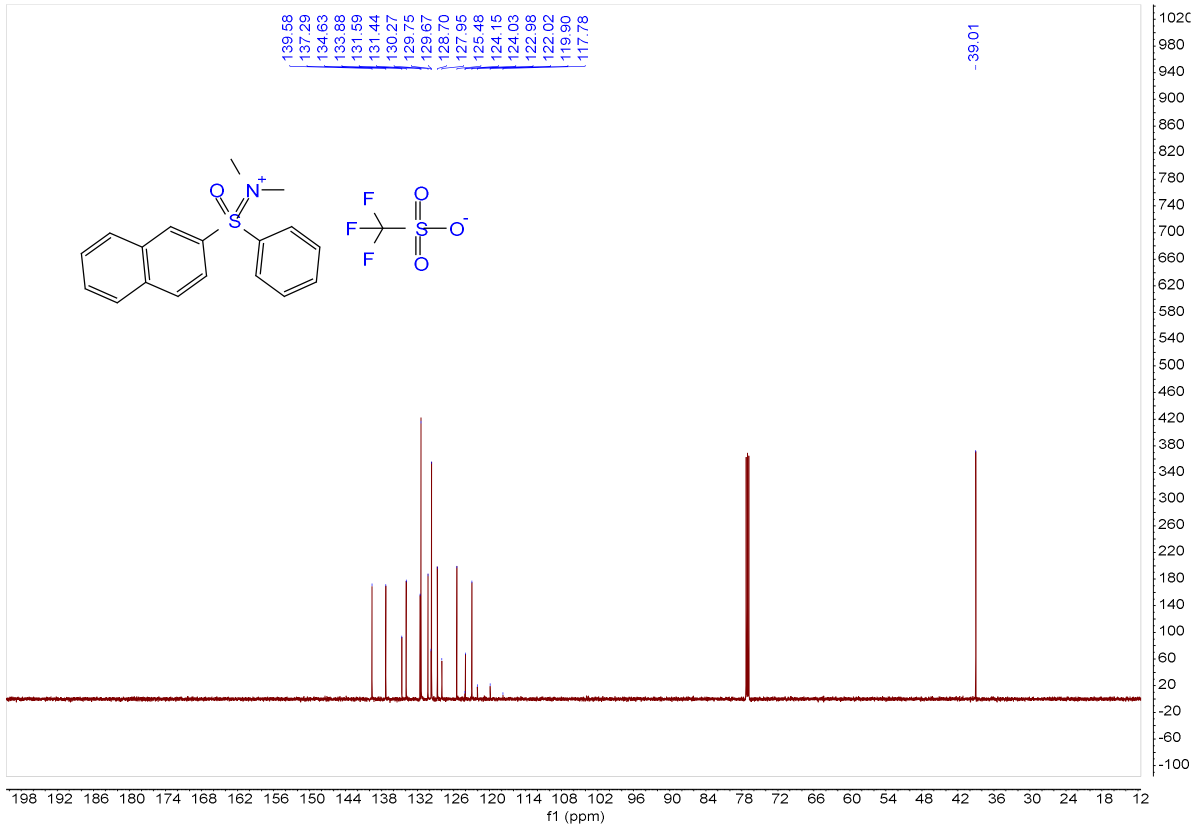

^{19}F NMR spectrum of compound **5k** (565 MHz, $CDCl_3$)

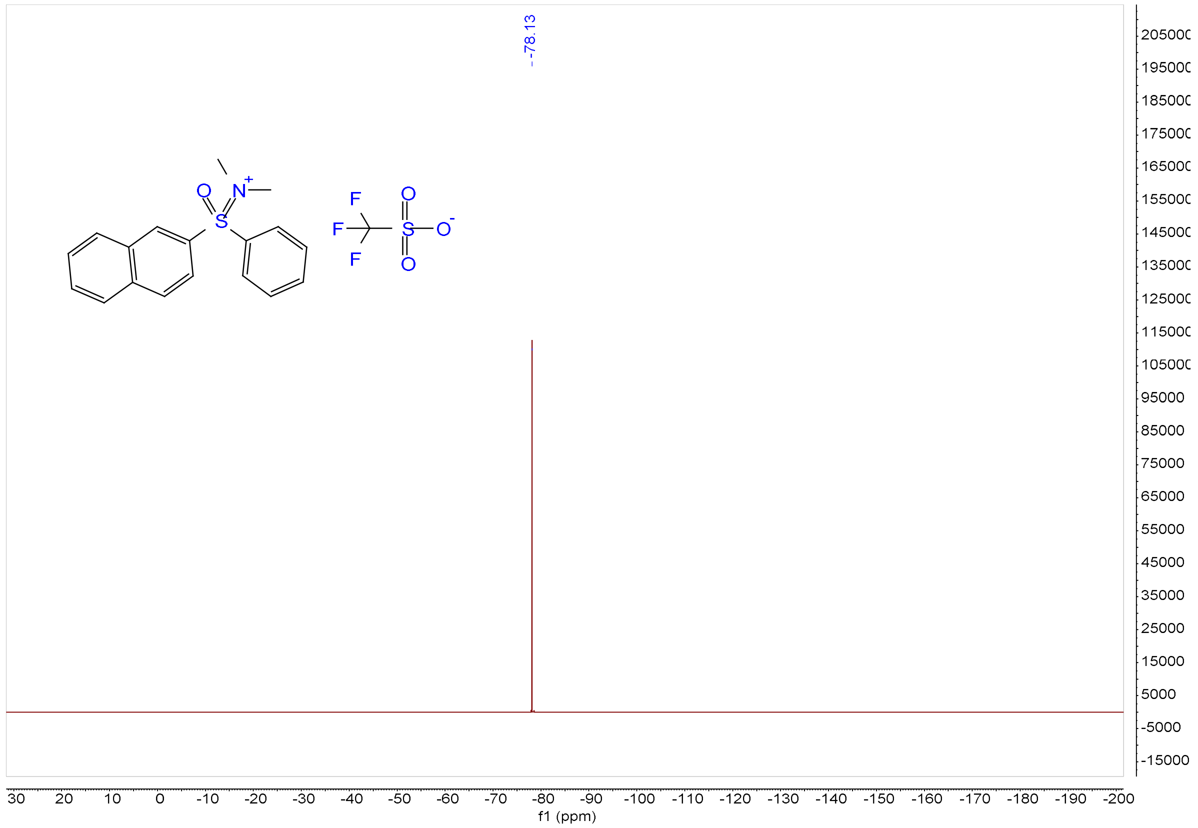

^{1}H NMR spectrum of compound **5l** (600 MHz, DMSO-d_6)

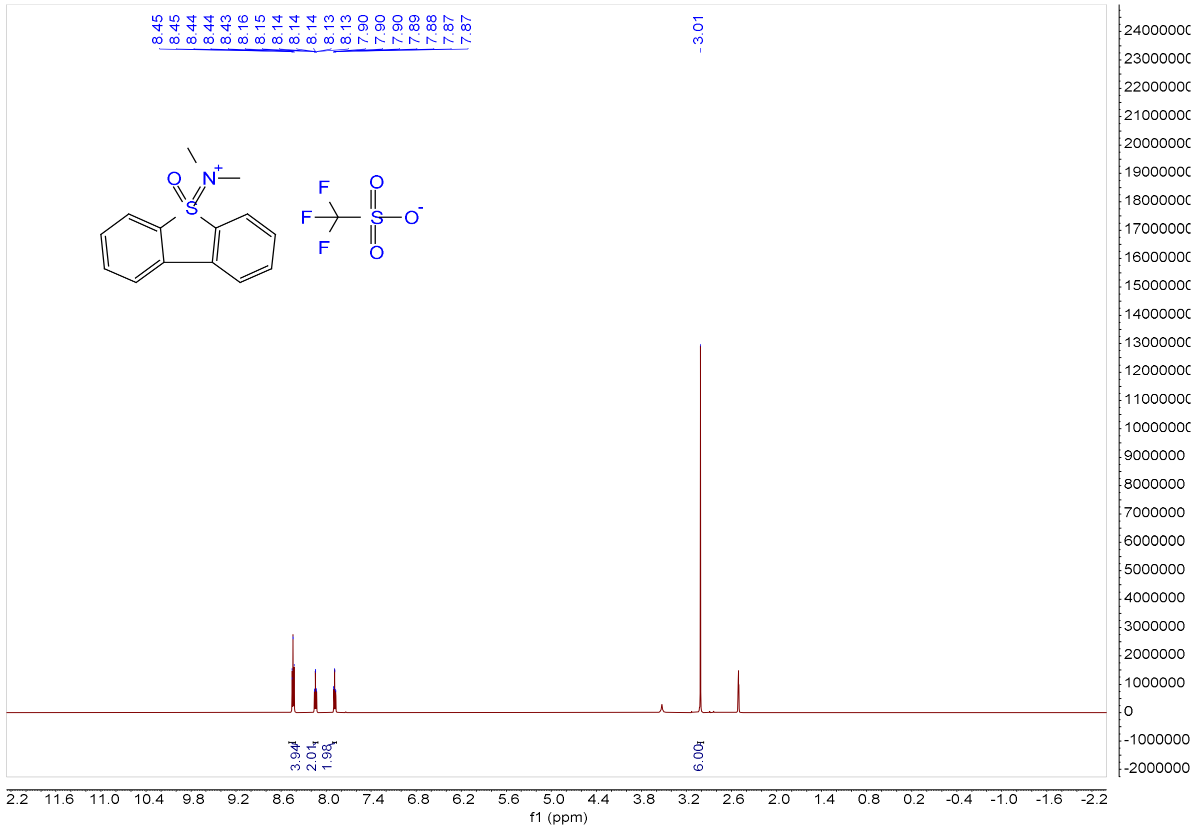

^{13}C NMR spectrum of compound **5l** (151 MHz, DMSO-d_6)

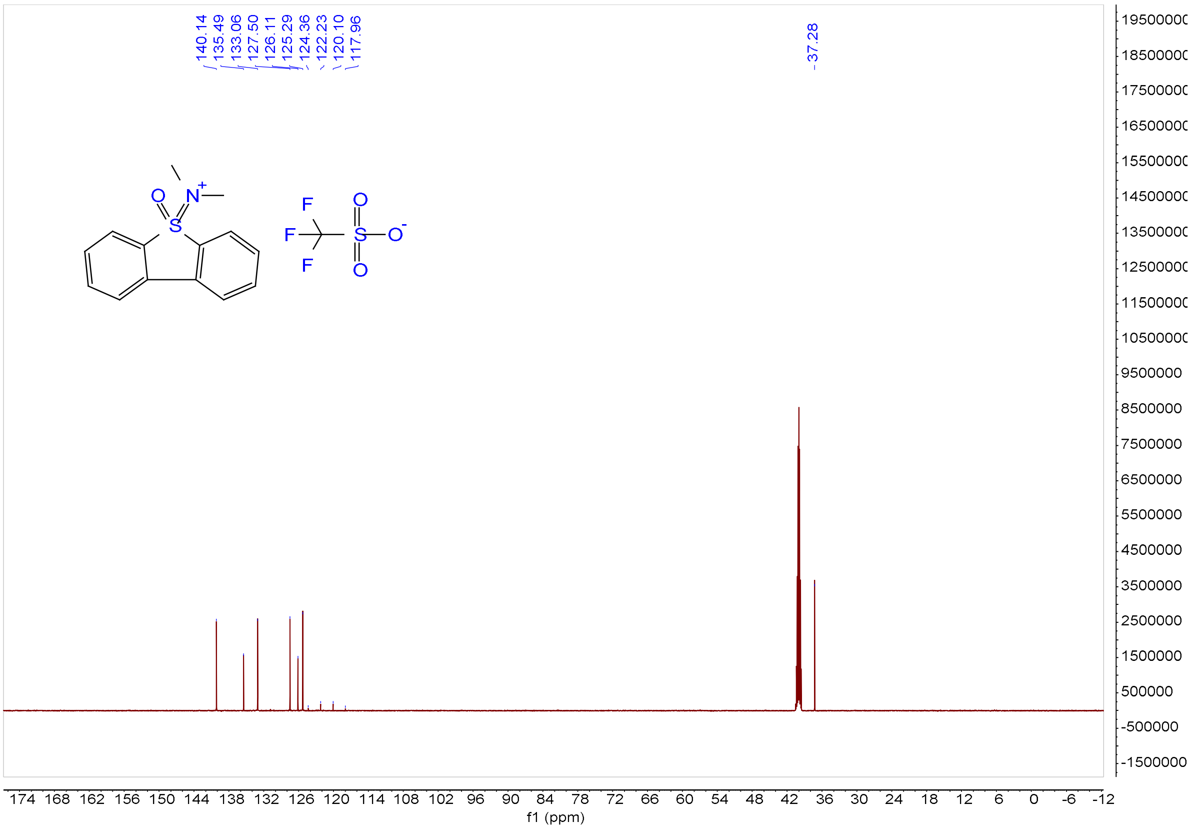

^{19}F NMR spectrum of compound **5l** (565 MHz, DMSO-d_6)

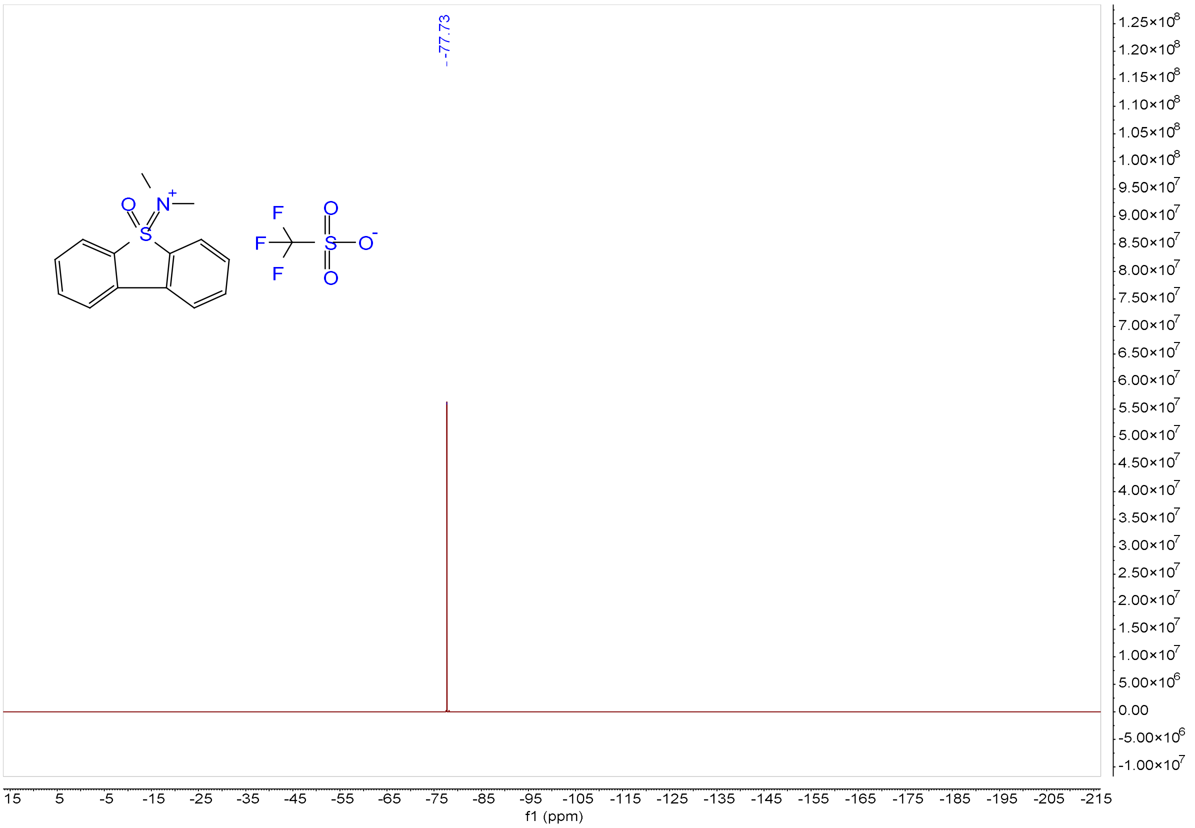

^{1}H NMR spectrum of compound **8aa** (400 MHz, $CDCl_3$)

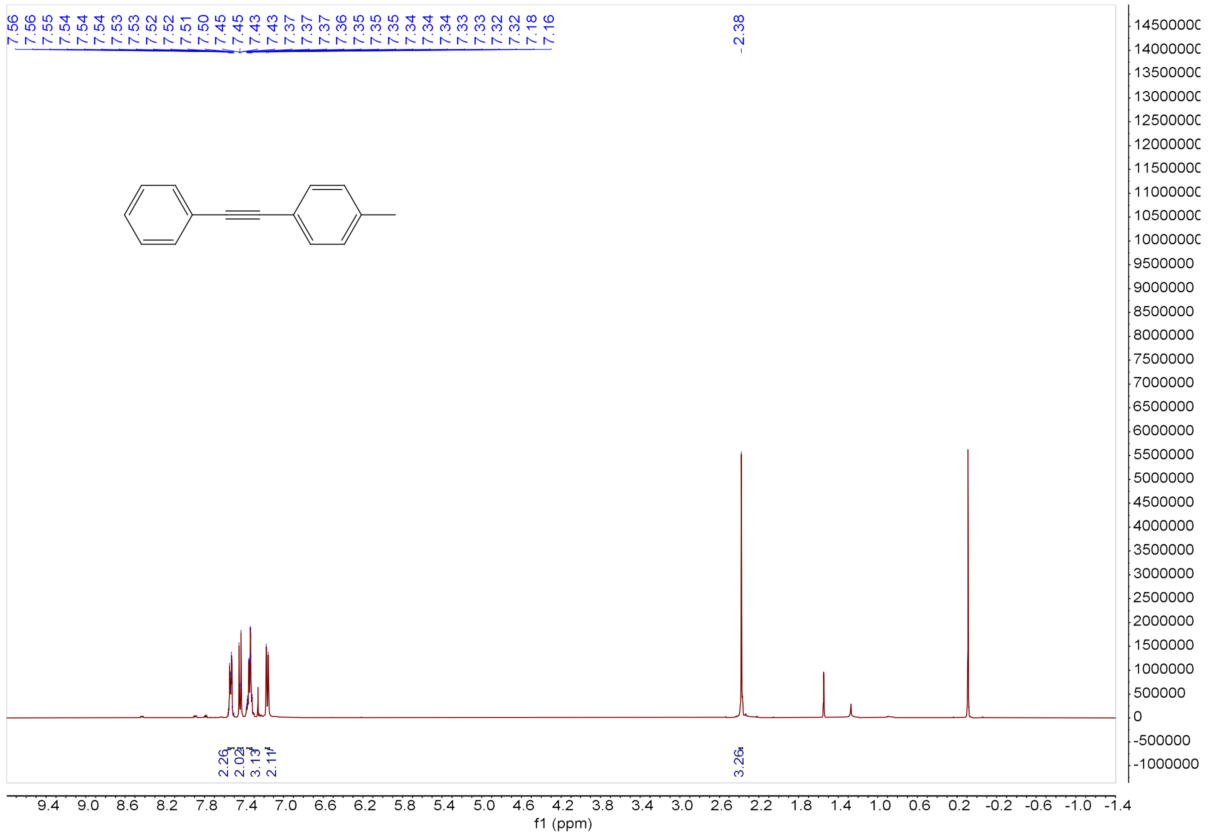

^{13}C NMR spectrum of compound **8aa** (101 MHz, $CDCl_3$)

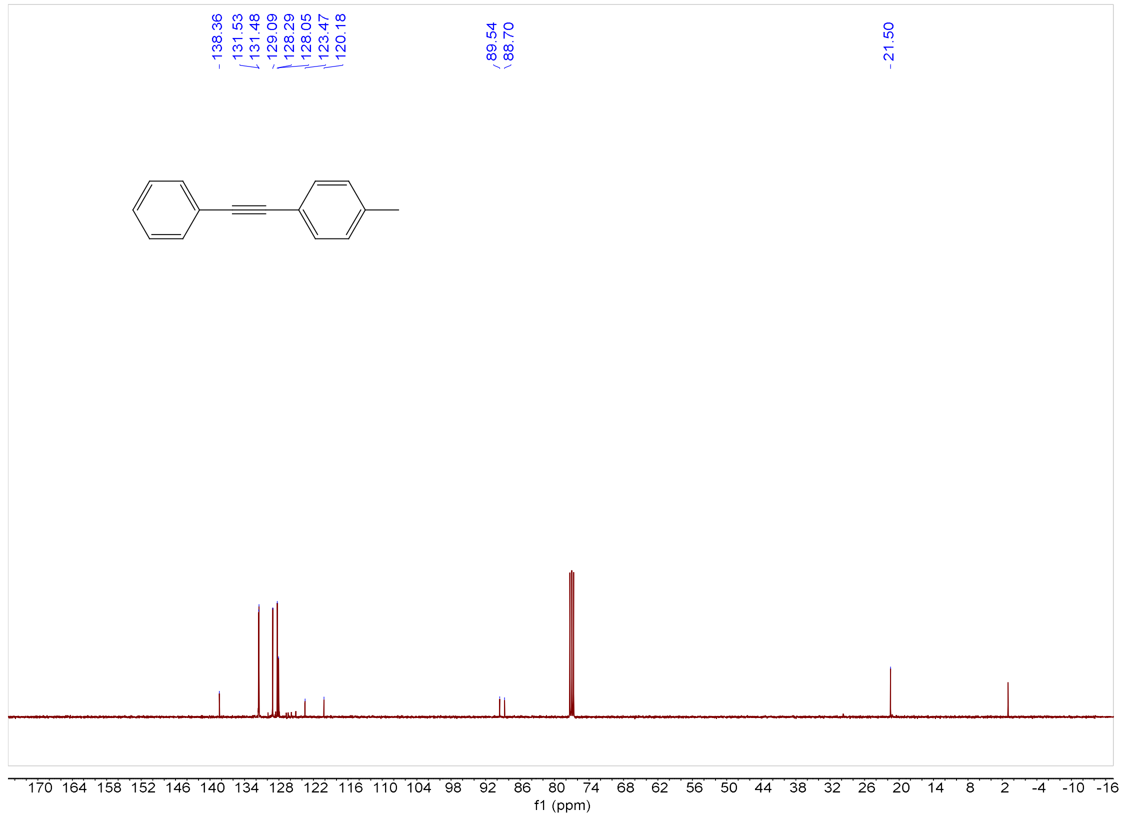

^{1}H NMR spectrum of compound **8ba** (400 MHz, $CDCl_3$)

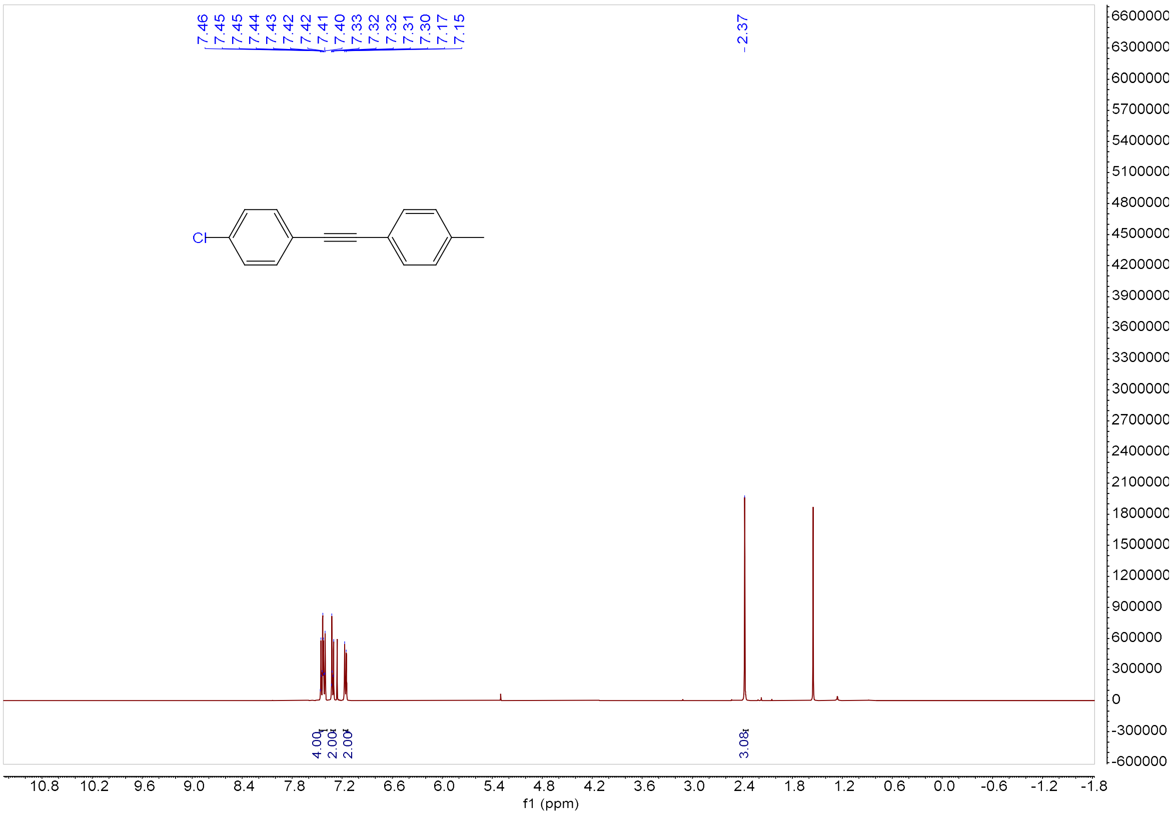

^{13}C NMR spectrum of compound **8ba** (101 MHz, $CDCl_3$)

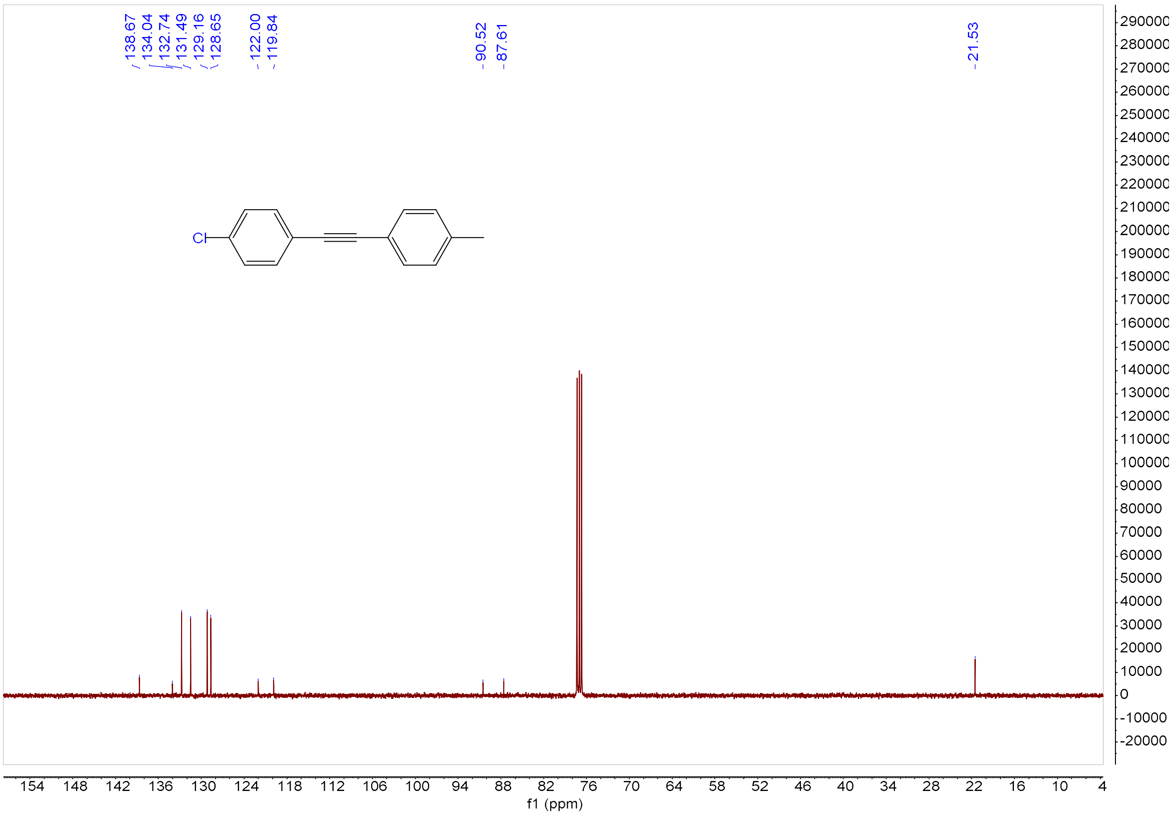

^{1}H NMR spectrum of compound **8da** (400 MHz, $CDCl_3$)

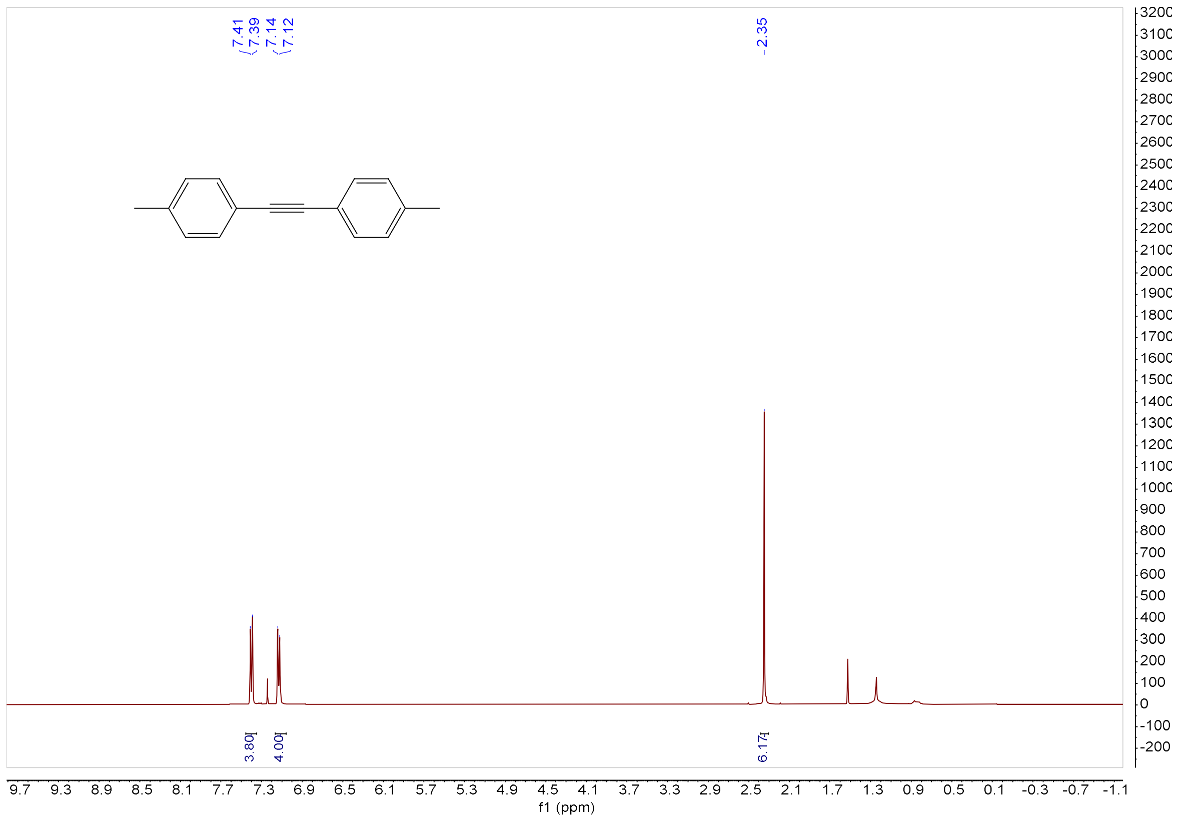

^{13}C NMR spectrum of compound **8da** (101 MHz, $CDCl_3$)

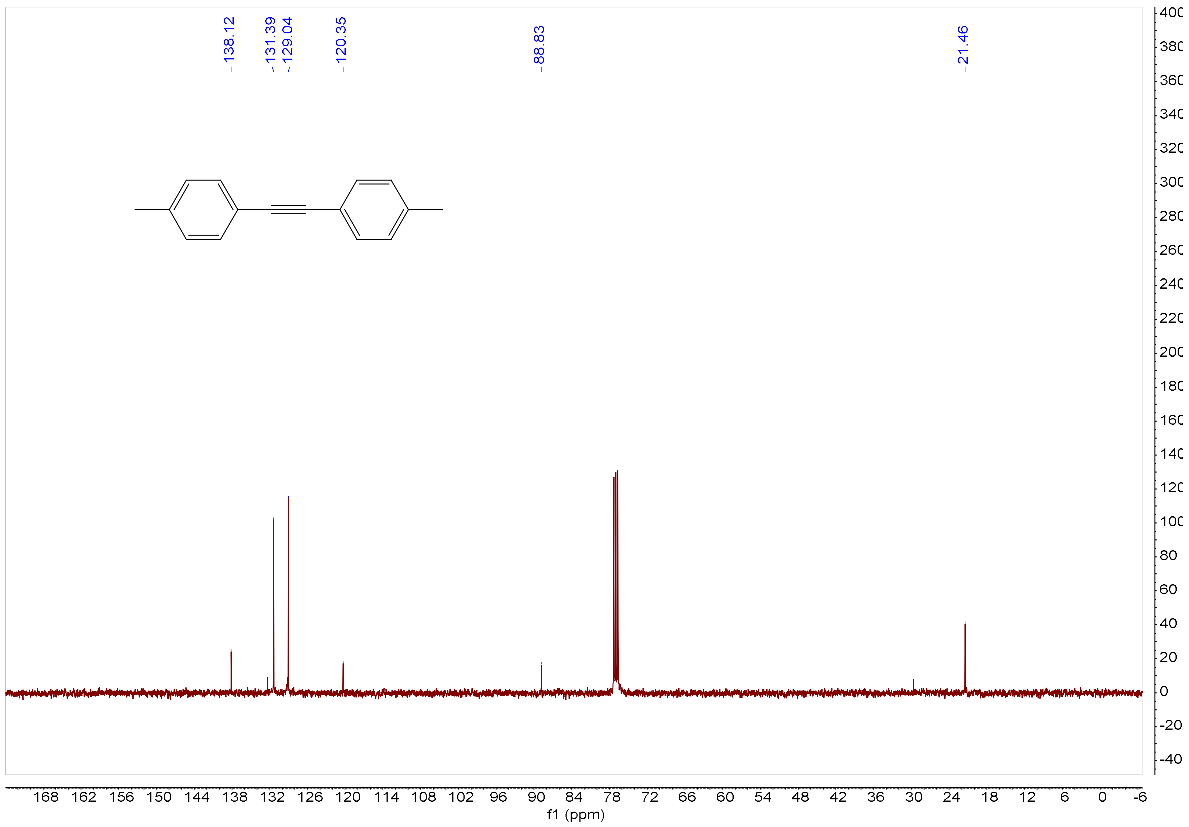

^{1}H NMR spectrum of compound **8fa** (600 MHz, $CDCl_3$)

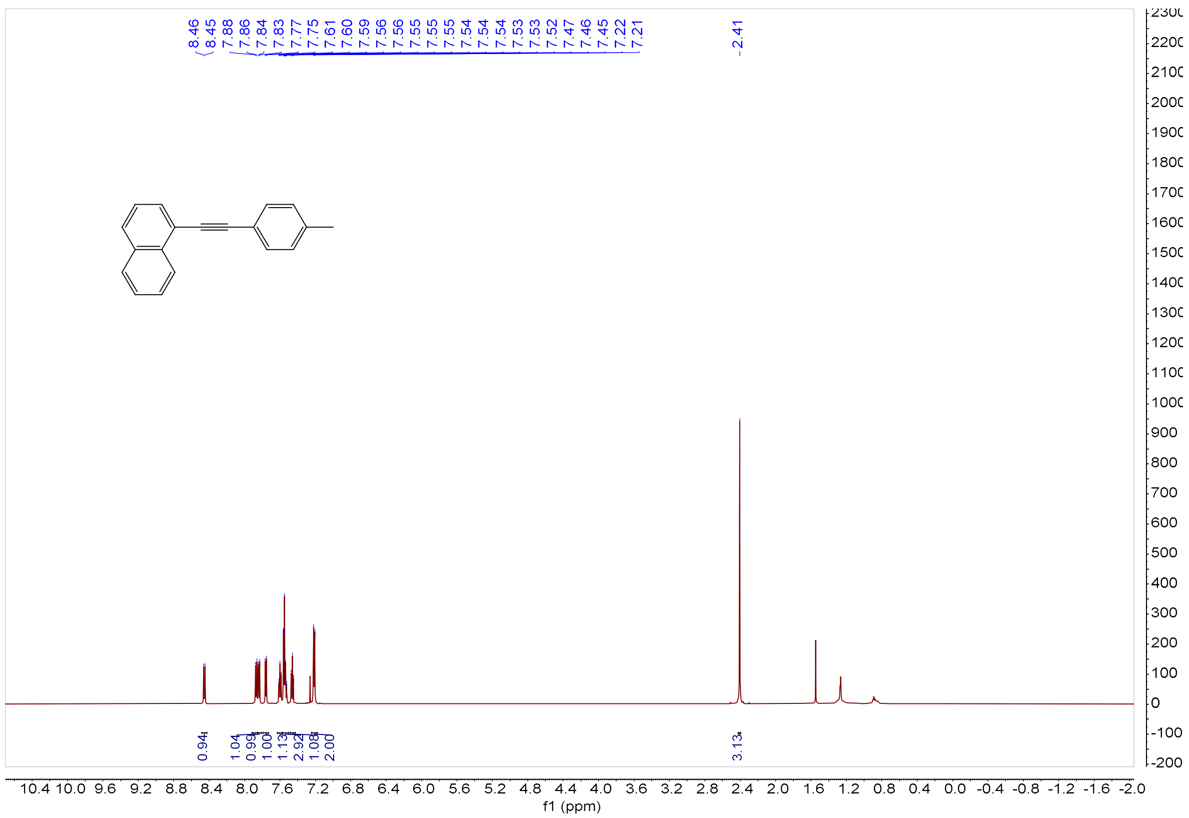

^{13}C NMR spectrum of compound **8fa** (151 MHz, $CDCl_3$)

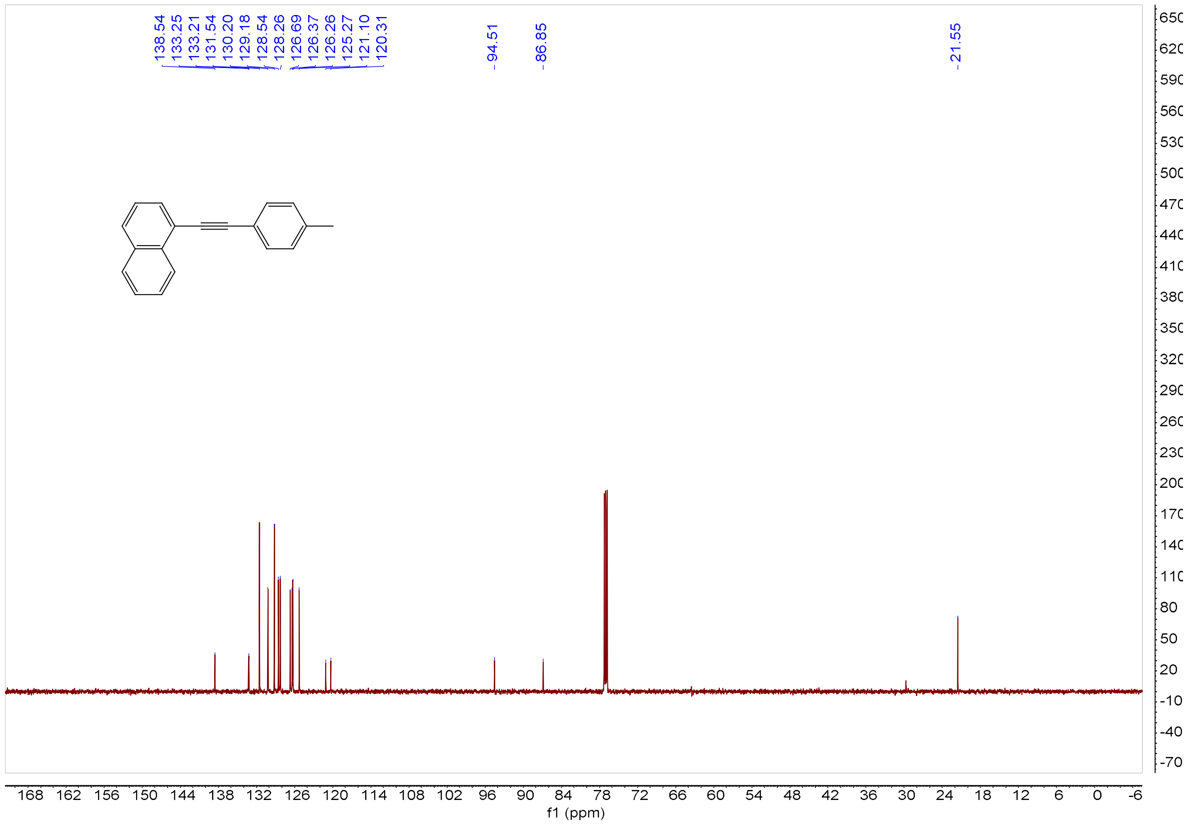

^{1}H NMR spectrum of compound **8ga** (600 MHz, $CDCl_3$)

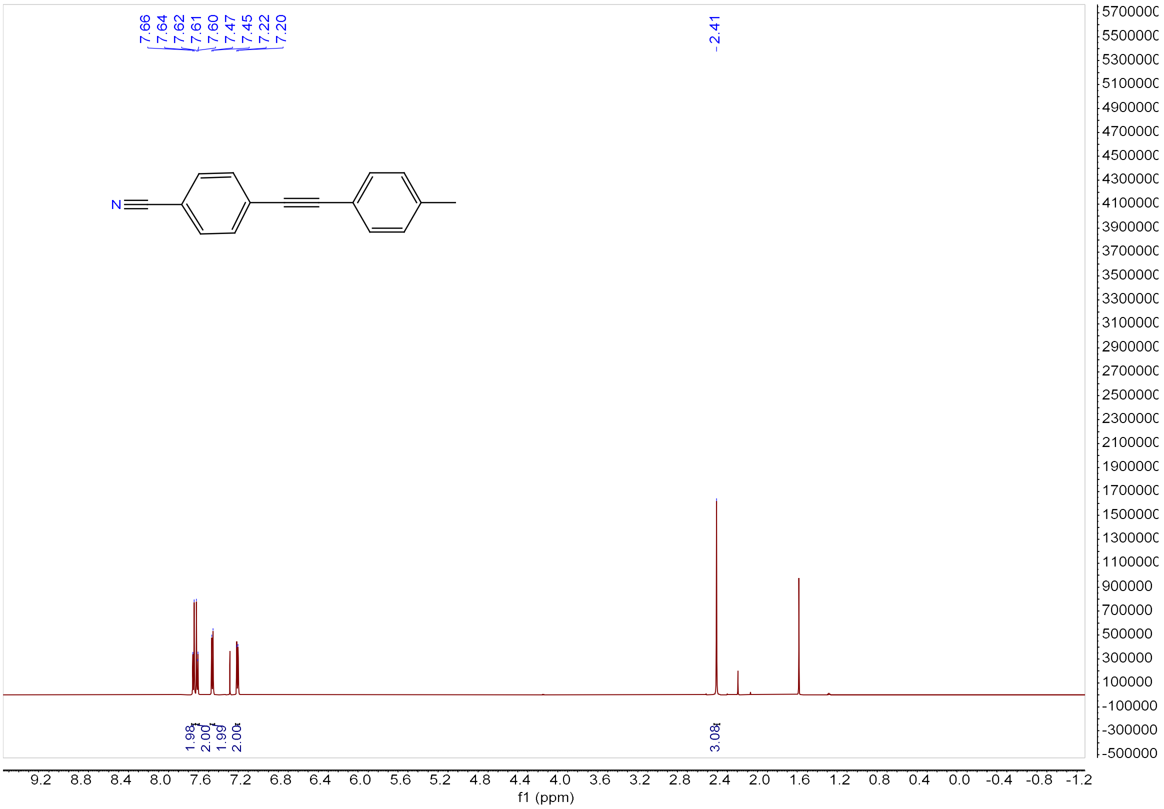

^{13}C NMR spectrum of compound **8ga** (151 MHz, $CDCl_3$)

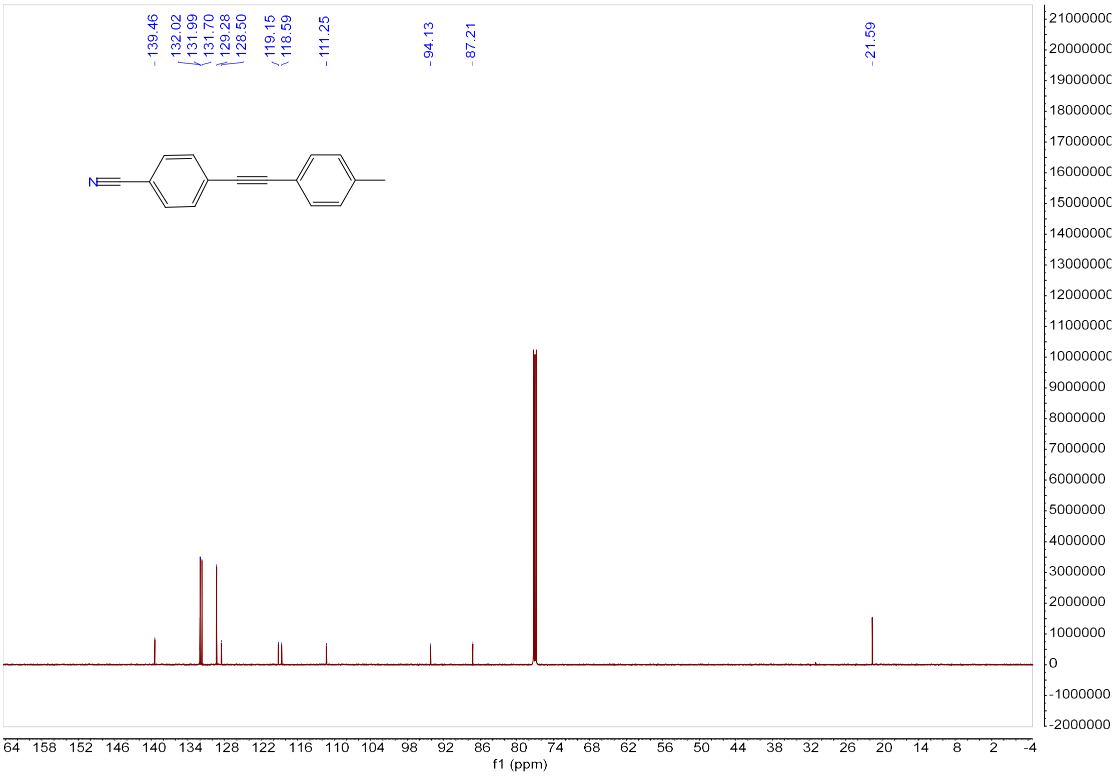

^{1}H NMR spectrum of compound **8bb** (600 MHz, $CDCl_3$)

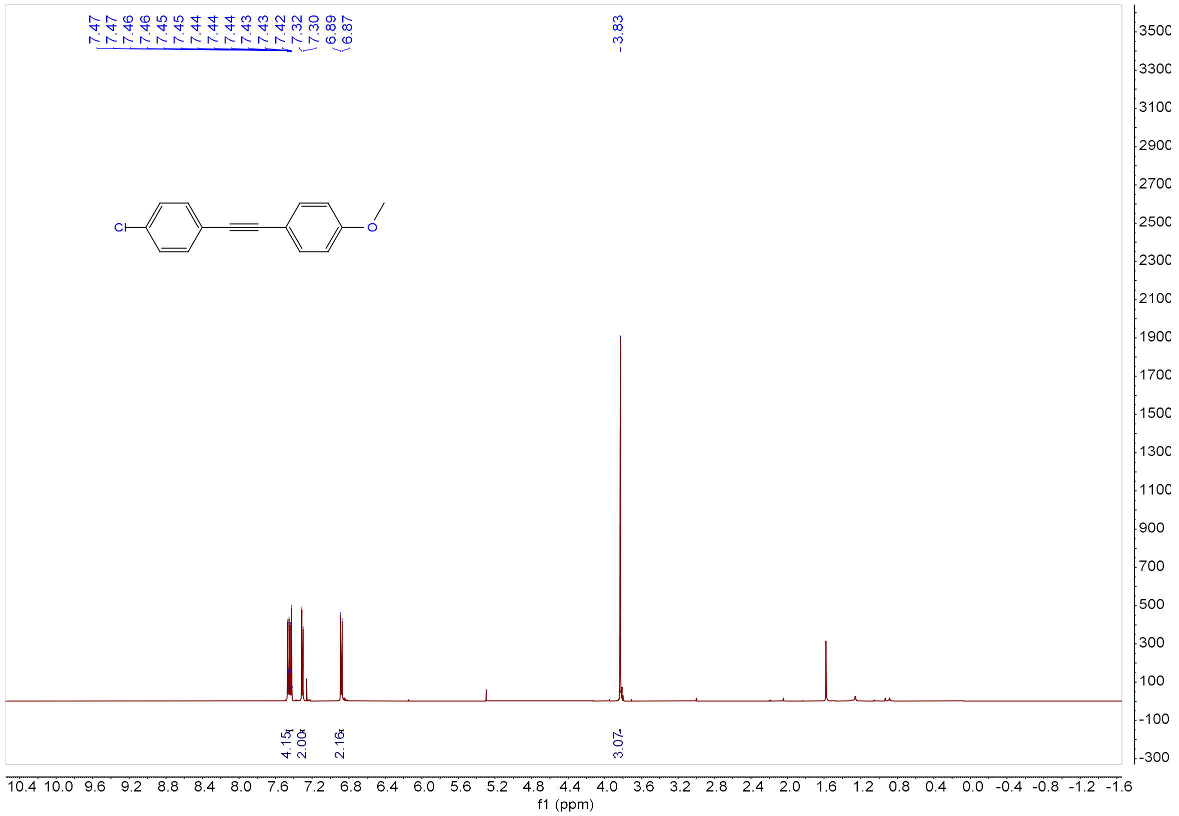

^{13}C NMR spectrum of compound **8bb** (151 MHz, $CDCl_3$)

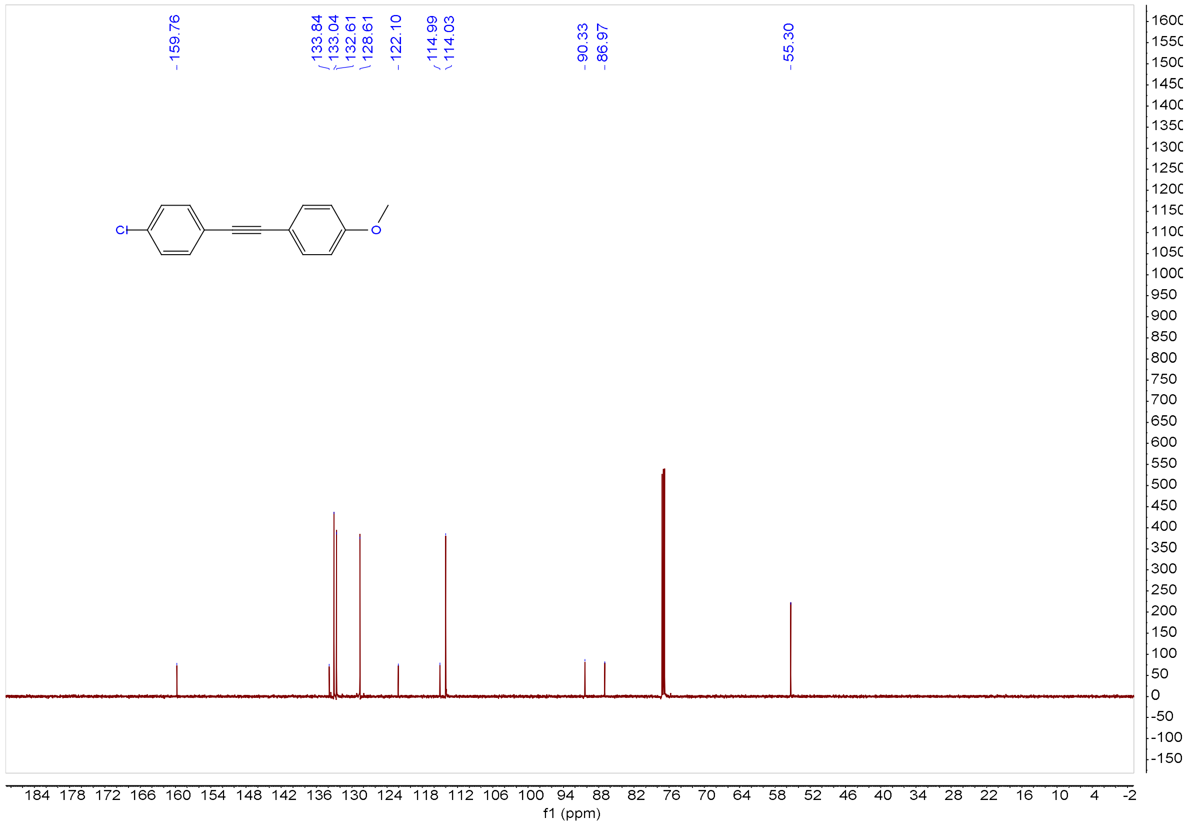

^{1}H NMR spectrum of compound **8bc** (400 MHz, $CDCl_3$)

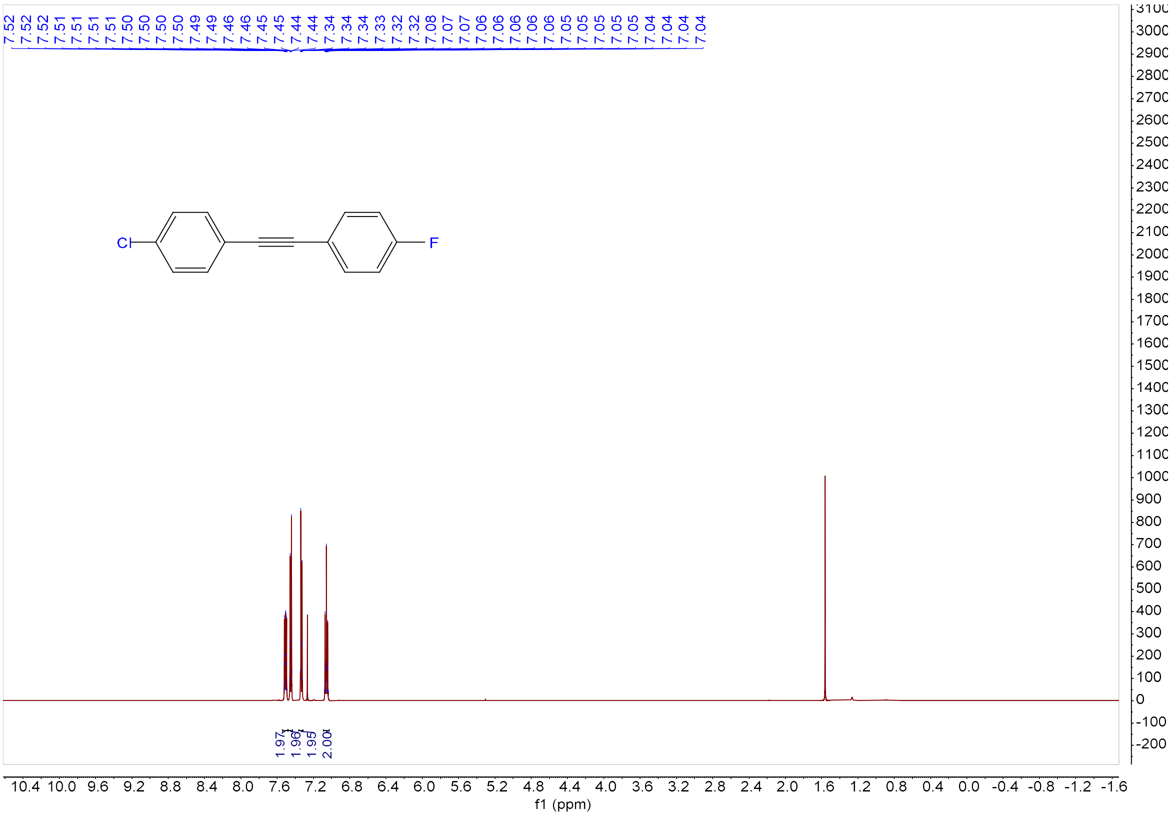

^{13}C NMR spectrum of compound **8bc** (151 MHz, $CDCl_3$)

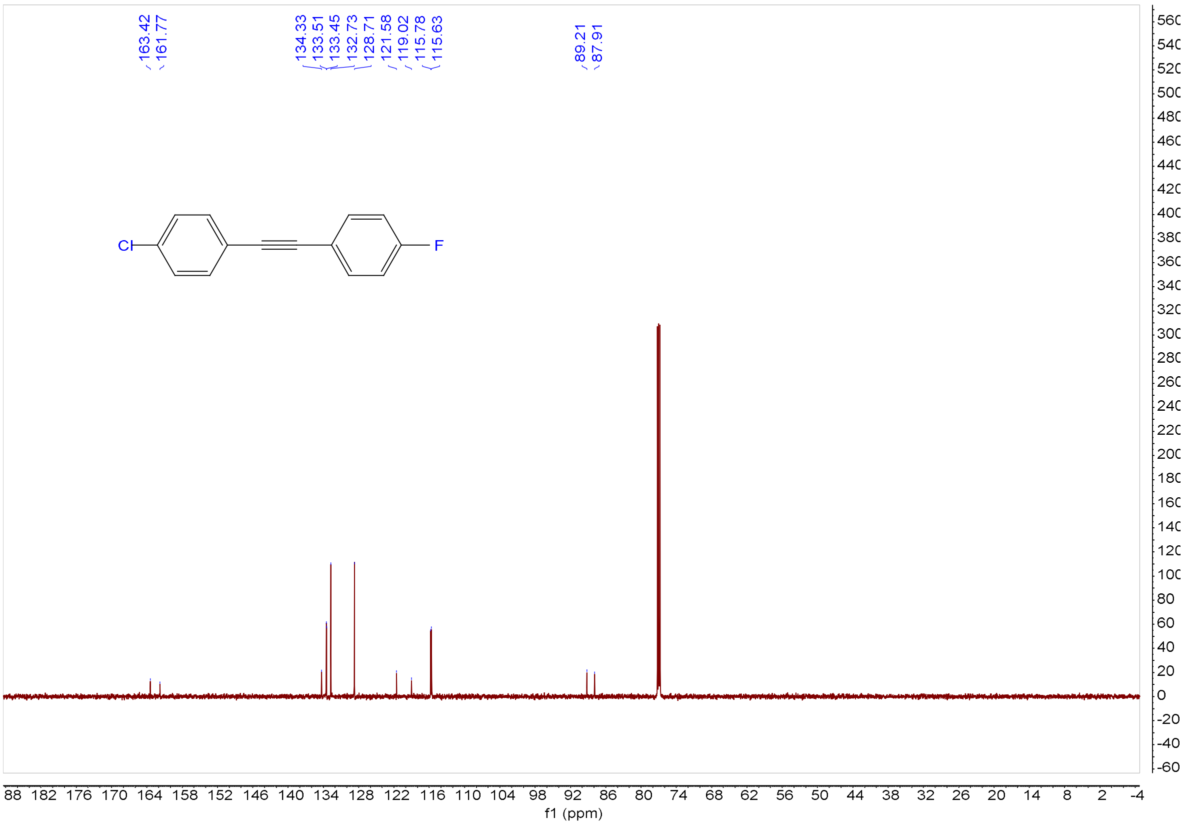

^{19}F NMR spectrum of compound **8bc** (376 MHz, $CDCl_3$)

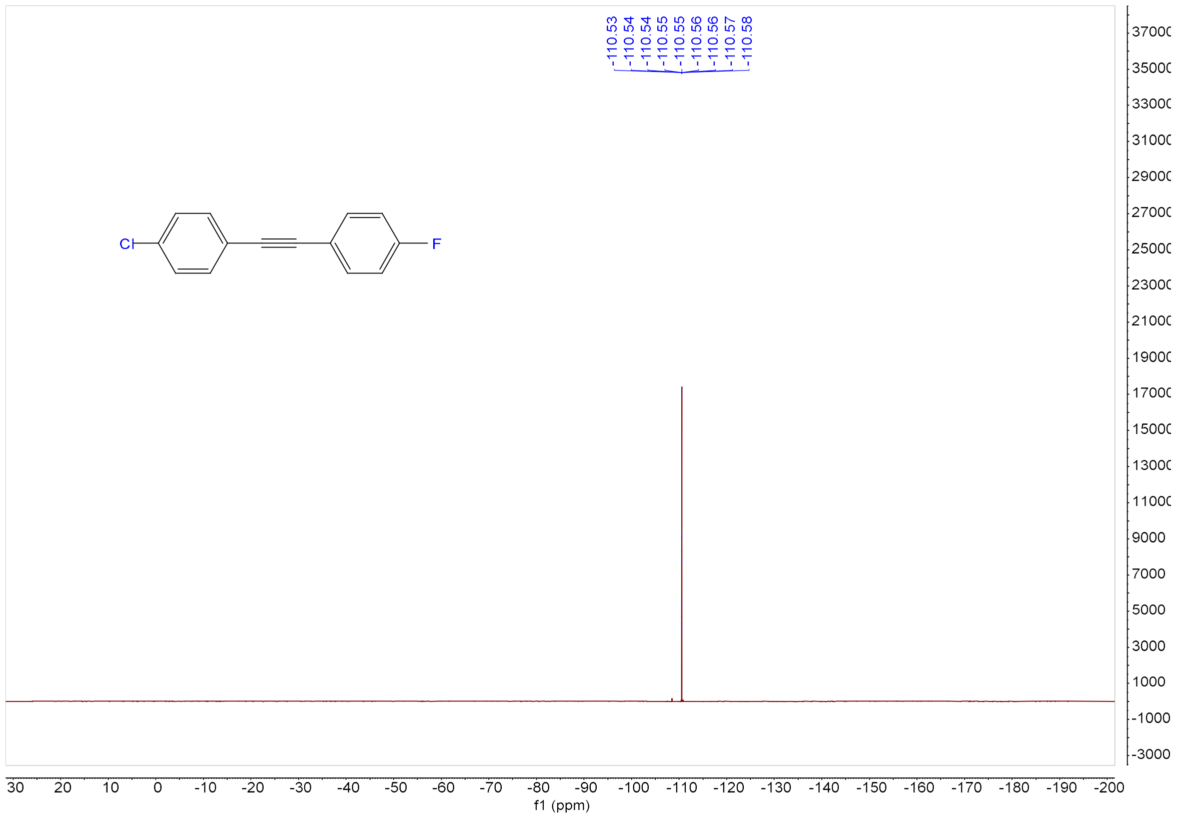

^{1}H NMR spectrum of compound **8bd** (400 MHz, $CDCl_3$)

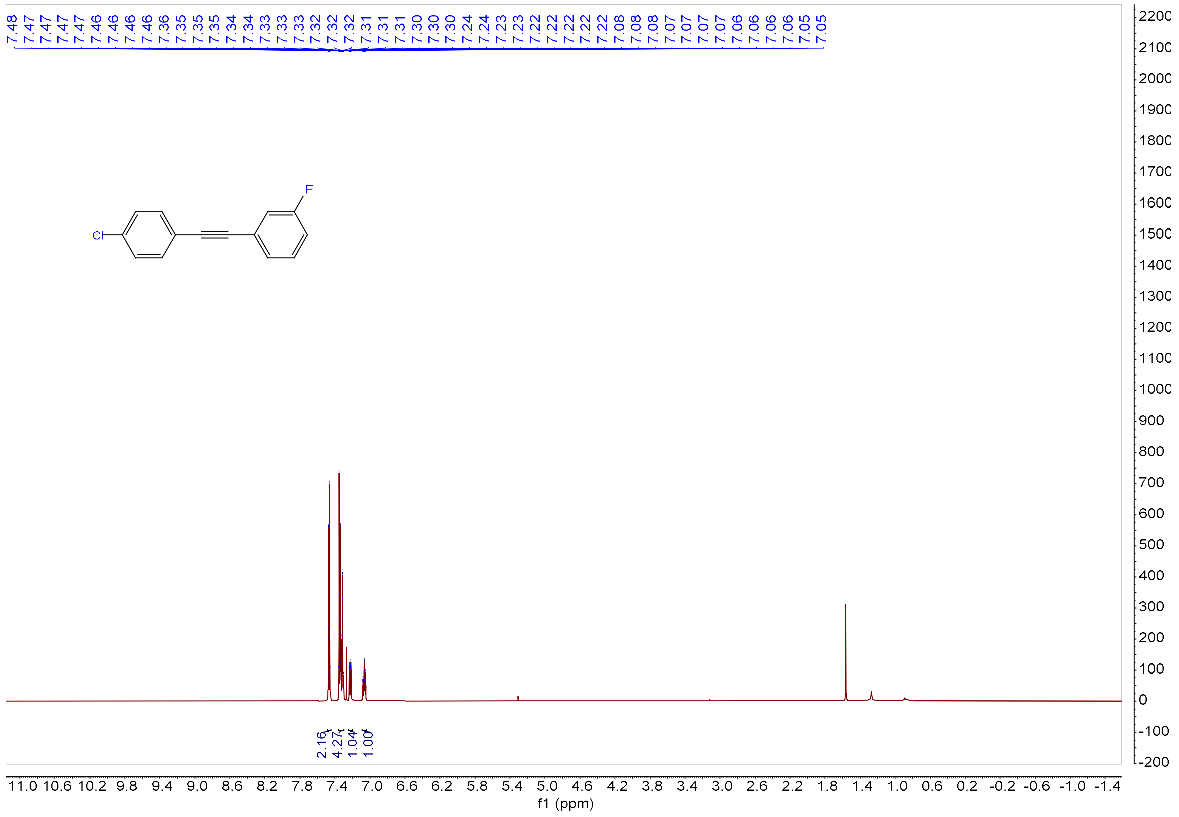

^{13}C NMR spectrum of compound **8bd** (151 MHz, $CDCl_3$)

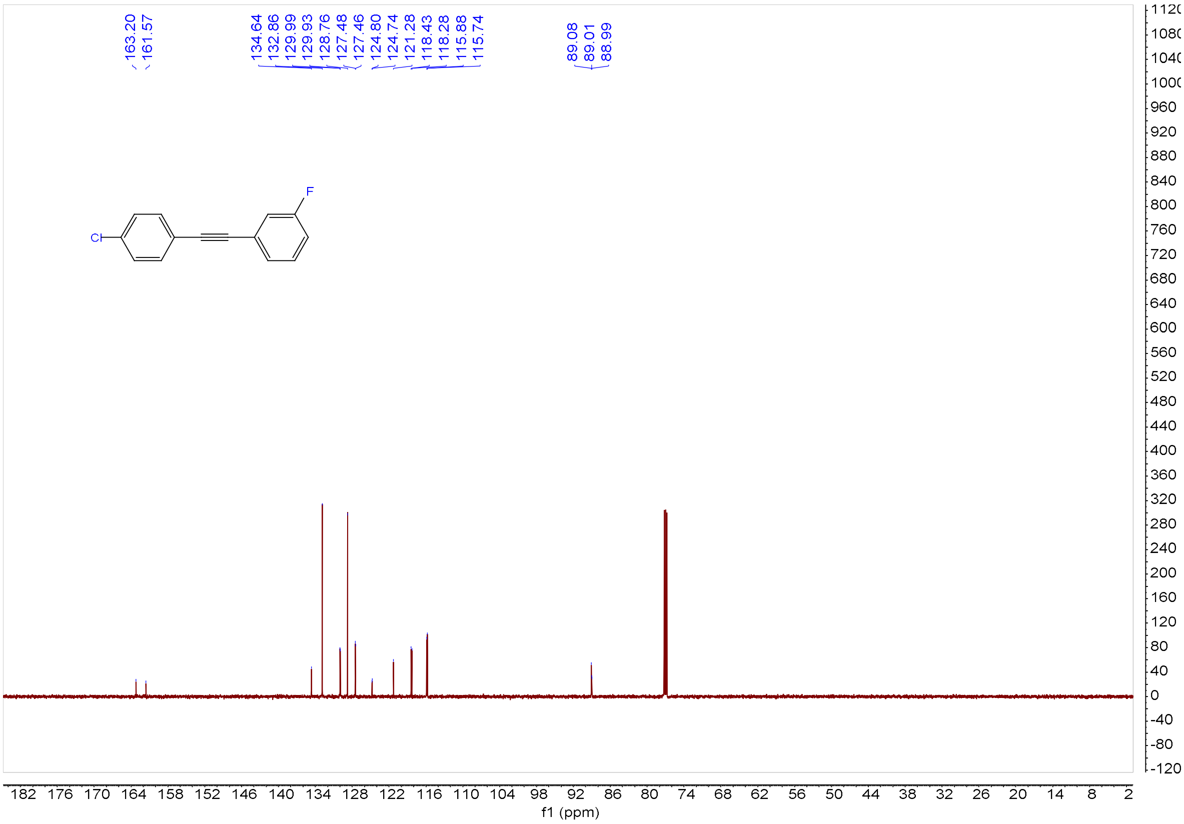

^{19}F NMR spectrum of compound **8bd** (376 MHz, $CDCl_3$)

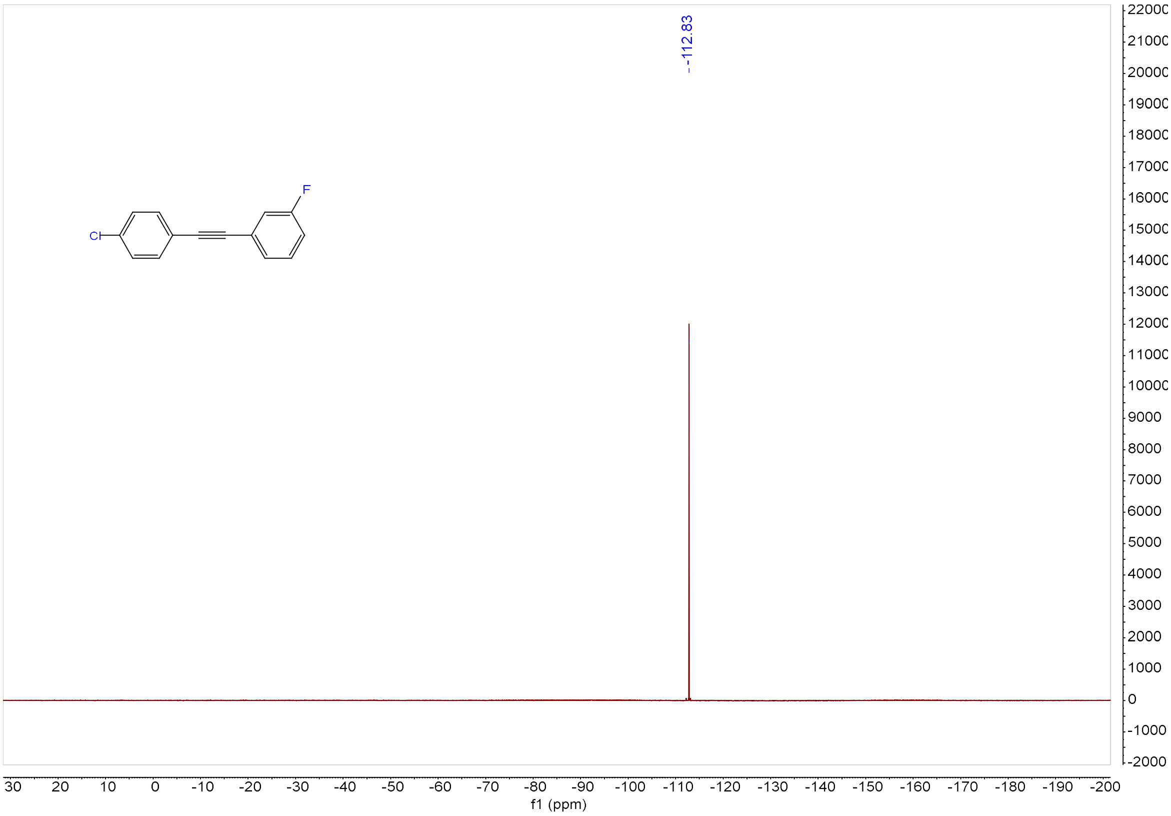

^{1}H NMR spectrum of compound **8be** (400 MHz, $CDCl_3$)

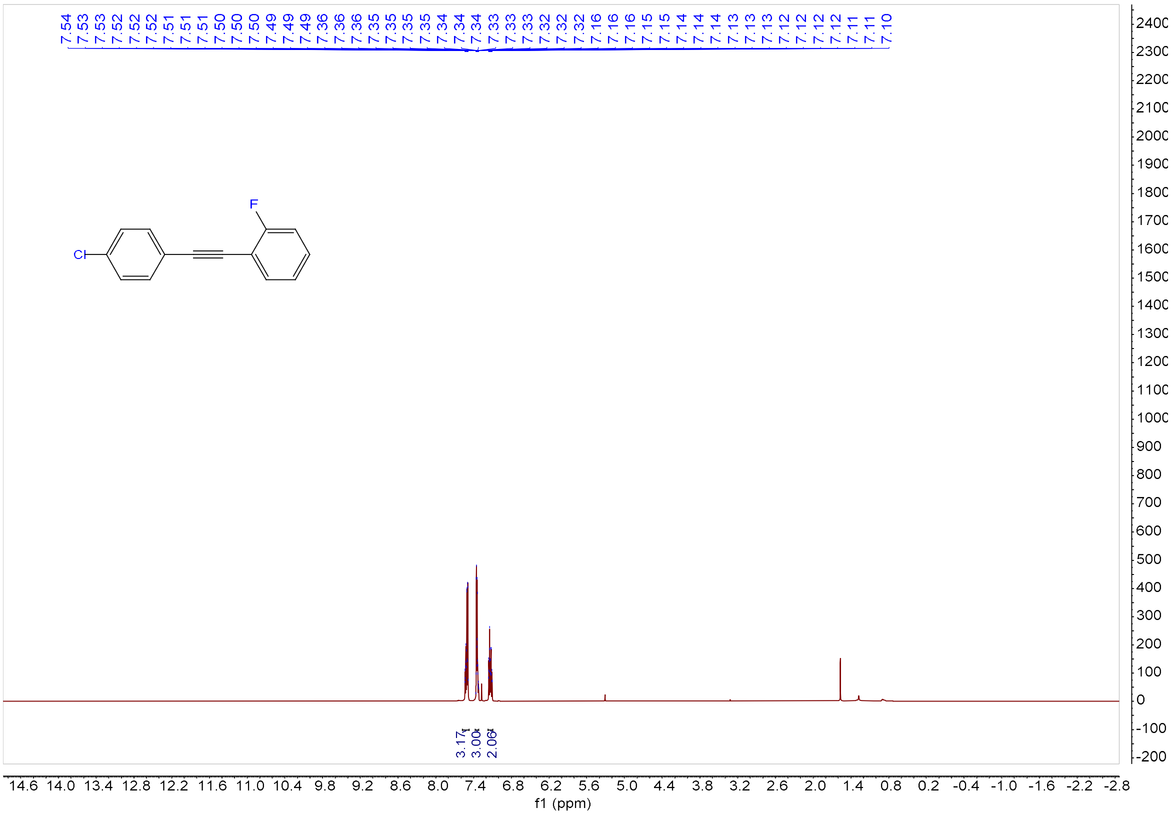

^{13}C NMR spectrum of compound **8be** (151 MHz, $CDCl_3$)

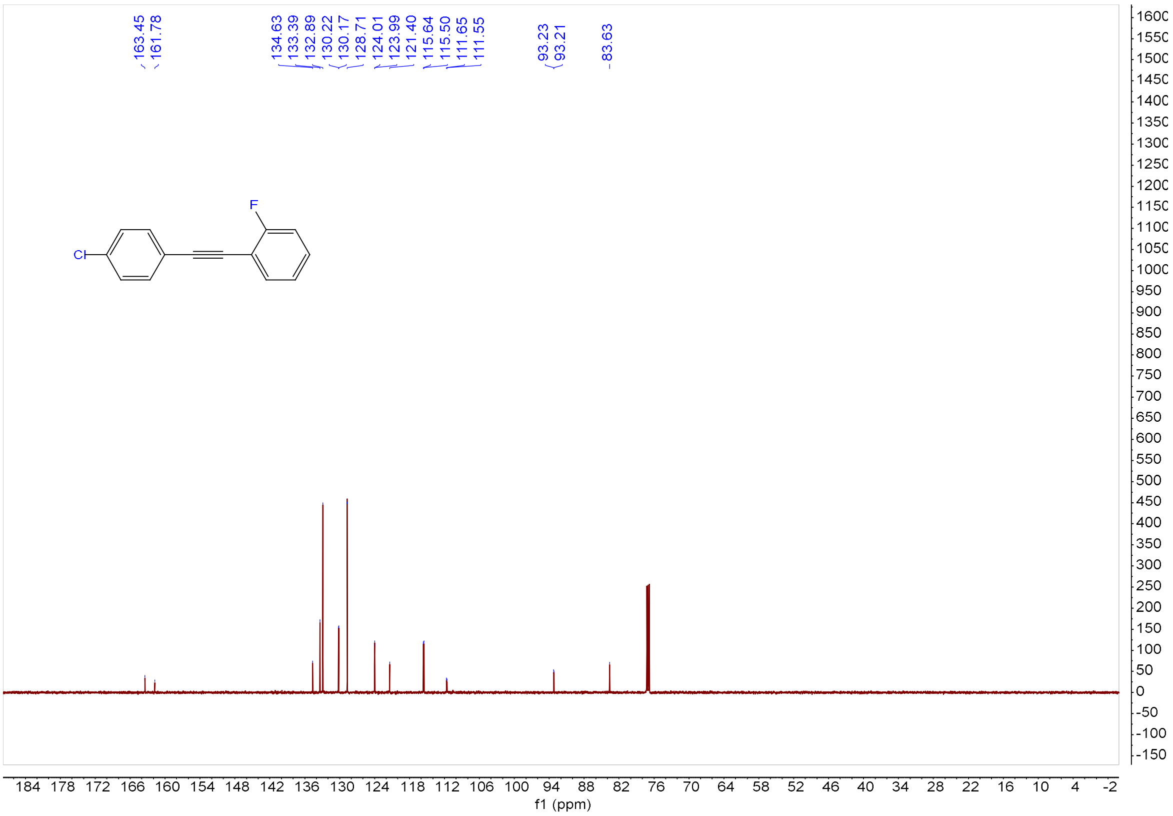

^{19}F NMR spectrum of compound **8be** (376 MHz, $CDCl_3$)

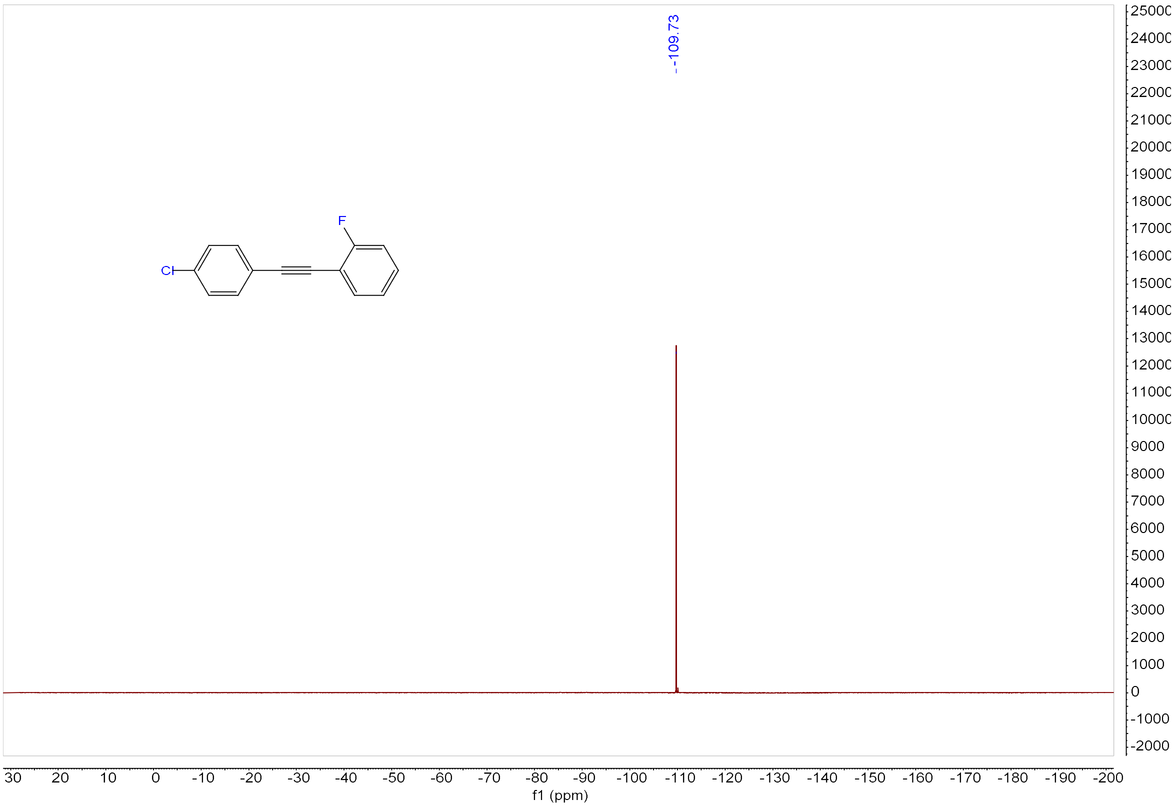

^{1}H NMR spectrum of compound **8bf** (400 MHz, $CDCl_3$)

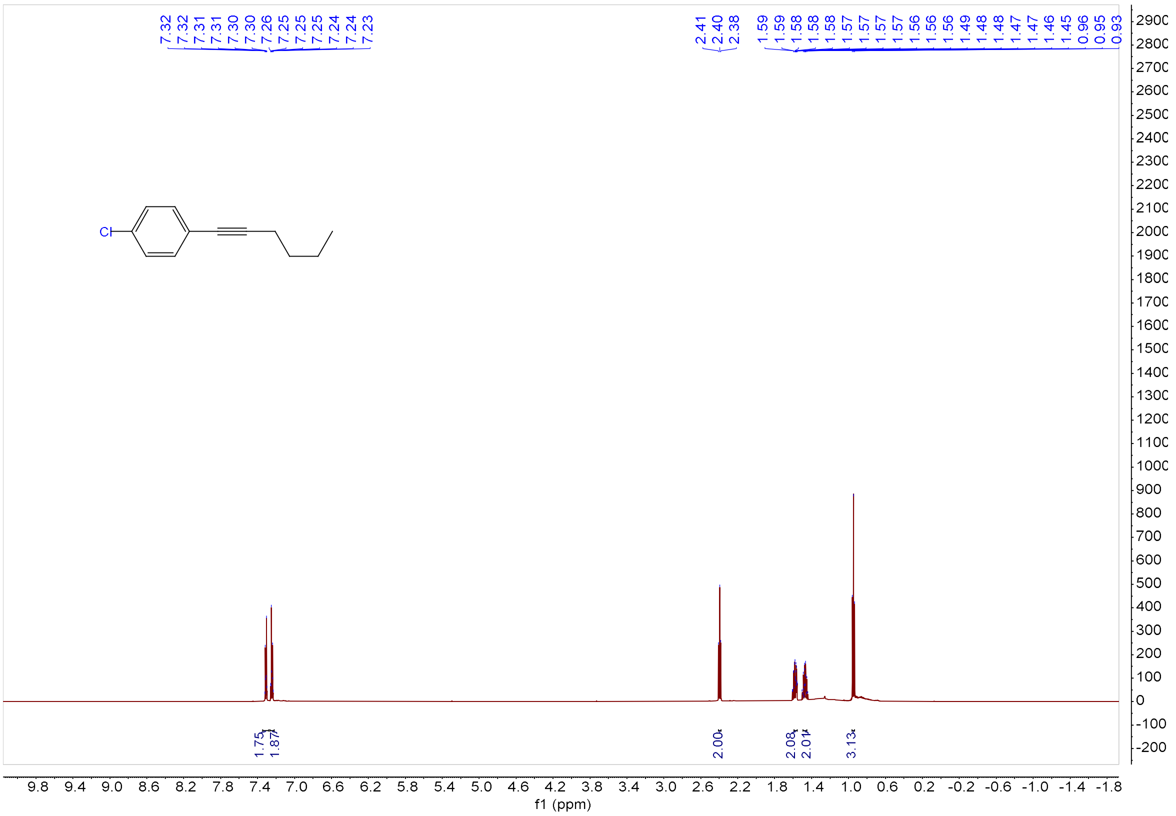

^{13}C NMR spectrum of compound **8bf** (151 MHz, $CDCl_3$)

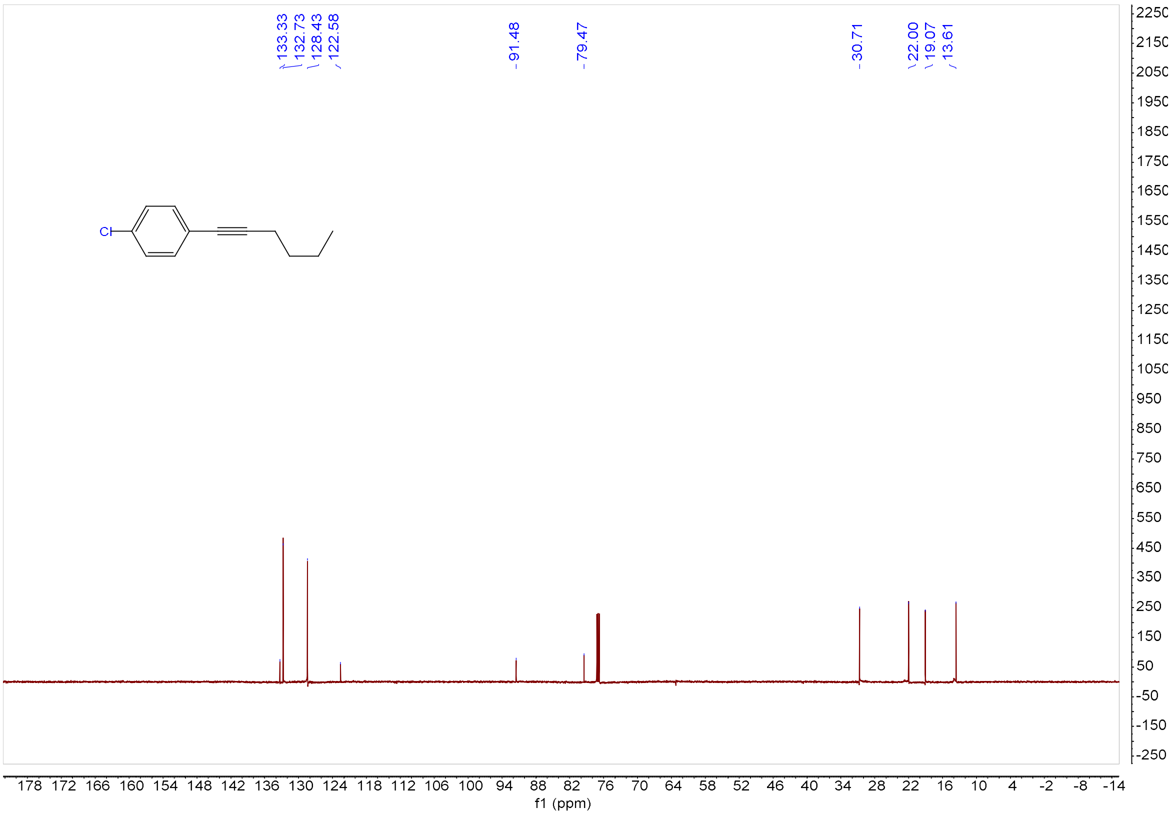

^{1}H NMR spectrum of compound **10aa** (400 MHz, $CDCl_3$)

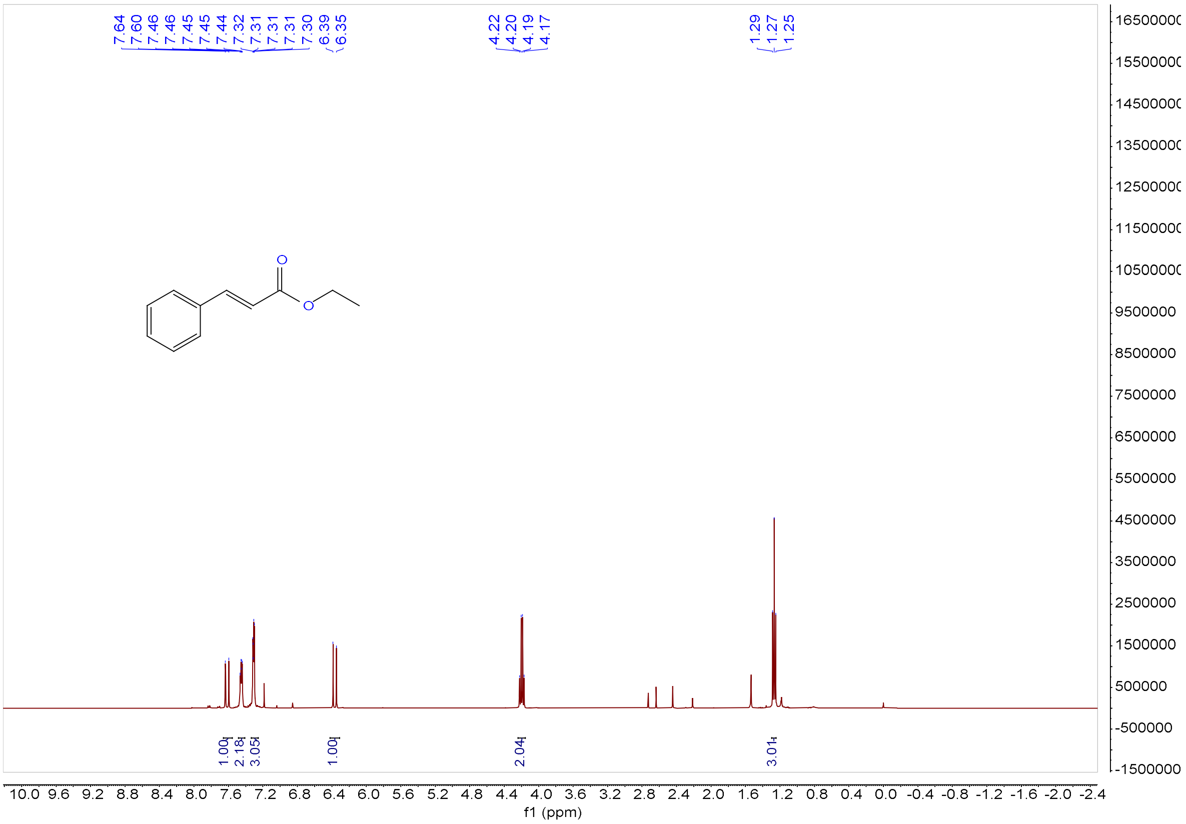

^{13}C NMR spectrum of compound **10aa** (101 MHz, $CDCl_3$)

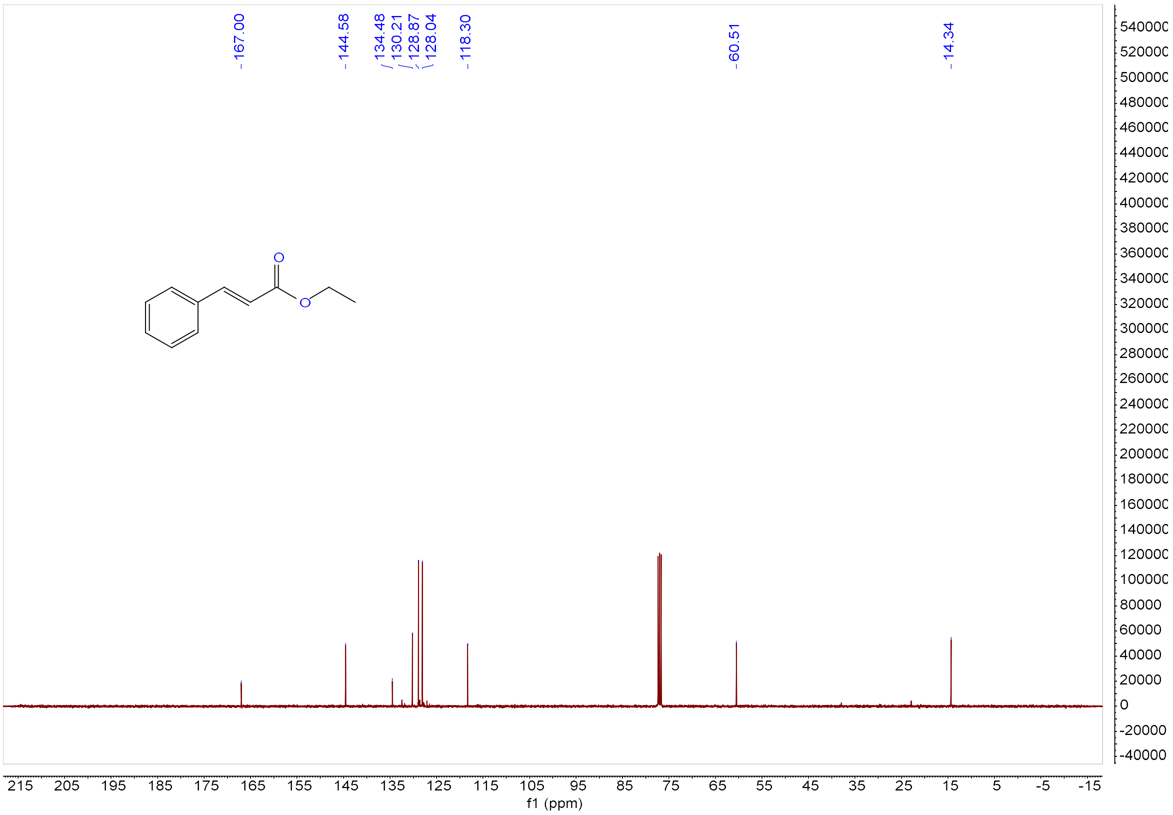

^{1}H NMR spectrum of compound **10ba** (400 MHz, $CDCl_3$)

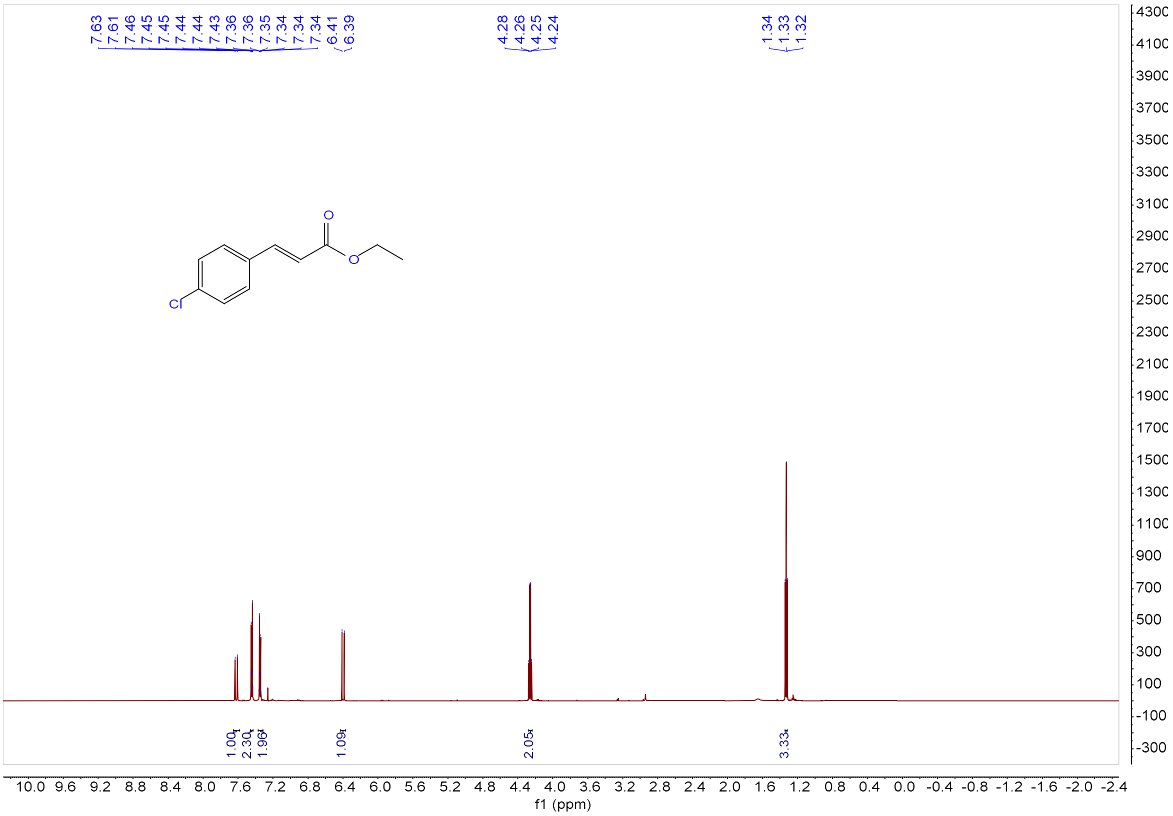

^{13}C NMR spectrum of compound **10ba** (151 MHz, $CDCl_3$)

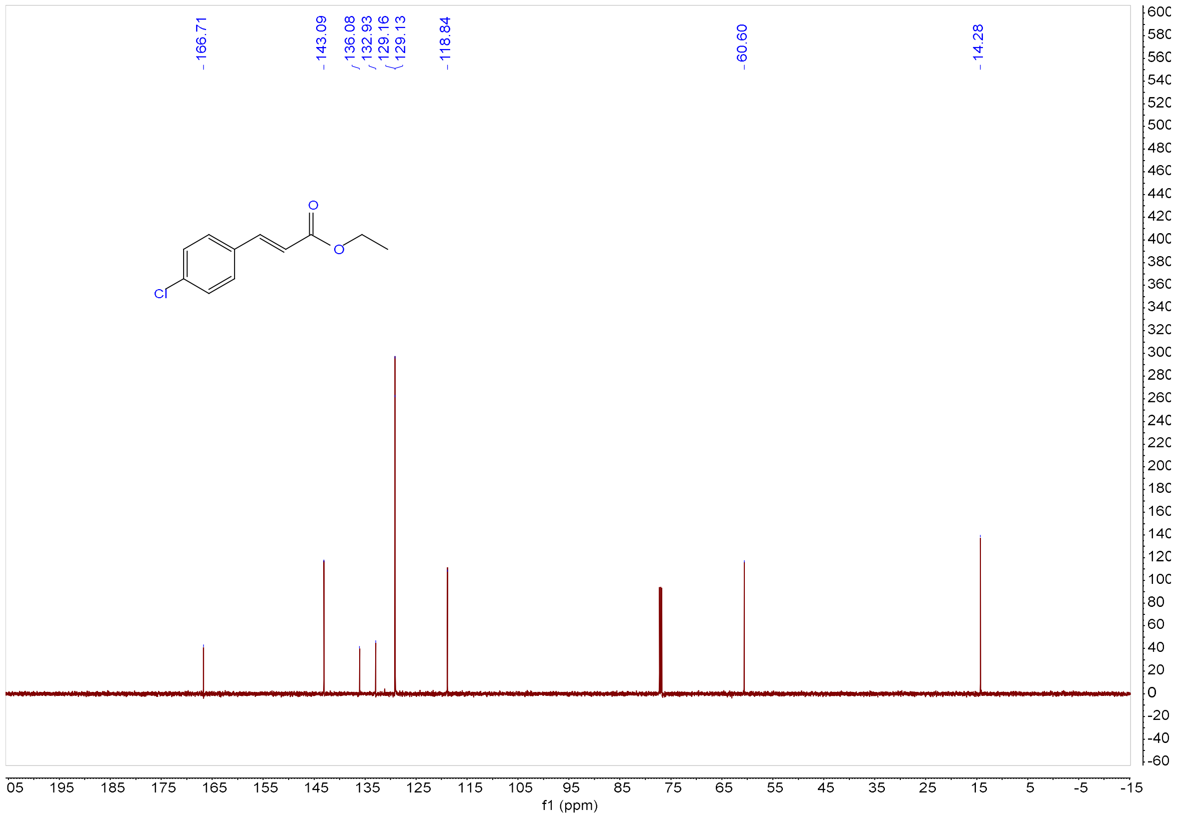

^1H NMR spectrum of compound **10da** (600 MHz, $CDCl_3$)

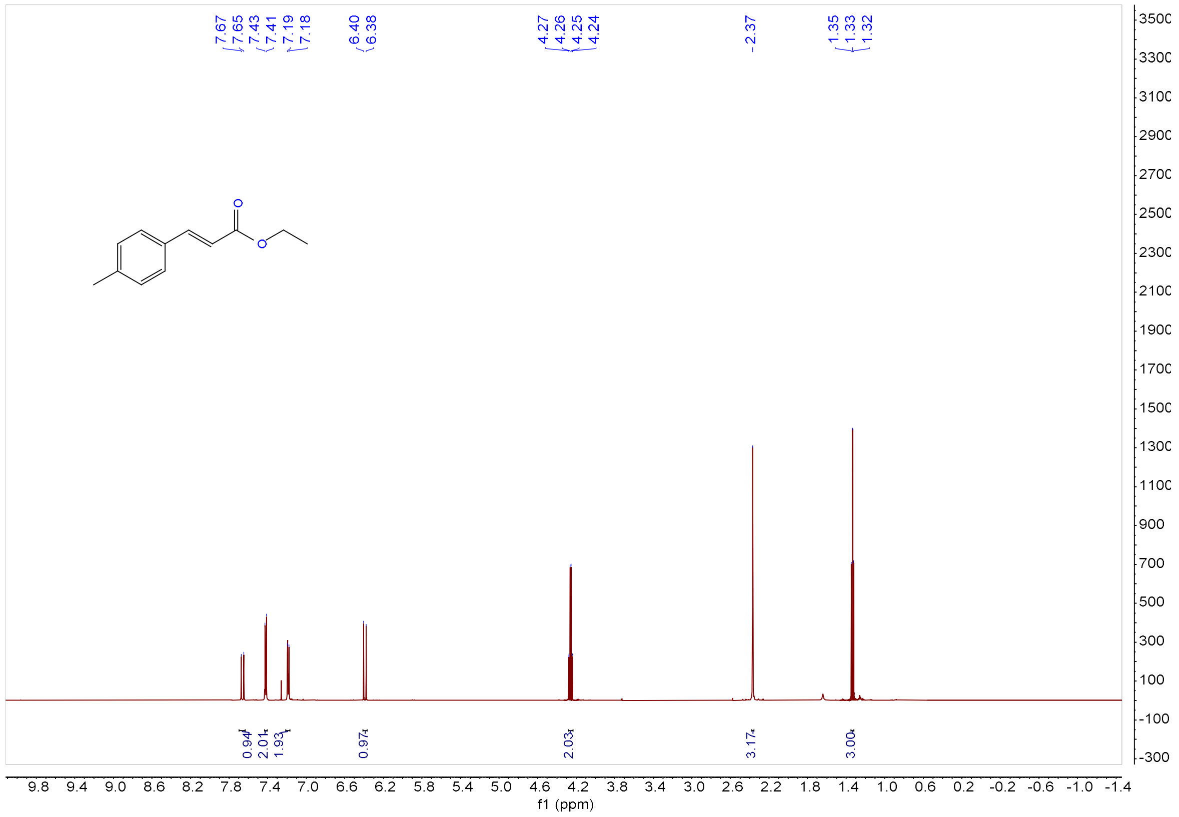

^{13}C NMR spectrum of compound **10da** (151 MHz, $CDCl_3$)

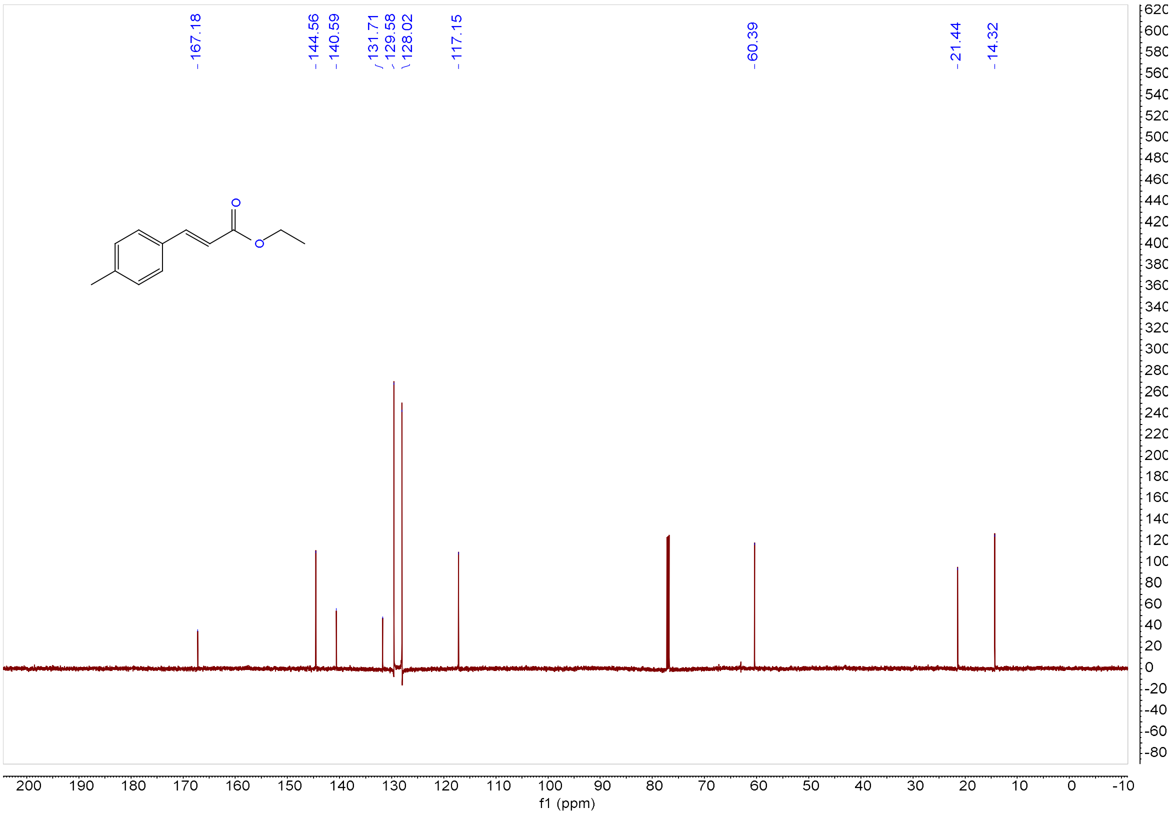

^{1}H NMR spectrum of compound **10ab** (400 MHz, $CDCl_3$)

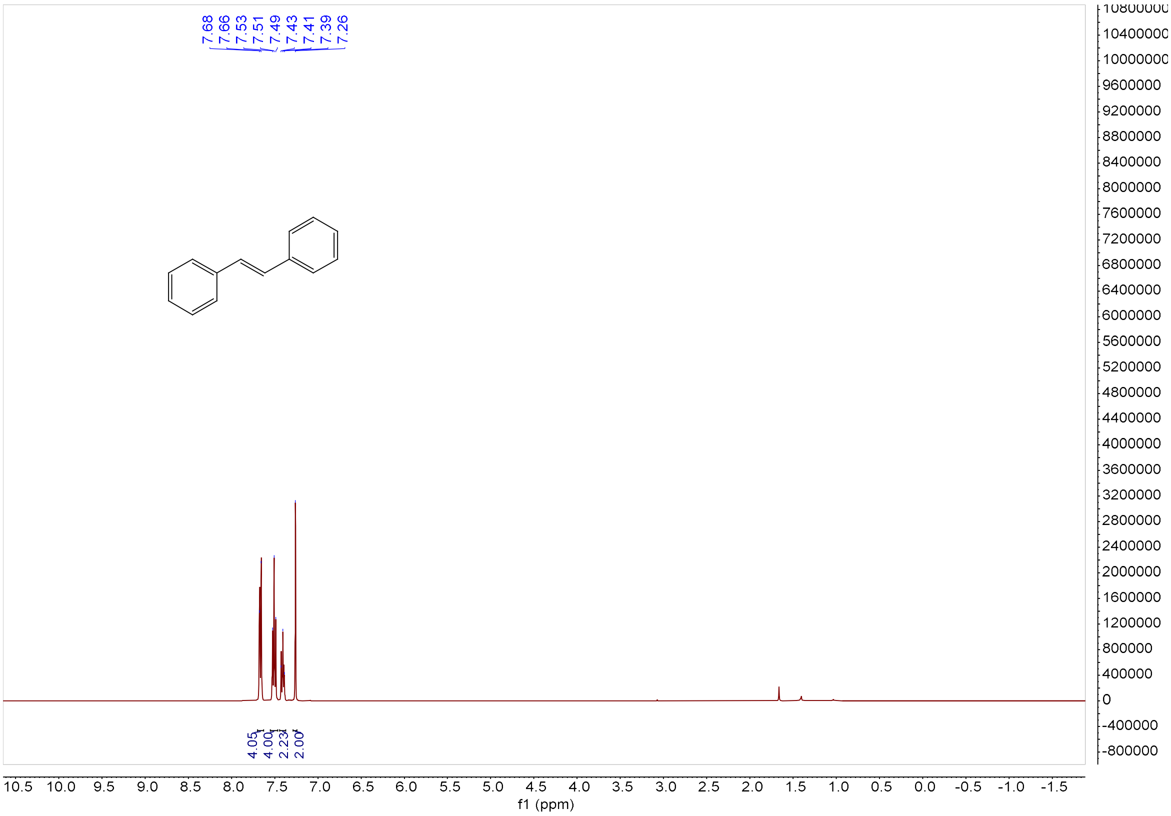

^{13}C NMR spectrum of compound **10ab** (101 MHz, $CDCl_3$)

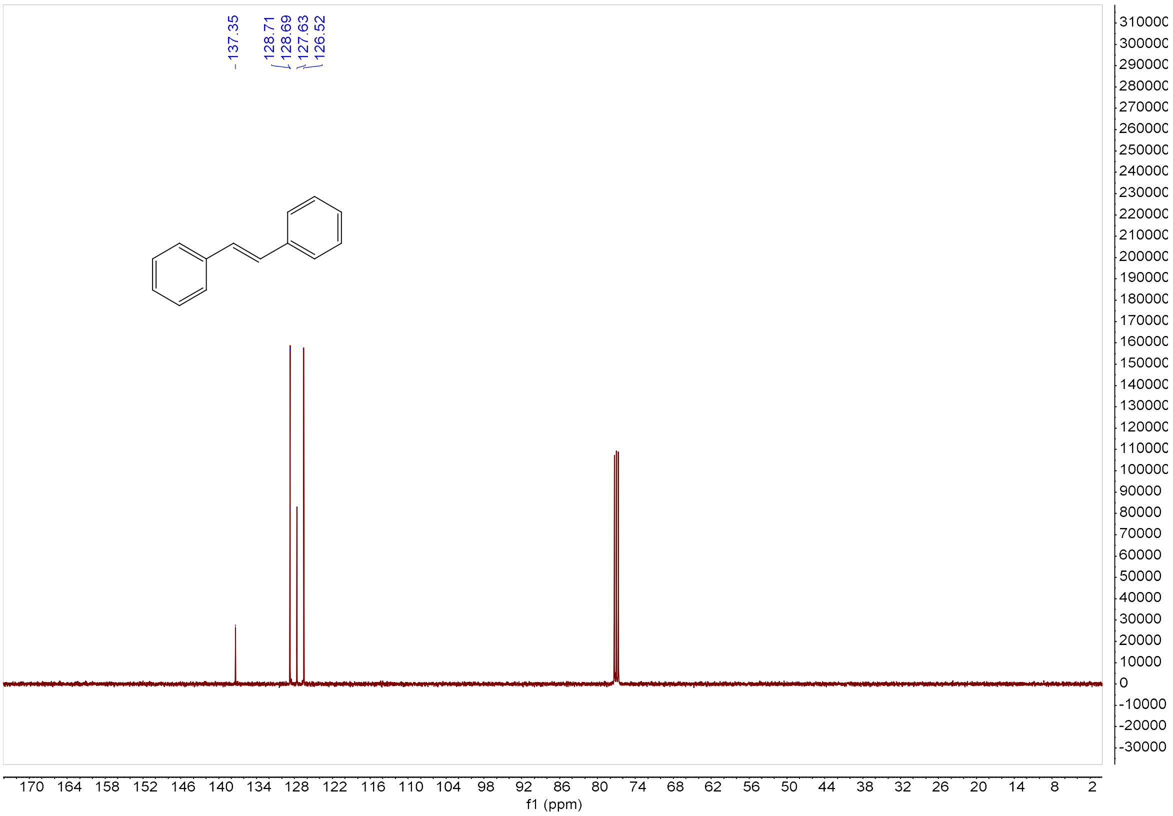

^{1}H NMR spectrum of compound **10ac** (600 MHz, $CDCl_3$)

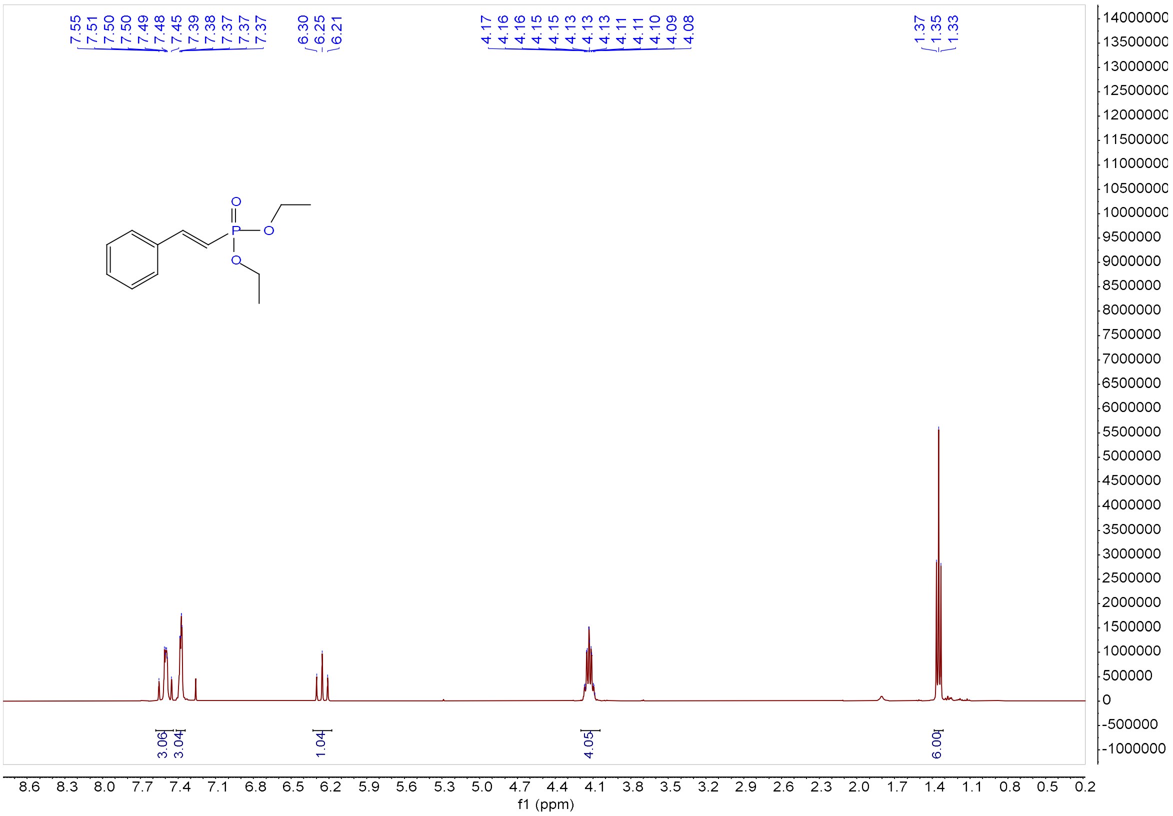

^{13}C NMR spectrum of compound **10ac** (151 MHz, $CDCl_3$)

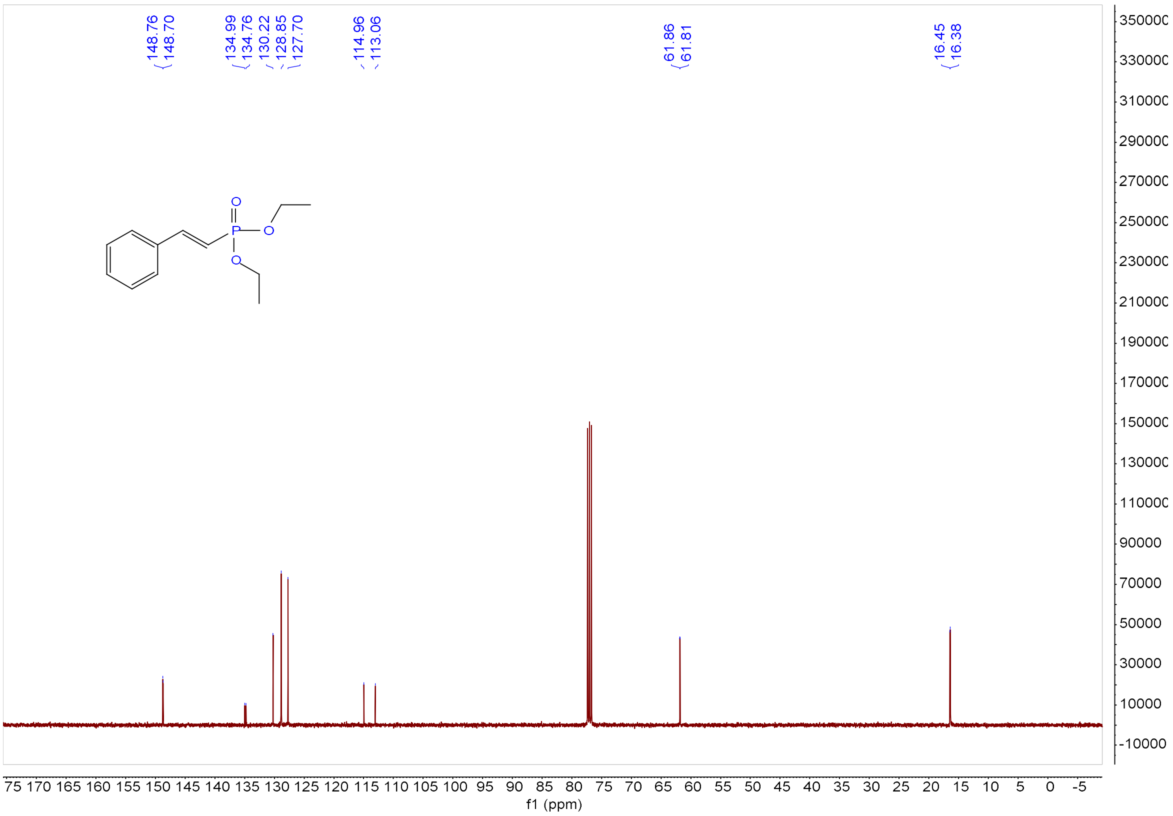

^{1}H NMR spectrum of compound **12aa** (400 MHz, $CDCl_3$)

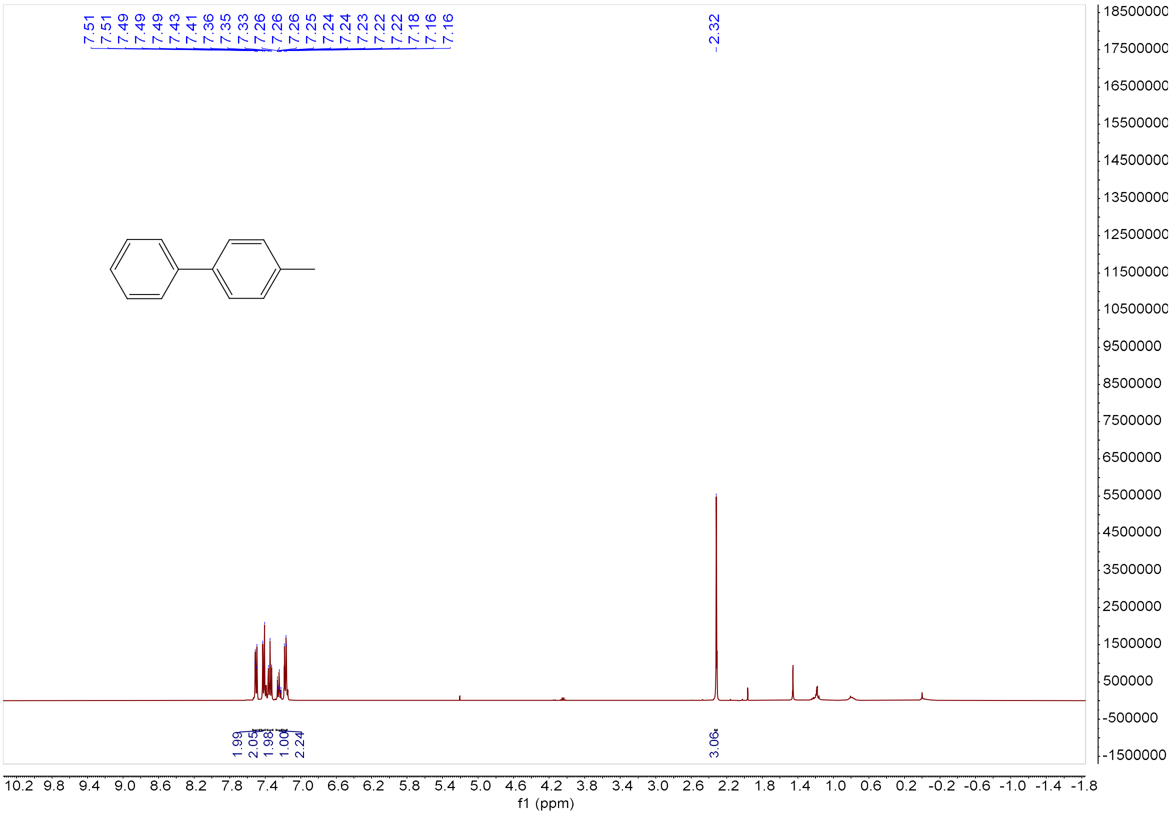

^{13}C NMR spectrum of compound **12aa** (101 MHz, $CDCl_3$)

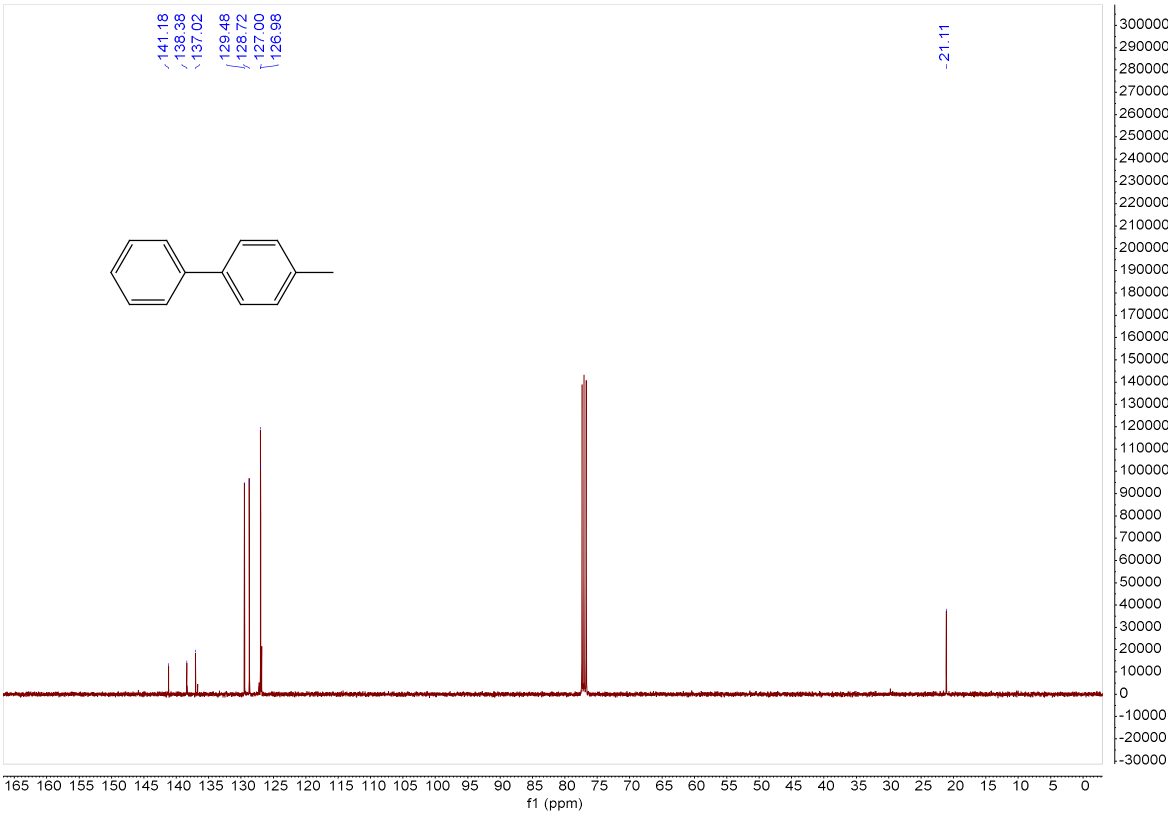

^{1}H NMR spectrum of compound **12ba** (400 MHz, $CDCl_3$)

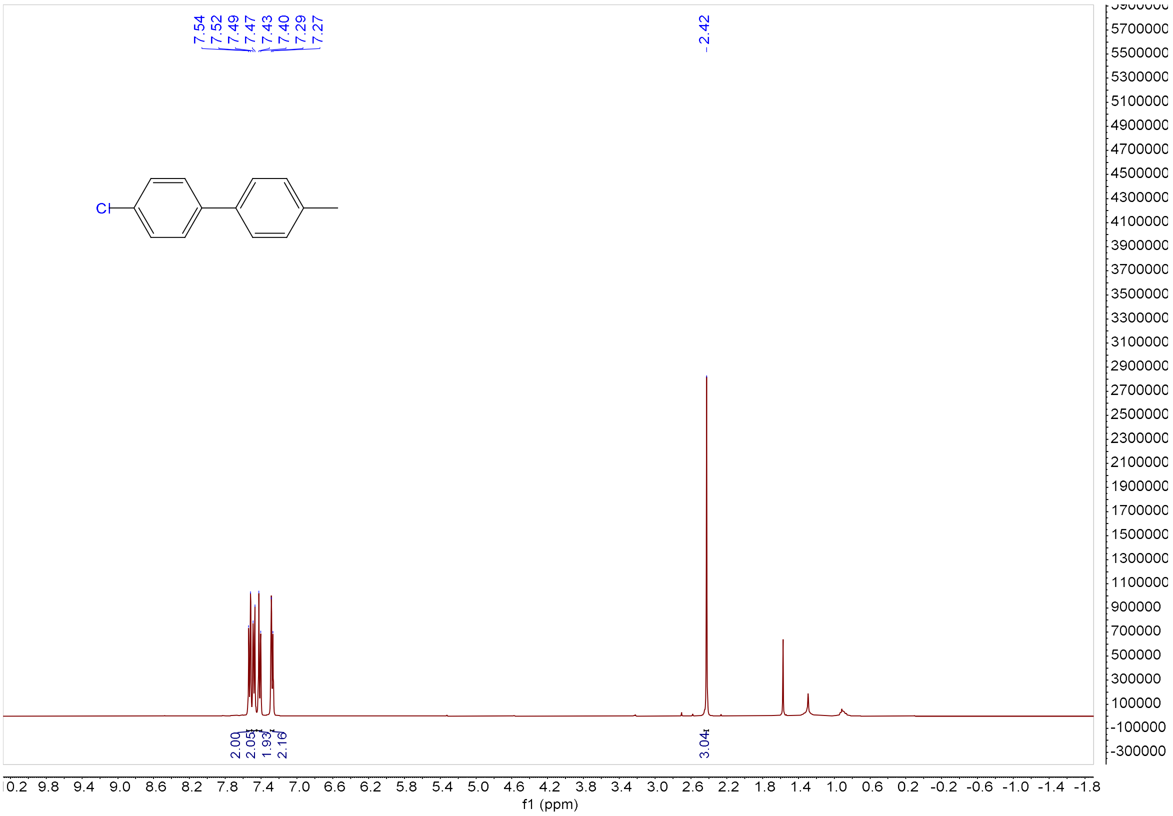

^{13}C NMR spectrum of compound **12ba** (101 MHz, CDCl$_3$)

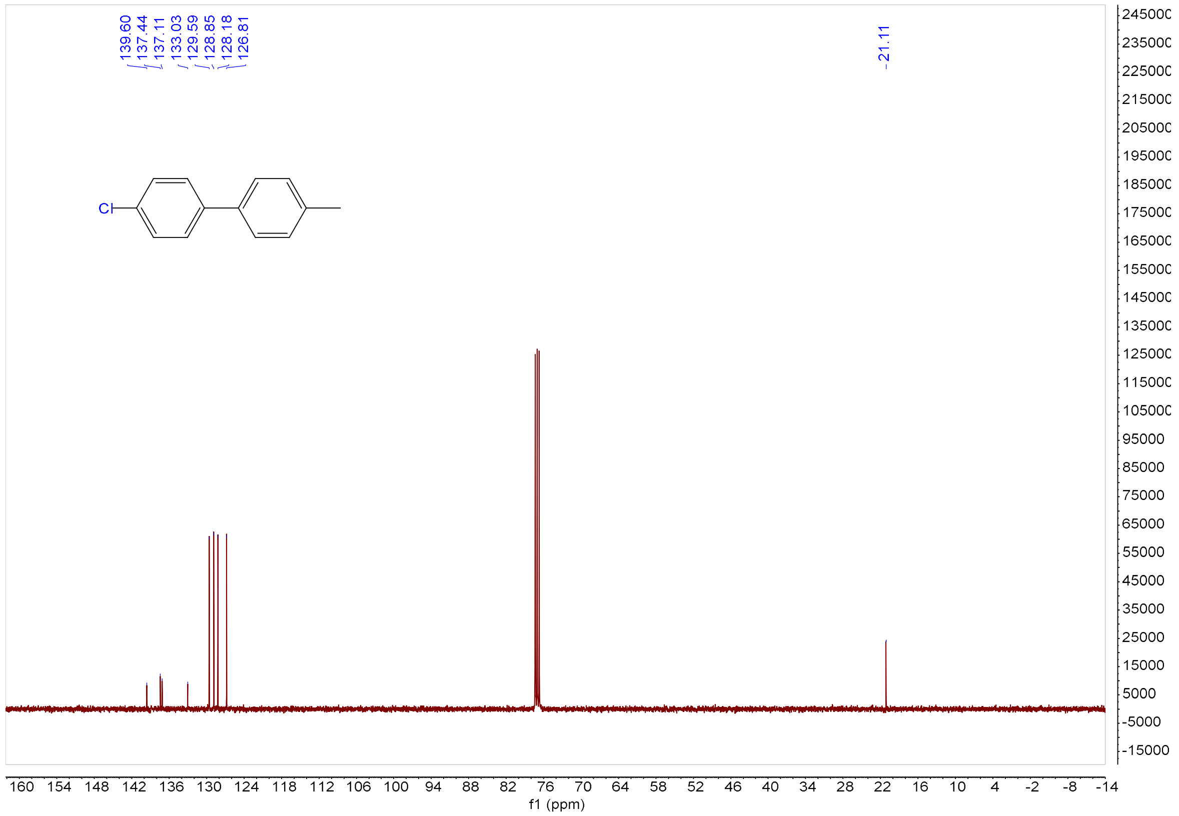

^{1}H NMR spectrum of compound **12da** (400 MHz, CDCl$_3$)

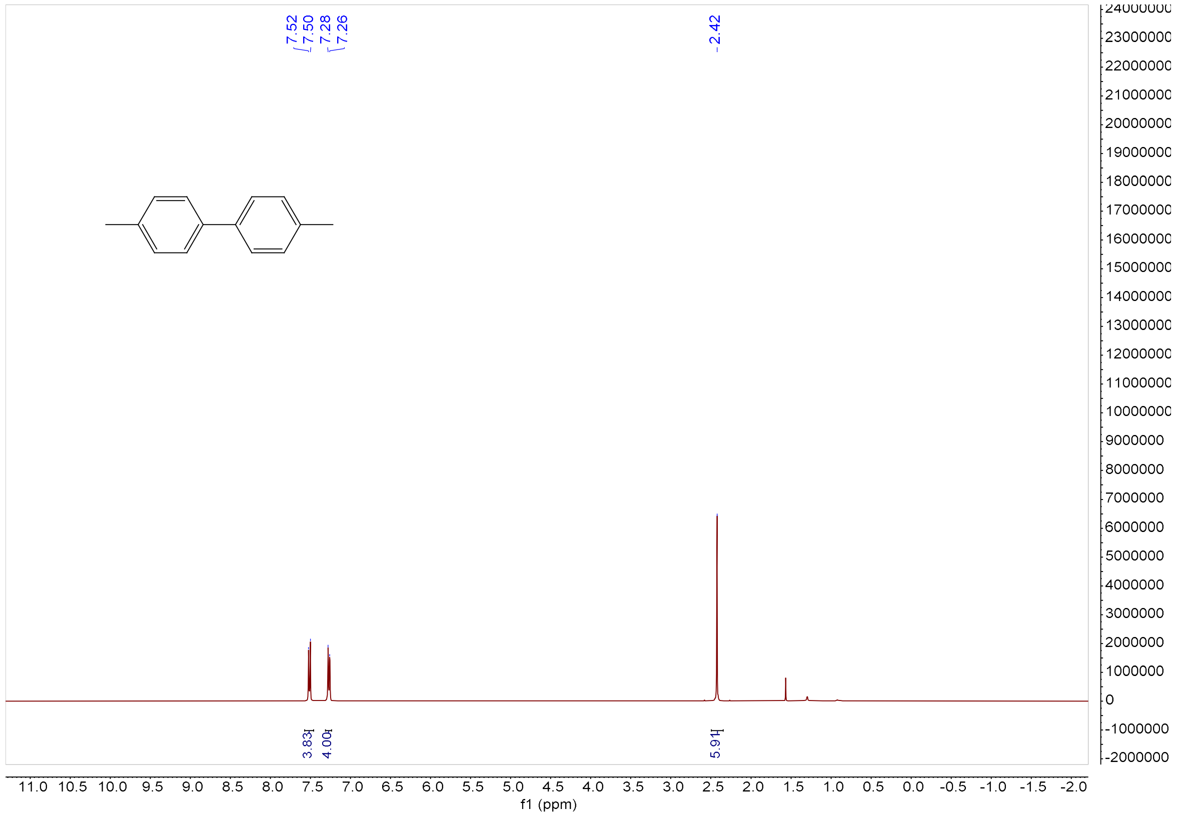

^{13}C NMR spectrum of compound **12da** (101 MHz, $CDCl_3$)

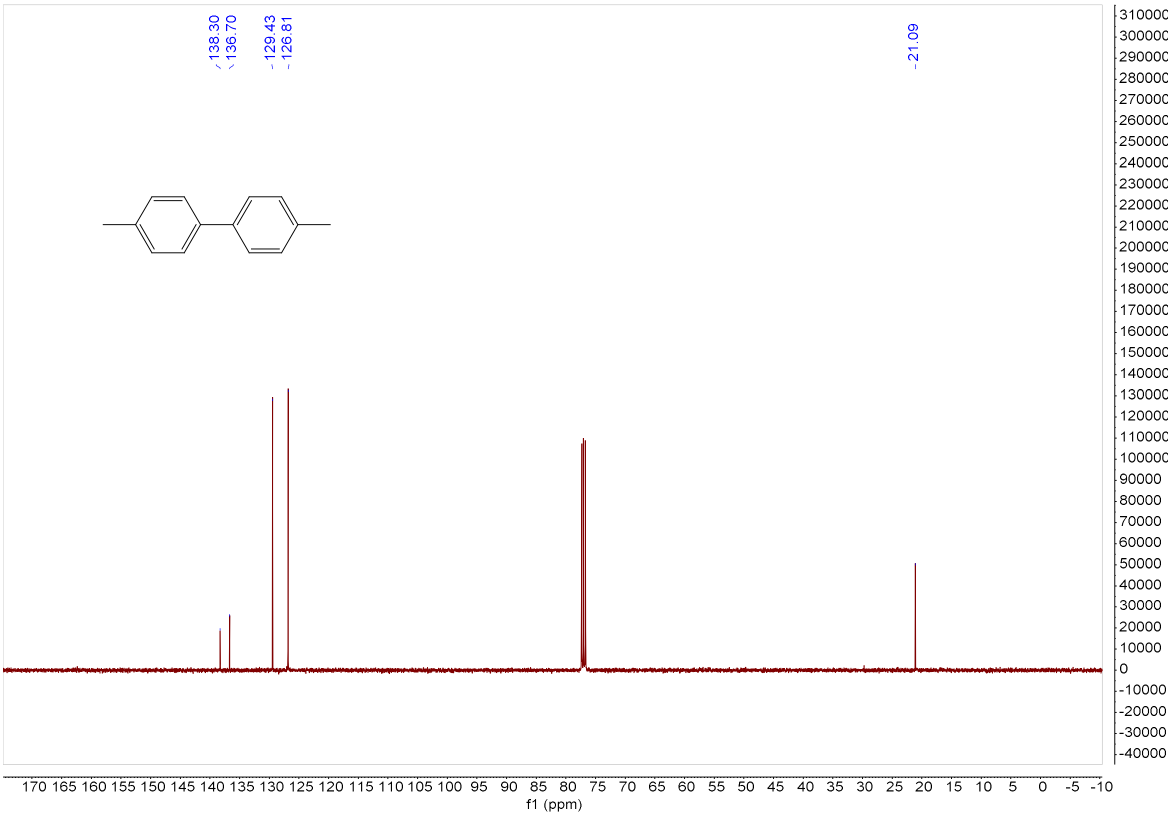

^{1}H NMR spectrum of compound **12fa** (600 MHz, $CDCl_3$)

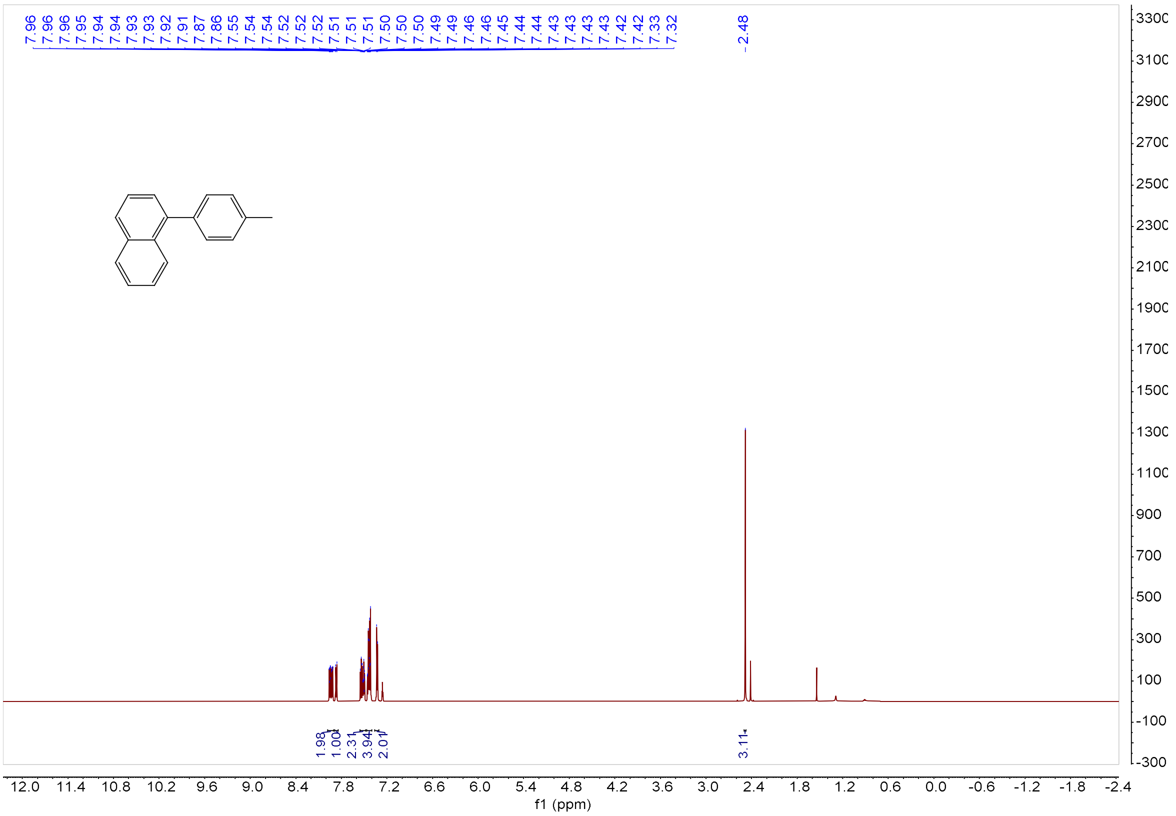

^{13}C NMR spectrum of compound **12fa** (151 MHz, $CDCl_3$)

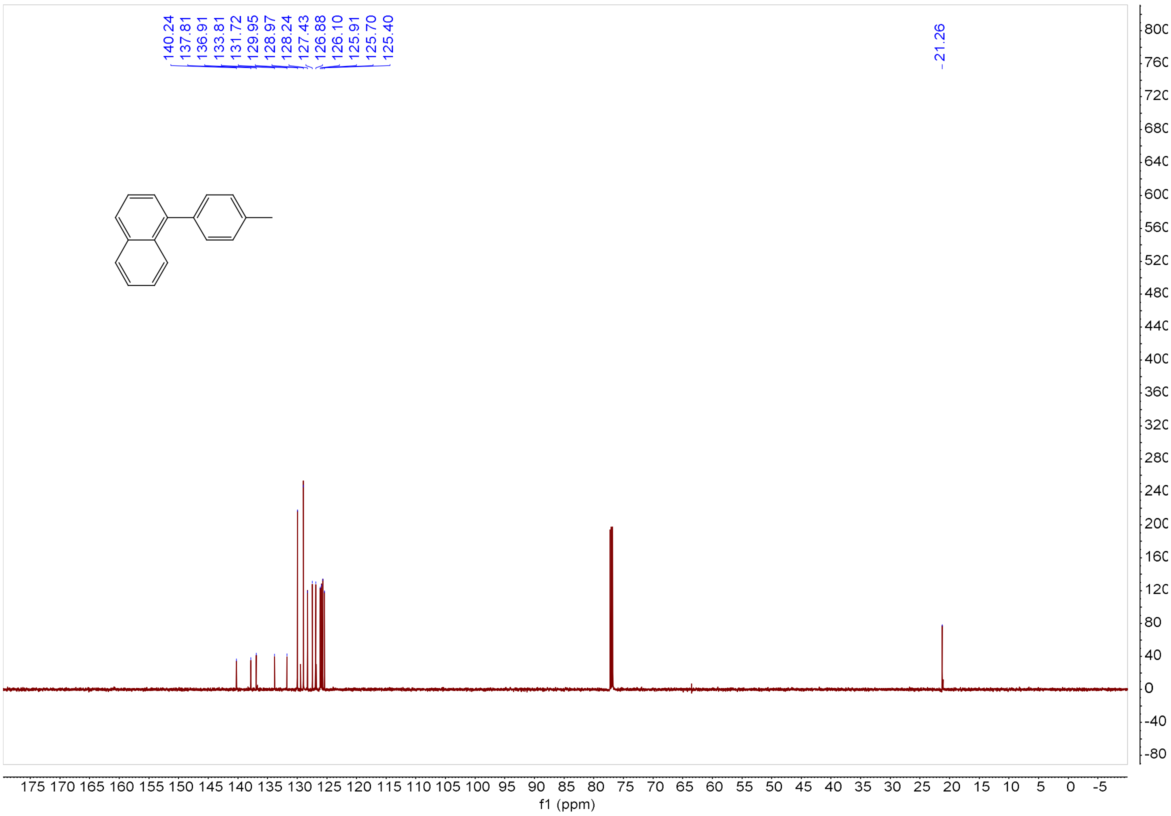

^{1}H NMR spectrum of compound **12bb** (600 MHz, $CDCl_3$)

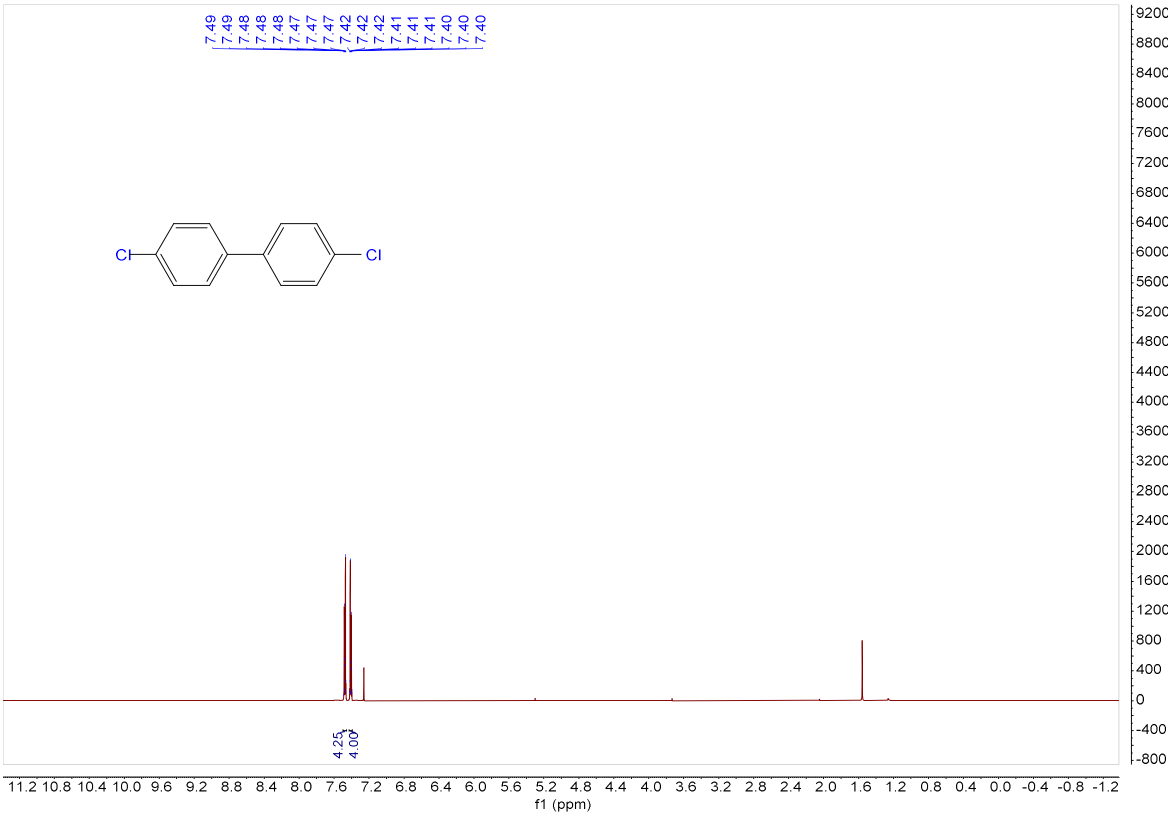

^{13}C NMR spectrum of compound **12bb** (151 MHz, $CDCl_3$)

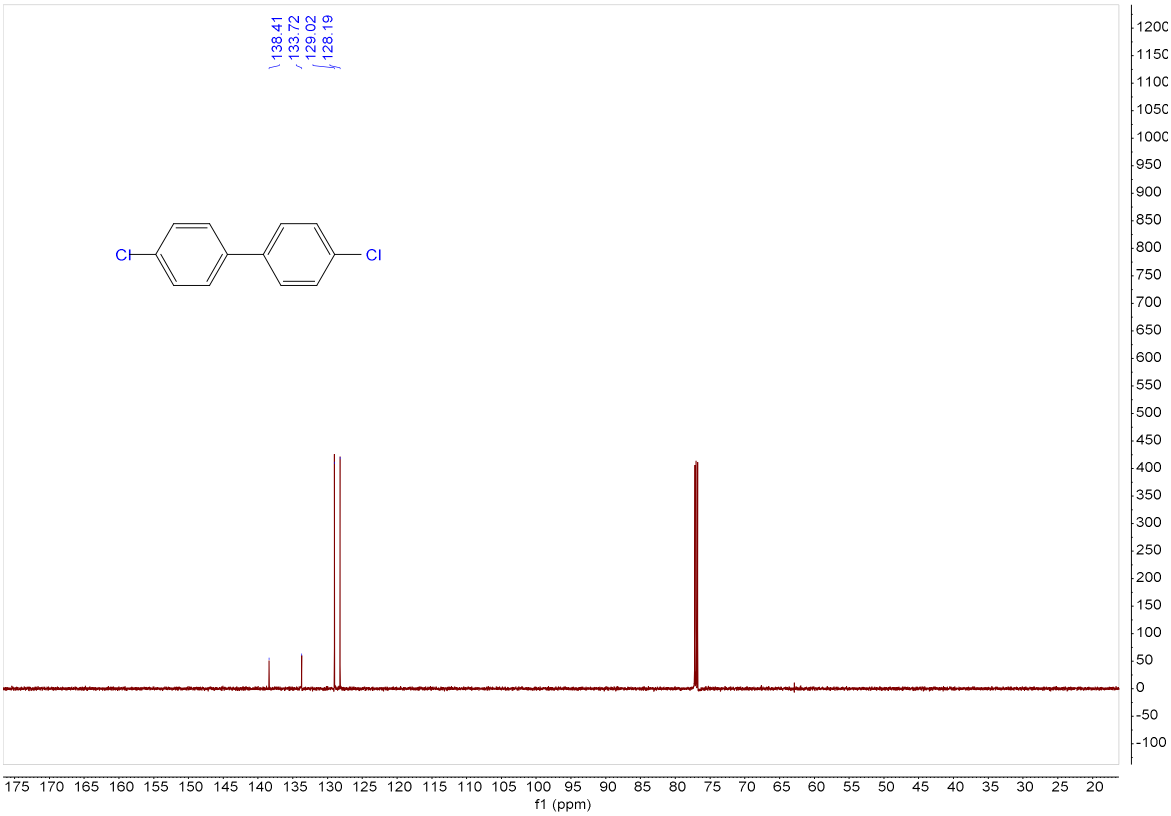

^{1}H NMR spectrum of compound **12bc** (600 MHz, $CDCl_3$)

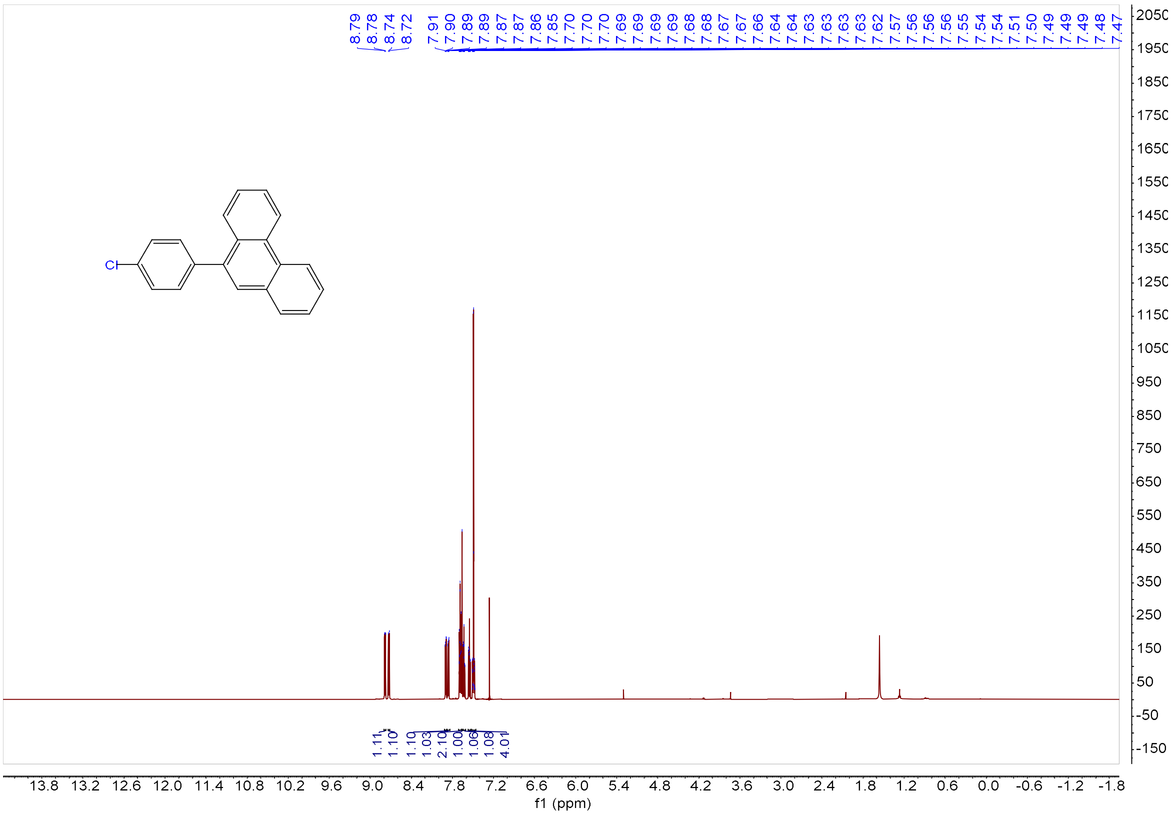

^{13}C NMR spectrum of compound **12bc** (151 MHz, $CDCl_3$)

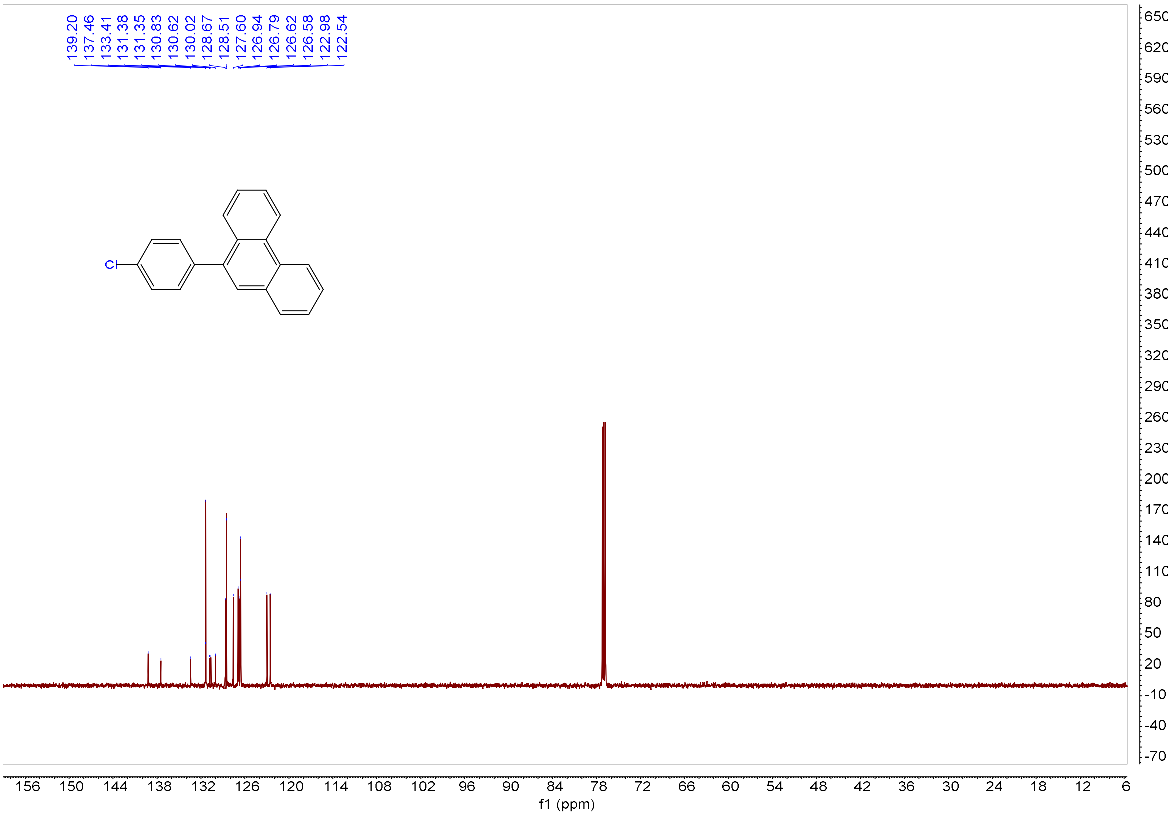

^{1}H NMR spectrum of compound **12ia** (600 MHz, $CDCl_3$)

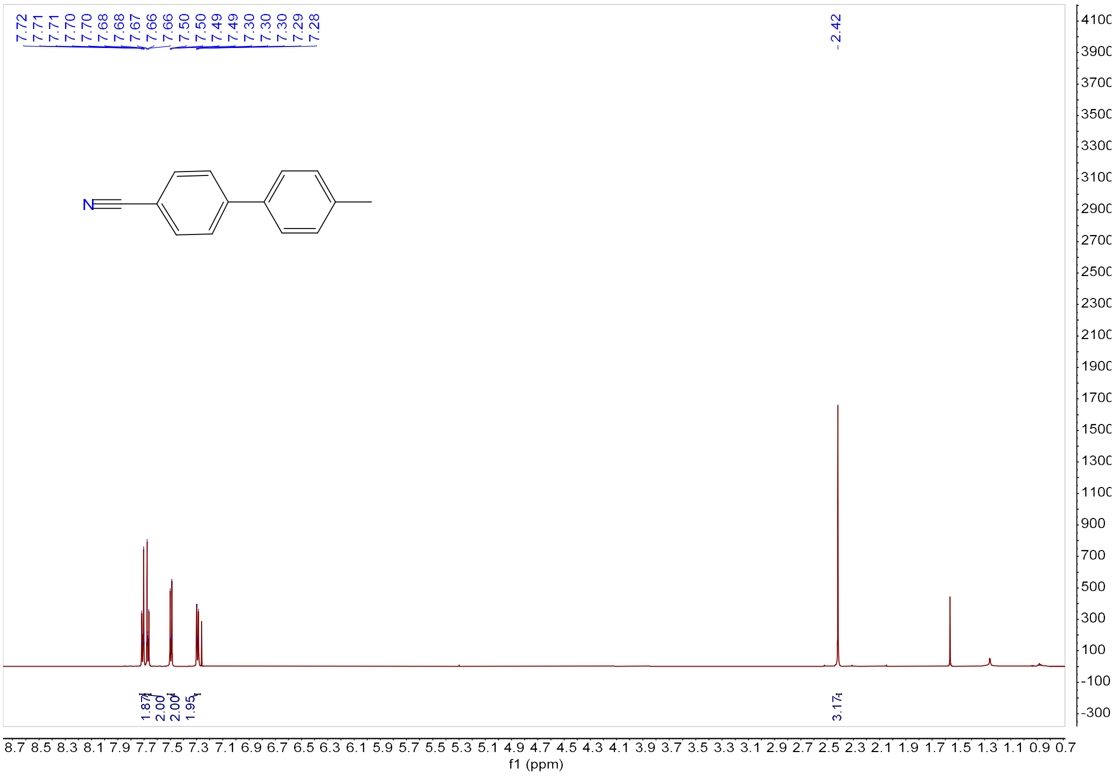

^{13}C NMR spectrum of compound **12ia** (151 MHz, $CDCl_3$)

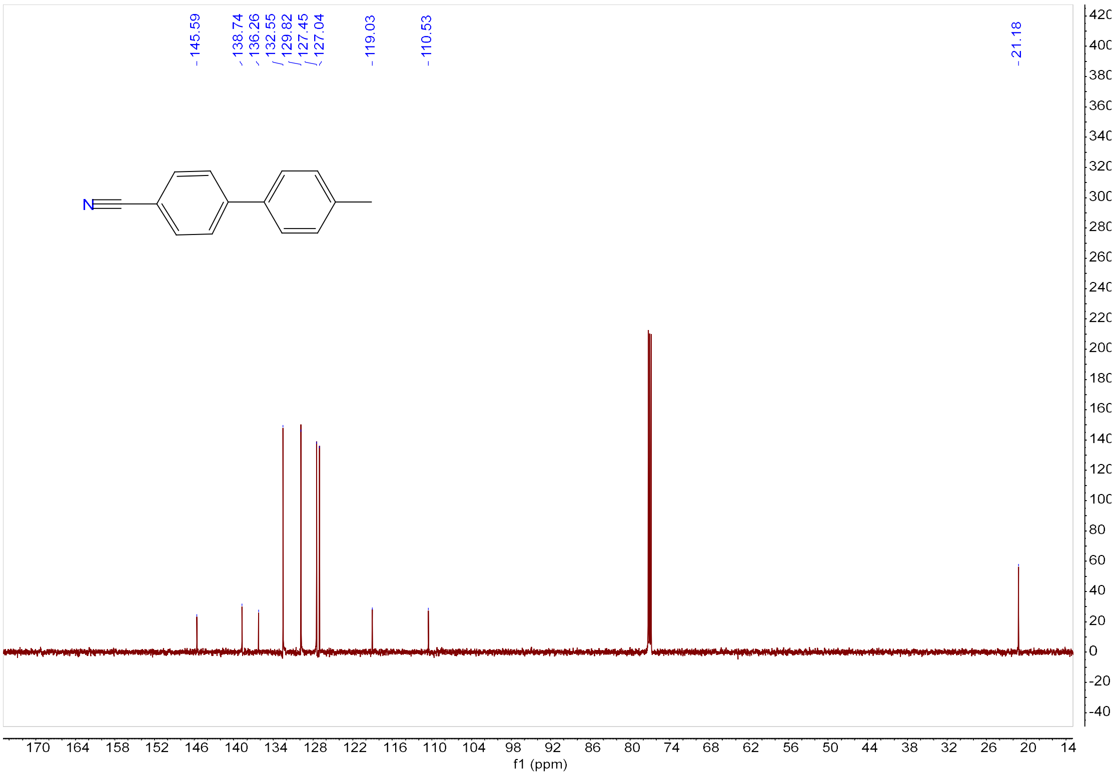

Supplement: Supplementary file 1 — Supporting Information [file ASIA-17-0-s001.pdf]
